# Resource availability and adjustment of social behaviour influence patterns of inequality and productivity across societies

**DOI:** 10.7717/peerj.5488

**Published:** 2018-10-02

**Authors:** António M.M. Rodrigues

**Affiliations:** 1 Department of Zoology, University of Cambridge, Cambridge, United Kingdom; 2 Wolfson College, Cambridge, United Kingdom

**Keywords:** Class structure, Cooperation, Reproductive value, Kin selection, Spatial and temporal variation, Resource availability, Kin competition, Demography, Heterogeneous populations

## Abstract

Animal societies vary widely in the diversity of social behaviour and the distribution of reproductive shares among their group members. It has been shown that individual condition can lead to divergent social roles and that social specialisation can cause an exacerbation or a mitigation of the inequality among group members within a society. This work, however, has not investigated cases in which resource availability varies between different societies, a factor that is thought to explain variation in the level of cooperation and the disparities in reproductive shares within each social group. In this study, I focus on how resource availability mediates the expression of social behaviour and how this, in turn, mediates inequality both within and between groups. I find that when differences in resource availability between societies persist over time, resource-rich societies become more egalitarian. Because lower inequality improves the productivity of a society, the inequality between resource-rich and resource-poor societies rises. When resource availability fluctuates over time, resource-rich societies tend to become more unequal. Because inequality hinders the productivity of a society, the inequality between resource-rich and resource-poor societies falls. From the evolutionary standpoint, my results show that spatial and temporal variation in resource availability may exert a strong influence on the level of inequality both within and between societies.

## Introduction

Animal societies display a wide range of variation in the distribution of the available reproductive resources among their members. In some species, the distribution of reproductive shares among group members is relatively equal (Cashdan, 1980; Cant, 2000; Packer, Pusey & Eberly, 2001; Flannery & Marcus, 2012; Scheidel, 2017). In others, this picture changes dramatically, and societies show pronounced reproductive inequality, with one or few individuals monopolising most of the resources of a society (Rood, 1986; Ellis, 1995; Boehm, 1999; Clutton-Brock et al., 2001; Charpentier et al., 2005; Surbeck et al., 2017). Explaining this variation in inequality across the animal world has been the object of much interest among behavioural ecologists, where a leading hypothesis views the social behaviour of group members as the main driver of inequality (Hamilton, 1964; Vehrencamp, 1983; West, Griffin & Gardner, 2007; Bourke, 2011; Davies, Krebs & West, 2012).

A large body of work has sought to understand adaptive variation in cooperation among species (Hamilton, 1964; West, Griffin & Gardner, 2007; Bourke, 2011), where relatedness and kin competition have been two key predictors commonly used to explain the evolution of social behaviour (Hamilton, 1964; West et al., 2001; West, Griffin & Gardner, 2007; Lehmann & Rousset, 2010; Bourke, 2011; Rodrigues & Kokko, 2016). High relatedness is required for the evolution of the extreme levels of altruism that characterise major evolutionary transitions in individuality (Hughes et al., 2008; Boomsma, 2009), and for the evolution of cooperatively breeding systems, both in birds (Cornwallis et al., 2010) and mammals (Lukas & Clutton-Brock, 2012). Kin competition can prevent the evolution of cooperation and even drive the evolution of conflict among closely related individuals (West et al., 2001; West, Pen & Griffin, 2002; Clutton-Brock et al., 2001). While these variables are able to explain average levels of cooperation across species, they are inadequate when the aim is to understand why specific individuals decide to adopt alternative behavioural and reproductive strategies (e.g., Clutton-Brock et al., 2010; Uematsu et al., 2010).

Alternative social strategies may emerge whenever individuals differ in life history traits (e.g., West-Eberhard, 1981; Craig, 1983; Frank, 1996; Frank, 2010; Markiewicz & O’Donnell, 2001; Whitlock, Davis & Yeaman, 2007; Rodrigues & Gardner, 2013a). Differences in individual condition can change the costs and benefits of alternative behaviours, and cause the evolution of contrasting behavioural strategies that ultimately shape the evolution of inequality. This hypothesis generates a simple relationship between the condition of individuals and the social and reproductive structure of groups. In natural populations, by contrast, individuals in identical condition need not adopt the same strategy, as the association between condition and social strategies can vary considerably within any given population (Nichols et al., 2012; Castillo, Queller & Strassmann, 2011; Flannery & Marcus, 2012; Cote et al., 2013). In fact, growing evidence suggests that the condition of individuals alone is insufficient to predict their behaviour. In the mosquitofish *Gambusia affinis*, for instance, personality-dependent behaviour strongly depends on the ecological context (Cote et al., 2013). In meerkats, the behaviour of dominant individuals varies significantly with their social environment (Clutton-Brock et al., 2010). Therefore, identifying additional key ecological and life history variables that explain not only why individuals adopt alternative strategies within a society, but also why these alternative strategies vary between societies remains a major challenge.

A leading hypothesis for explaining differences in the social and reproductive structure of societies is resource availability (e.g., Callaway et al., 2002; Castillo, Queller & Strassmann, 2011). Although the influence of resource availability on conflict and cooperation has been widely investigated, its impact on the social and reproductive composition of groups remains largely unexplored. In his classic book, *Mutual Aid*, Kropotkin (1902) pioneered the idea of resource availability as a primary force shaping the evolution of cooperation. Over the past few decades, resource availability has been central to theories concerning the evolution of cooperative breeding (Emlen, 1982; Stacey & Ligon, 1991; Jetz & Rubenstein, 2011), as well as the evolution of helping across many species (Covas, Du Plessis & Doutrelant, 2008; Krams et al., 2010).

However, little attention has been paid to understanding how resource availability influences variation in social behaviour and inequality within species. It is well-documented that the scarcity of local resources often leads high-status individuals to suppress the reproductive activity of social partners (e.g., Clutton-Brock et al., 2010; Guinote et al., 2015; Pettay et al., 2016). Under this assumption, Ellis (1995) proposed that in resource-rich societies, the competitive instincts of high-status individuals are contained so that abundant resources are expected to create a more egalitarian distribution of reproductive shares among social partners. However, empirical data seem to suggest a more complex pattern. While in some cases we see a negative correlation between resource availability and inequality (Callaway et al., 2002), in others we observe a positive correlation (Castillo, Queller & Strassmann, 2011; Nichols et al., 2012). This suggests that focusing on resource availability alone is insufficient to explain variation in the social and reproductive structure of groups.

A general limitation of these explanations is their premise that resource availability is constant over time. In natural populations, this is unlikely to be the case. Further, theoretical work shows that temporal variation in resource availability has a strong impact upon the adaptive expression of social behaviour (Rodrigues & Gardner, 2012; Rodrigues & Gardner, 2013b; Rodrigues & Gardner, 2016). Thus, one hypothesis to resolve the seemingly conflicting observations is that some populations may experience fluctuations in resource availability, while others may benefit from a constant supply of resources through time, and that this has an impact on both the social and reproductive structure of societies.

A wealth of models has considered the evolution of social behaviour in spatially structured populations where local competition for resources is a significant evolutionary force (Gardner & West, 2006;  Alizon & Taylor, 2008; Lion & Gandon, 2009; Powers & Lehmann, 2013; Débarre, Hauert & Doebeli, 2014; Peña, Nöldeke & Lehmann, 2015); for reviews see Lehmann & Rousset, 2010; Rodrigues & Kokko, 2016). Far fewer models have studied how variation in individual condition influences the evolution of social behaviour (e.g., Weesie, 1993; Frank, 1996; Frank, 2010; Whitlock, Davis & Yeaman, 2007; Bao & Wild, 2012; Przepiorka & Diekmann, 2013; Rodrigues & Gardner, 2013a; Rodrigues & Gardner, 2016). Frank (2010), for instance, has shown that better-endowed individuals contribute more to a public good than individuals with fewer resources, with the contrasting behaviours having an equalising effect within societies. Rodrigues & Gardner (2013a) found that those individuals with higher reproductive value contribute less to helping than those with lower reproductive value. However, Rodrigues & Gardner (2013a) did not study how the expression of condition-dependent behaviour is affected by variation in resource availability. Conversely, Rodrigues & Gardner (2012) studied the influence of spatial and temporal variation in resource availability on the evolution of average levels of helping. However, they did not study how variation in resource availability affects the evolution of condition-dependent behaviour and how this shapes the evolution of inequality. Thus, the relationship between resource availability and social behaviour remains unclear.

In this paper, I present a general kin selection model to investigate the relationship between temporal variation in resource availability, life history, social behaviour, and the evolution of inequality, both within and between groups. I assume that individuals differ in their intrinsic quality and that these differences have an impact on their fecundity or wealth. I describe how different ecological, demographic, and genetic variables influence the genetic structure of the population and the reproductive value of each individual. I then ask how these key variables mediate the fitness of each individual through their impact on the kin-selected benefits of resource transfer among individuals, and on the costs of kin competition arising from the scarcity of local resources. In addition, I study how these different factors influence the evolution of the individuals’ social behaviour, in general, and of the individuals’ competitive effort, in particular. Finally, I study how the evolved behaviours shape the differences between individuals, and whether this enhances or reduces initial levels of inequality among group members.

## Model

### Life-cycle

I assume a population of asexually-reproducing haploid individuals subdivided into a very large number of groups (as in Wright’s (1931) infinite island model), which I suppose vary in resource availability. Generations are non-overlapping, and following (Rodrigues & Gardner, 2012; Rodrigues & Gardner, 2016), I suppose that patches are of two types: high-quality resource-rich patches and low-quality resource-poor patches (although the resource availability of a patch may change from one generation to the next, as discussed below). Each patch contains a high-quality and a low-quality breeder (cf. Rodrigues & Gardner, 2013a; Rodrigues & Gardner, 2016). The fecundity of both breeders is assumed to be very large, albeit different. More specifically, in any given generation, the high-quality breeder (denoted by ‘H’) in a resource-rich patch (denoted by ‘R’) gives birth to *f*_HR_(*x*_HR_, *x*_LR_) offspring, while the low-quality (denoted by ‘L’) gives birth to *f*_LR_(*x*_LR_, *x*_HR_) offspring (where *f*_HR_(*x*_HR_, *x*_LR_) ≥ *f*_LR_(*x*_LR_, *x*_HR_). The fecundity of each breeder depends on the level of expression of a social behaviour by the high-quality individual (denoted *x*_HR_) and by the low-quality individual (denoted *x*_LR_). Equivalent fecundities and levels of expression of the social behaviour for individuals in a resource-poor patch (denoted by ‘P’) are denoted *f*_HP_(*x*_HP_, *x*_LP_) and *f*_LP_(*x*_LP_, *x*_HP_), *x*_HP_ and *x*_LP_. I describe the social behaviour in more detail below.

Following social interactions and reproduction, a fraction 1 – *d* of the offspring remain in their natal patch, while a fraction *d* disperse to a random patch in the population. Dispersers survive dispersal with probability 1 – *c*, where *c* is the mortality cost of dispersal. Offspring then compete for the available breeding vacancies created by the death of the adult generation. Offspring are identical in quality and therefore all have equal chances of becoming a high- or a low-quality breeder. Patches may then undergo changes in their resource availability. Resource-rich patches remain resource-rich with probability *p*_R→R_, and become resource-poor with probability *p*_R→P_ = 1 − *p*_R→R_; resource-poor patches remain resource-poor with probability *p*_P→P_, and become resource-rich with probability *p*_P→R_ = 1 − *p*_P→P_. After these ecological changes the life-cycle repeats. Model notation is summarised in Table 1.

**Table 1:** Summary of model notation.

**Symbol**	**Description**
*A*_*ασ*_	Potential for helping of a quality-*α* (or state-*α*) breeder in a type-*σ* patch
*c*	Cost of dispersal
*d*	Dispersal rate of a focal individual
*f*_*ρσ*_	Fecundity of a state-*ρ* individual in a type-*σ* patch
*F*_*σ*_	Total fecundity (i.e., productivity) in type-*σ* patches
*G*_*ω*,*σ*_	Inequality within type-*σ* patches
*G*_*β*_	Inequality between patch types
H (or L)	High-quality (or low-quality) individuals
*μ*_*σ*_	Resource availability in a type-*σ* patch
*p*_*σ*→*σ*′_	Probability that a type-*σ* patch becomes a type-*σ*′ patch
*q*_*ρσ*_	Baseline fecundity of a state-*ρ* breeder in a type-*σ* patch
*r*_*σ*_	Relatedness between social partners in type-*σ* patches
R (or P)	Resource-rich (or resource-poor) patches
*τ*	Coefficient of temporal correlation
*v*_*ασ*_ (or *V*_*ασ*_)	Reproductive value of a state-*α* breeder (or offspring) in a type-*σ* patch
${v}_{\alpha \sigma }^{\phi }$	Philopatric component of the reproductive value of a state-*α* breeder in a type-*σ* patch
*x*_*ρσ*_	Behaviour of a state-*ρ* individual in a type-*σ* patch
*z*_*ρσ*_	Population’s average behaviour of state-*ρ* individuals in type-*σ* patches

### Methodology

I am interested in the evolution of a social trait that is costly to the actor and is either helpful or harmful to the recipient. I assume (as stated above) that this trait is expressed at a level that is conditional upon an individual’s state (i.e., its quality, i.e., ‘H’ or ‘L’, and the resource availability of its patch, i.e., ‘R’ or ‘P’). I use the neighbour-modulated approach to kin selection (Taylor & Frank, 1996; Frank, 1998; Rousset, 2004; Rousset & Ronce, 2004; Rodrigues & Gardner, 2013a) to derive the inclusive fitness effect of the trait of interest.

The inclusive fitness effect of the trait of interest depends on two key quantities: relatedness and reproductive value. Relatedness gives a measure of the value of social partners pertaining to the degree to which they share genes in common (Hamilton, 1964). Reproductive value gives a measure of the value of social partners pertaining to the degree to which they are able to project copies of their genes into the gene pool of future generations (Fisher, 1930;  Taylor, 1990; Grafen, 2006). I then conceptualise the fitness effect of the trait in terms of Hamilton’s rule, which gives the condition for the evolution of an alternative allele (Hamilton, 1964; Charnov, 1977). Finally, given Hamilton’s rule, I can obtain evolutionarily stable levels of the traits of interest, which occur whenever the left-hand side (LHS) of Hamilton’s rule is zero, and therefore there is selection neither for an increase nor a decrease in trait value. To solve the model, I use a combination of analytical and numerical methods, a detailed description of which can be see in the Appendix S1.

First, I determine the relatedness coefficients among group members. For this purpose, I define a set of recursion equations describing changes in the genetic structure of groups from one generation to the next (Taylor, 1992; Rousset, 2004; Rodrigues & Gardner, 2012; Rodrigues & Gardner, 2016). More specifically, the relatedness coefficients depend on the state of the patch in the previous generation, i.e., the probability *p*_*γ*|*σ*_, that a patch currently in state *σ* was in state *γ* in the previous generation; the probability *φ*_*γ*_ that both adults are philopatric; the probability *U*_H*γ*_^2^ (or ${U}_{\mathrm{L}\gamma }^{2}$) that they are both offspring of a high-quality (or low-quality) breeder; and the probability 2*U*_H*γ*_*U*_L*γ*_ that one is an offspring of a high-quality breeder and the other is an offspring of a low-quality breeder. If they are siblings, their relatedness is one, otherwise their relatedness is *r*_*γ*_, with *γ* ∈{*R*, *P*}. Collecting these terms together, we obtain the following recursion equations

(1) \begin{eqnarray*}{r}_{\sigma }^{{^{\prime}}}=\sum _{\gamma \in \{\mathrm{R},\mathrm{P}\}}{p}_{\gamma {|}\sigma }{\varphi }_{\gamma } \left( {U}_{\mathrm{H}\gamma }^{2}+{U}_{\mathrm{ L}\gamma }^{2}+2{U}_{\mathrm{ H}\gamma }{U}_{\mathrm{L}\gamma }{r}_{\gamma } \right) , \sigma \in \left\{ \mathrm{R},\mathrm{P} \right\} ,\end{eqnarray*}

which can be solved for equilibrium (i.e., ${r}_{\sigma }^{{^{\prime}}}={r}_{\sigma }$), in order to derive the relatedness coefficients in each patch type (see Appendix S1 for details).

Second, I determine the reproductive value of breeders (denoted by *v*), where reproductive value is defined as the contribution of a breeder to the gene pool of future generations (Fisher, 1930; Grafen, 2006). The reproductive value of a breeder is given by the elements of the left eigenvector associated to the leading eigenvalue of the fitness matrix **w**, which is

(2) \begin{eqnarray*}\mathbf{w}={ \left( {w}_{\rho \pi \longrightarrow \eta \gamma } \right) }_{4\times 4}.\end{eqnarray*}

Each element of this matrix denotes the number of offspring of a focal breeder that secure a breeding place in the next generation, according to the state of the breeder (i.e., her quality *ρ*, and her patch type *π*), and according to the breeding state of her offspring (i.e., the offspring quality *η*, and the offspring patch type *γ*; see Appendix S1 for details).

Third, I determine the neighbour-modulated fitness of a focal breeder of each possible state. To do this, I sum the number of successful offspring the focal breeder has, weighting each successful offspring by its reproductive value. This weighted total is then divided by the reproductive value of a random breeder in the focal class, yielding

(3) \begin{eqnarray*}{W}_{\rho \pi }= \frac{\sum _{\gamma \in \{\mathrm{R},\mathrm{P}\}}\sum _{\eta \in \{\mathrm{H},\mathrm{L}\}}{w}_{\rho \pi \longrightarrow \eta \gamma }{v}_{\eta \gamma }}{{v}_{\rho \pi }} .\end{eqnarray*}

Fourth, I determine the expected fitness of a randomly chosen focal breeder, who might be of any state. This is given by

(4) \begin{eqnarray*}\overline{w}=\sum _{\pi \in \{\mathrm{R},\mathrm{P}\}}\sum _{\rho \in \{\mathrm{H},\mathrm{L}\}}{u}_{\rho \pi }{v}_{\rho \pi }{W}_{\rho \pi },\end{eqnarray*}

where *u*_*ρ*__*π*_ is the frequency of individuals in state *ρ* (i.e., either high- or low-quality) and in patch type *π* (i.e., either resource-rich or resource-poor), which is given by the right eigenvector associated to the leading eigenvalue of matrix **w** (see Appendix S1 for details). Finally, the fitness effect of a social behaviour enacted by a focal actor in state *α* in a patch with resource availability *σ* is given by the slope of an individual’s expected fitness ($\overline{w}$) on its breeding value for that level of expression (*g*_*ασ*_). This is given by

(5) \begin{eqnarray*} \frac{\mathrm{d}\overline{w}}{\mathrm{d}{g}_{\alpha \sigma }} =\sum _{\pi \in \{\mathrm{R},\mathrm{P}\}}\sum _{\rho \in \{\mathrm{H},\mathrm{L}\}}{u}_{\rho \pi }{v}_{\rho \pi } \frac{\mathrm{d}{W}_{\rho \pi }}{\mathrm{d}{g}_{\alpha \sigma }} .\end{eqnarray*}

Note that social interactions unfold among individuals in the same patch, and therefore a behaviour expressed in resource-rich patches (or in resource-poor patches) does not affect individuals in resource-poor patches (or in resource-rich patches). Thus, in Eq. (5), the slope of fitness of individuals in resource-rich patches (or in resource-poor patches) on the breeding value of individuals in resource-poor patches (or in resource-rich patches) is zero.

### Hamilton’s rule

If the fitness effect, given by Eq. (5), is greater than zero (i.e., $\mathrm{d}\overline{w}/\mathrm{d}{g}_{\alpha \sigma }\gt 0$), then a slightly higher value of the trait evolves, a condition that is often known as Hamilton’s rule (Hamilton, 1964; Charnov, 1977; Rodrigues & Gardner, 2013a). Hamilton’s rule governing the behaviour of a quality-*α* breeder in a type-*σ* patch is given by

(6) \begin{eqnarray*}-\underbrace{{C}_{\alpha \sigma }{V}_{\alpha \sigma }{}}_{ {\text{primary}\atop \text{cost}} }+\underbrace{{B}_{\alpha \sigma }{V}_{\rho \sigma }{r}_{\sigma }{}}_{ {\text{primary}\atop \text{benefit}} }-\underbrace{ \left( {B}_{\alpha \sigma }-{C}_{\alpha \sigma } \right) {w}_{\sigma }^{\phi } \left( {v}_{\alpha \sigma }^{\phi }+{v}_{\rho \sigma }^{\phi }{r}_{\sigma } \right) {}}_{\text{kin competition cost}}\gt 0,\end{eqnarray*}

where: *C*_*ασ*_ is the fecundity cost paid by the quality-*α* actor in the type-*σ* patch; *V*_*ασ*_ (or *V*_*ρσ*_) is the reproductive value of the actor’s (or recipient’s) offspring; *B*_*ασ*_ is the benefit enjoyed by the recipient; *r*_*σ*_ is the relatedness between the actor and recipient; ${w}_{\sigma }^{\phi }$ is the probability that a native random offspring remains and wins a breeding site in the focal type-*σ* patch; and ${v}_{\alpha \sigma }^{\phi }$ (or ${v}_{\rho \sigma }^{\phi }$) is the philopatric component of the actor’s (or recipient’s) expected reproductive value (i.e., the reproductive value derived from offspring that remain in the local patch). In the Appendix S1, I provide additional information on how to calculate these quantities.

Hamilton’s rule immediately yields an inclusive fitness interpretation of the behaviour, which can be partitioned into a primary cost and benefit of the behaviour and a kin competition cost. First, the actor’s behaviour costs her *C*_*ασ*_ offspring, with each offspring representing a decrement *V*_*ασ*_ in her reproductive value. Second, the actor’s behaviour improves the fecundity of the recipient by an amount *B*_*ασ*_, with each additional offspring representing an increment *V*_*ρσ*_ in reproductive value, an increment that must be depreciated by the relatedness *r*_*σ*_ between social partners. Finally, the behaviour leads to the production of *B*_*ασ*_ − *C*_*ασ*_ additional offspring, with a fraction ${w}_{\sigma }^{\phi }$ of these offspring remaining and displacing other offspring in the local patch. The displacement of offspring destroys a portion ${v}_{\alpha \sigma }^{\phi }$ of the actor’s reproductive value, and a portion ${v}_{\rho \sigma }^{\phi }$ of the partner’s reproductive value, where the partner’s portion must be depreciated by the coefficient of relatedness *r*_*σ*_.

Hamilton’s rule gives the condition for the evolution of a social trait, which is either a helping trait when the benefit is greater than zero (i.e., *B*_*ασ*_ > 0), or a harming trait when the benefit is less than zero (i.e., *B*_*ασ*_ < 0). If Hamilton’s rule holds, then the mutant expressing a slightly higher value of the trait spreads to fixation. To analyse the action of kin selection, one can set the left-hand side of Hamilton’s rule to zero, and re-arrange the resulting equation in the form of *C*_*ασ*_∕*B*_*ασ*_ = *A*_*ασ*_. The variable ${A}_{\alpha \sigma }=({r}_{\sigma }{V}_{\rho \sigma }-{w}_{\sigma }^{\phi }({v}_{\alpha \sigma }^{\phi }+{v}_{\rho \sigma }^{\phi }{r}_{\sigma }))/({V}_{\alpha \sigma }-{w}_{\sigma }^{\phi }({v}_{\alpha \sigma }^{\phi }+{v}_{\rho \sigma }^{\phi }{r}_{\sigma }))$ is the potential for helping (Rodrigues & Gardner, 2013a). Helping evolves if the potential for helping is positive (*A*_*ασ*_ > 0), whilst harming evolves if the potential for helping is negative (*A*_*ασ*_ < 0), providing the cost (*C*_*ασ*_) is sufficiently small. Of particular interest for the analysis is the coefficient of temporal correlation in resource availability (denoted by *τ*). At one extreme, when the coefficient of temporal correlation is 1 (i.e., *τ* = 1), the amount of locally available resources remains constant from one generation to the next. At another extreme, when the coefficient of temporal correlation is −1 (i.e., *τ* = − 1), the amount of locally available resources fluctuates from one generation to the next. The potential for helping and the other model variables depend on the coefficient of temporal correlation, as we shall see below.

### Competitive effort

Above, we have seen how to analyse the action of kin selection in terms of ratios of costs and benefits, which I have assumed constant. More generally, a behaviour will influence the relative fecundity of each actor, and this in turn will influence the variables, such as relatedness and reproductive value, that mediate the action of kin selection. Thus, to understand the level of helping and harming at evolutionary equilibrium, we need to specify how behaviour and fecundity are intertwined.

The particular behavioural function specifying the fecundity *f*_*ρσ*_ of a focal individual in state *ρ* in a type-*σ* patch will determine both the costs and the benefits of the behaviour. Let *x*_*ρσ*_ be the phenotype of a focal individual in state *ρ* in a focal type-*σ* patch, and ***x***_*σ*_ the vector of phenotypes of all individuals in the focal group, in a population where the average trait values are given by *z*_*ρσ*_. I can then specify that social interactions mediate the fecundity of the focal individual through a personal component Ψ_*ασ*_, which is a function of the focal individual’s phenotype *x*_*ασ*_, and a group component Θ_*σ*_, which is a function of the phenotype of all group members, as defined by ***x***_*σ*_. If the two components, personal and group, are multiplicative, then the fecundity cost of the behaviour is determined by the additive inverse of the effect of the focal individual’s phenotype on the personal component of its fecundity, i.e., *C*_*ασ*_ =  − (∂Ψ_*ασ*_∕∂*x*_*ασ*_)Θ_*σ*_. That is, an increase in the focal individual’s phenotype will allow her to acquire a larger share of the resources available in the local patch. Moreover, the fecundity benefit is determined by the effect of the actor’s phenotype on the group component of the focal individual’s fecundity, i.e., *B*_*ασ*_ = (∂Θ_*σ*_∕∂*x*_*ασ*_)Ψ_*ασ*_. That is, an increase in the focal individual’s phenotype also causes a decrease in the amount of resources available in the local patch.

Here, I focus on a particular form of social interactions, where *x*_*ασ*_ is defined as the competitive effort of a focal individual (c.f. Frank, 1994). In the appendix, I explore alternative behavioural functions, which, as we shall see below, give the same qualitative results (Appendix S1). I assume that competitive effort increases the rate or efficiency of resource acquisition by an individual, but decreases the total amount of resources available to the group. We can interpret greater competition as a harming (selfish) strategy because an individual gains a direct benefit at a cost to her social partner; while we can interpret reduced competition as a helping (cooperative) strategy because an individual pays a direct cost to provide a benefit to her group mate. I assume that the quality of individuals, denoted by *q*_*ρσ*_, varies, with high-quality individuals holding an intrinsic fecundity advantage relative to low-quality individuals. I assume that social interactions have an additive effect on the baseline fecundity of individuals, with social interactions either amplifying or alleviating the initial inequality between individuals. Furthermore, I assume that there are more resources available in resource-rich patches than in resource-poor patches. Thus, *μ*_R_ > *μ*_P_, where *μ*_R_ (or *μ*_P_) is the amount of resource in resource-rich (or resource-poor) patches. The fecundity function of a focal class-*ρ* individual in a type-*σ* patch is then given by

(7) \begin{eqnarray*}{f}_{\rho \sigma }={\mu }_{\sigma } \left( {q}_{\rho \sigma }+ \frac{{x}_{\rho \sigma }}{\sum _{\eta \in \{\mathrm{H},\mathrm{L}\}}{x}_{\eta \sigma }/{n}_{\sigma }} \left( 1-\sum _{\eta \in \{\mathrm{H},\mathrm{L}\}} \frac{{x}_{\eta \sigma }}{{n}_{\sigma }} \right) \right) ,\end{eqnarray*}

where Ψ_*ασ*_ = *x*_*ρσ*_, ${\Theta }_{\sigma }= \left( 1-{\sum }_{\eta \in \{\mathrm{H},\mathrm{L}\}}{x}_{\eta \sigma }/{n}_{\sigma } \right) /{\sum }_{\eta \in \{\mathrm{H},\mathrm{L}\}}{x}_{\eta \sigma }/{n}_{\sigma }$, and *n*_*σ*_ is the number of individuals in the patch, which in the model is two across all patches. My aim is to determine the optimal competitive effort for high- and low-quality individuals in resource-rich and in resource-poor patches (i.e., ${z}_{\mathrm{HR}}^{\mathrm{\ast }}$, and ${z}_{\mathrm{LP}}^{\mathrm{\ast }}$; and ${z}_{\mathrm{HP}}^{\mathrm{\ast }}$, and ${z}_{\mathrm{LP}}^{\mathrm{\ast }}$). These are the competitive efforts at which the LHS of Hamilton’s rule for each trait is neither positive nor negative, and which I check for convergence stability (Christiansen, 1991; Eshel, 1996; Taylor, 1996; see Appendix S1 for details), where convergence stability means that the evolutionary trajectories of the traits under selection will always converge towards the singular strategies. While I have not tested the convergence stability of the general model, I have found no instances where the singular strategies fail the convergence stability test. This suggests that the singular strategies of the specific competitive effort behavioural functions presented above are always convergence stable.

### Coefficients of inequality

Above, I have described how fecundity may vary both within and between groups. Here, I define the coefficients of inequality (denoted by *G*) to provide an operational measure of variation in fecundity both within and between groups. First, I define the coefficients of inequality within resource-rich and resource-poor patches, and I then define the coefficient of inequality between resource-rich and resource-poor patches.

The coefficient of within-group inequality, denoted by *G*_*ω*,*σ*_, is defined as *G*_*ω*,*σ*_ = 1 − *f*_L*σ*_∕*f*_H*σ*_, with *σ* ∈ {*R*, *P*}. Thus, when both breeders produce an equal number of offspring (i.e., *f*_L*σ*_ = *f*_H*σ*_), the group is strictly egalitarian and the coefficient of within-group inequality is zero (i.e., *G*_*ω*,*σ*_ = 0). By contrast, when the fecundity of low-quality individuals is negligible relative to the fecundity of high-quality individuals (i.e., *f*_L*σ*_ ≪ *f*_H*σ*_), the group is extremely unequal and the coefficient of within-group inequality is one (i.e., *G*_*ω*,*σ*_ = 1). The coefficient of between-group inequality, denoted by *G*_*β*_, is defined as *G*_β_ = 1 − *F*_L_∕*F*_H_, where *F*_*σ*_ = *f*_L*σ*_ + *f*_H*σ*_ is the productivity of type-*σ* patches. If the productivity of the two patch types is identical (i.e., *F*_L_ = *F*_H_), then between-group inequality is zero (i.e., *G*_*β*_ = 0). By contrast, if the productivity of resource-poor patches is negligible in relation to the productivity of resource-rich patches (i.e., *F*_L_ ≪ *F*_H_), then between-group inequality is one (i.e., *G*_*β*_ = 1).

Finally, I specify the baseline levels of both within-group inequality, denoted by ${G}_{\omega ,\sigma }^{0}$, and between-group inequality, denoted by ${G}_{\beta }^{0}$, by excluding the effects of social interactions. That is, I calculate the initial levels of inequality assuming fully selfish individuals (i.e., *x*_*ησ*_ = *z*_*ησ*_ = 1). Thus, from Eq. (7), I find that the initial level of within-group inequality is given by ${G}_{\omega ,\sigma }^{0}=1-{\mu }_{\sigma }{q}_{\mathrm{L}\sigma }/{\mu }_{\sigma }{q}_{\mathrm{H}\sigma }$, while the initial level of between-group inequality is given by ${G}_{\beta }^{0}=1-{\mu }_{\mathrm{L}} \left( {q}_{\mathrm{HL}}+{q}_{\mathrm{LL}} \right) /{\mu }_{\mathrm{H}} \left( {q}_{\mathrm{HH}}+{q}_{\mathrm{LH}} \right) $. The difference between initial and evolved levels of inequality gives the effect of social interactions on inequality, as we shall see below.

## Analysis and Results

I first consider selection for helping and harming behaviour in resource-rich and then in resource-poor patches. I then study how selection drives the evolution of optimal competitive effort strategies.

### Behaviour in resource-rich patches

**Figure 1:**
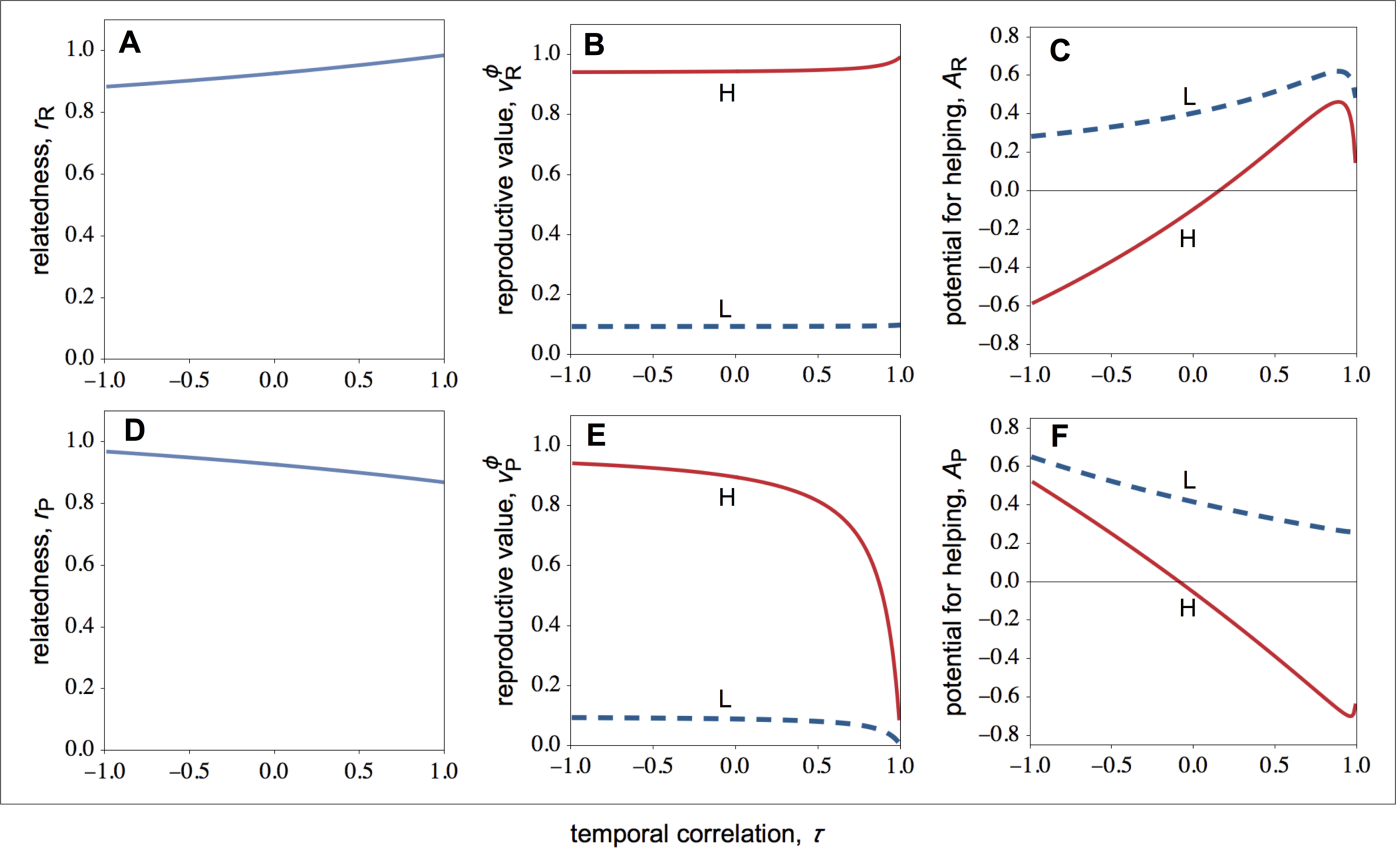
Relatedness, the philopatric component of reproductive value, and the potential for helping as a function of the temporal correlation for rich and poor patches. Relatedness (*r*), the philopatric component of reproductive value (*v*^*ϕ*^), and the potential for helping (*A*) of high- (solid lines) and low-quality (dashed lines) individuals as a function of the temporal correlation (*τ*) for rich and poor patches. (A–C) In resource-rich patches, temporal variation in resource availability decreases the potential for helping. (D–F) In resource-poor patches, temporal variation in resource availability increases the potential for helping. Parameter values: *c* = 0.9, *d* = 0.1, *p* = 0.5, *f*_*LR*_∕*f*_*HR*_ = *f*_*LP*_∕*f*_*HP*_ = 0.1, *F*_*P*_∕*F*_*R*_ = 0.1.

Let us first focus on how selection shapes the behaviour of individuals in resource-rich patches. As shown in Fig. 1C, low-quality individuals always help more than high-quality individuals. If we look at Fig. 1A, we see that both high- and low-quality breeders value offspring of the social partner equally (same *r*_H_). However, as shown in Fig. 1B, the intensity of kin competition experienced by high-quality breeders is higher than the intensity of kin competition experienced by low-quality breeders. More specifically, because high-quality breeders produce more offspring, high-quality breeders have more offspring competing for the available breeding sites than low-quality breeders. Hence, ${v}_{\mathrm{HR}}^{\phi }$ is relatively higher than ${v}_{\mathrm{LR}}^{\phi }$. As a result, the high-quality breeder is selected to suppress the reproduction of her low-quality partner to improve the chances that her philopatric offspring obtain a breeding site. By contrast, the low-quality breeder is selected to raise offspring of the high-quality social partner, as the low-quality breeder experience relatively lower kin competition costs.

Looking again at Fig. 1C, we see that average helping peaks when the environment is relatively stable (*τ* ≈ 1). More specifically, we find that selection for helping initially increases as the environment becomes unstable, but it rapidly starts to decrease as the instability of the environment increases further. The decrease in selection for helping with environmental instability is explained by a monotonic decrease in relatedness with environment instability (Fig. 1A). That is, as the environment becomes unstable, the probability that the focal resource-rich patch was a resource-poor patch increases, and therefore the probability that unrelated immigrants from resource-rich patches (rather than related philopatric individuals) recolonize the focal patch increases. The peak in the selection for helping, by contrast, is explained because the intensity of kin competition does not fall linearly with temporal instability. In particular, the philopatric component of reproductive value shows an initial sharp decline with temporal instability (Fig. 1B), which explains the initial increase in selection for helping with temporal instability.

### Behaviour in resource-poor patches

Let us now turn the attention to the action of selection in resource-poor patches. As shown in Fig. 1F, low-quality individuals always help more than high-quality individuals (a pattern also observed in resource-rich patches). Again, this is because low-quality individuals experience less kin competition than high-quality individuals (i.e., ${v}_{\mathrm{HP}}^{\phi }\gt {v}_{\mathrm{LP}}^{\phi }$; Fig. 1E).

Figure 1F also shows that the average helping peaks when the environment is relatively unstable (*τ* ≪ 1); specifically: in unstable environments, both high- and low-quality individuals help more; in stable environments, high-quality individuals harm low-quality individuals, and low-quality individuals help high-quality individuals less than in unstable environments. This is because both relatedness and the intensity of kin competition depend on the coefficient of temporal stability (Figs. 1D and 1F). In unstable environments, group mates are more likely to be close relatives because the probability that the focal patch was resource-rich in the previous generation is higher, and therefore the probability that the two breeders are both philopatric is also higher. On the other hand, there is a disproportional large influx of unrelated immigrants arriving at the local patch, which erodes the intensity of kin competition. Thus, unstable environments tend to favour the immediate fitness returns generated by helping behaviour among group mates, even if this gives rise to some additional amount of kin competition. In stable environments, group mates are relatively more likely to be unrelated because of low productivity in the local patch over consecutive generations, which means that the focal patch has been colonised by successive generations of unrelated immigrant individuals. On the other hand, if good fortune strikes, the local patch may become resource-rich, and therefore the next generation of resident individuals may be relatively more successful than the current generation. Thus, stable environments tend to favour lower helping among group mates, such that local offspring are more likely to win breeding spots in the next generation.

### Competitive effort

**Figure 2:**
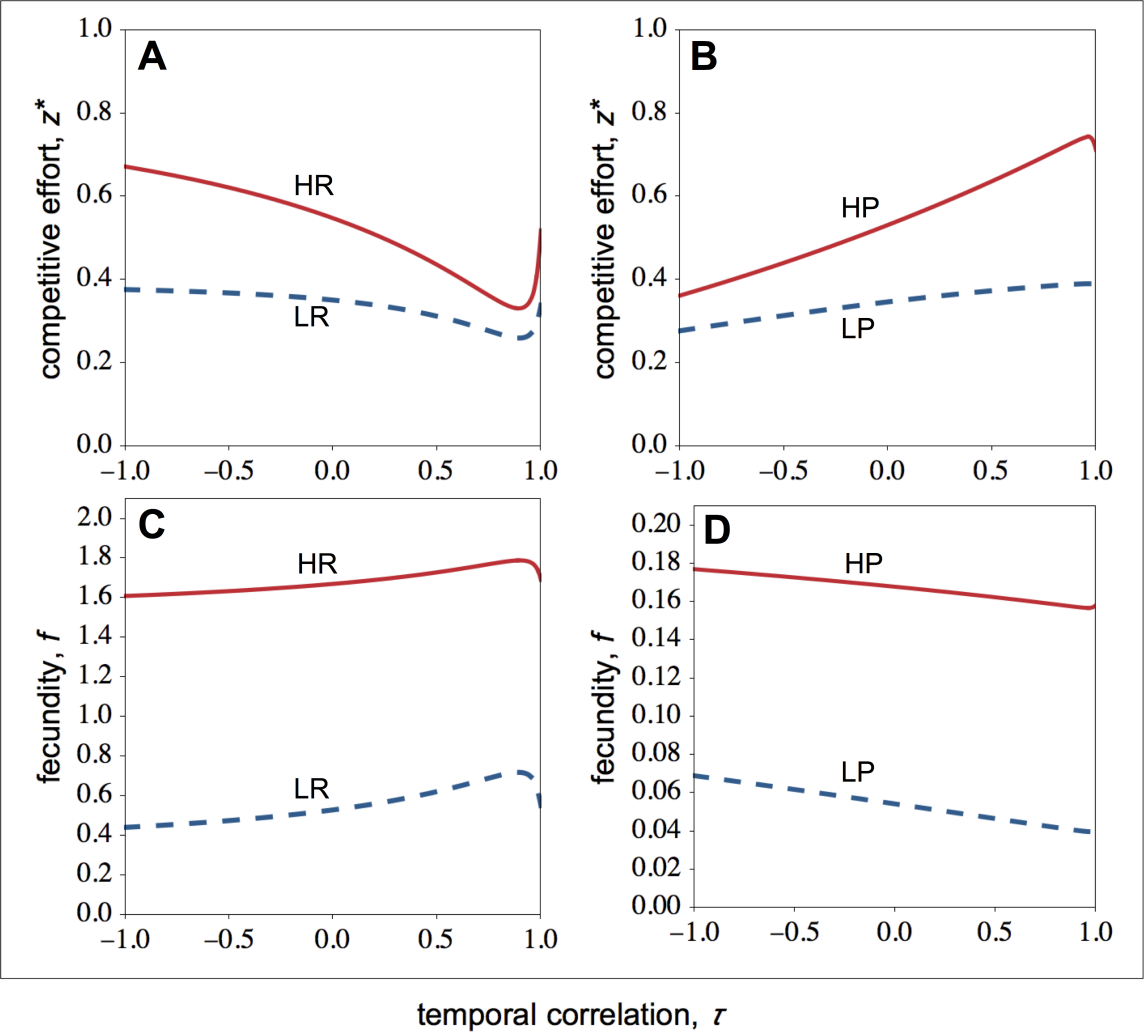
Optimal competitive effort and fecundity as a function of the temporal correlation. Optimal competitive effort (*z*^∗^) and fecundity (*f*) of high- (solid lines) and low-quality (dashed lines) individuals as a function of the temporal correlation (*τ*). (A) In resource-rich patches, temporal variation in resource availability increases investment in competitive effort. (B) In resource-poor patches, temporal variation in resource availability decreases investment in competitive effort. Temporal variation leads to lower fecundity in resource-rich patches (C), whilst it leads to higher fecundity in resource-poor patches (D). Parameter values: *c* = 0.9, *d* = 0.1, *p* = 0.5, *q*_*HR*_ = *q*_*HP*_ = 1.0, *q*_*LR*_ = *q*_*LP*_ = 0.1, *μ*_*R*_ = 1.0, *μ*_*P*_ = 0.1.

I now focus on the stable levels of competitive effort. In resource-rich patches, I find that as the environment becomes more stable, the competitive effort of high-quality individuals falls sharply, while the competitive effort of low-quality individuals falls more slowly (Fig. 2A). As a result, when the environment becomes more stable, the difference between the competitive effort of high- and low-quality individuals decreases. High-quality individuals invest less in competitive effort as the environment becomes more stable because environmental stability leads to relatively higher relatedness among social partners. Since in temporally stable environments, high- and low-quality individuals are more closely related (Fig. 1A), high-quality individuals are selected to share a bigger portion of the available resources with low-quality individuals.

Let us now look at the optimal competitive effort in resource-poor patches (Fig. 2B). Here, I find that as the environment becomes more stable, the competitive effort of high-quality individuals rises sharply, while the competitive effort of low-quality individuals rises more slowly (Fig. 2C). As a result, when the environment becomes more stable, the difference between the competitive effort of high- and the low-quality individuals rises. Owing to the steep increase in the competitive effort of high-quality individuals, both the fecundity of the high- and low-quality individuals falls (Fig. 2D).

In summary, environmental stability decreases the difference between the competitive effort of high- and low-quality individuals within resource-rich groups, while it reinforces the difference between the competitive effort of high- and low-quality individuals within resource-poor groups (compare Fig. 2A with Fig. 2B).

### Group productivity and inequality

**Figure 3:**
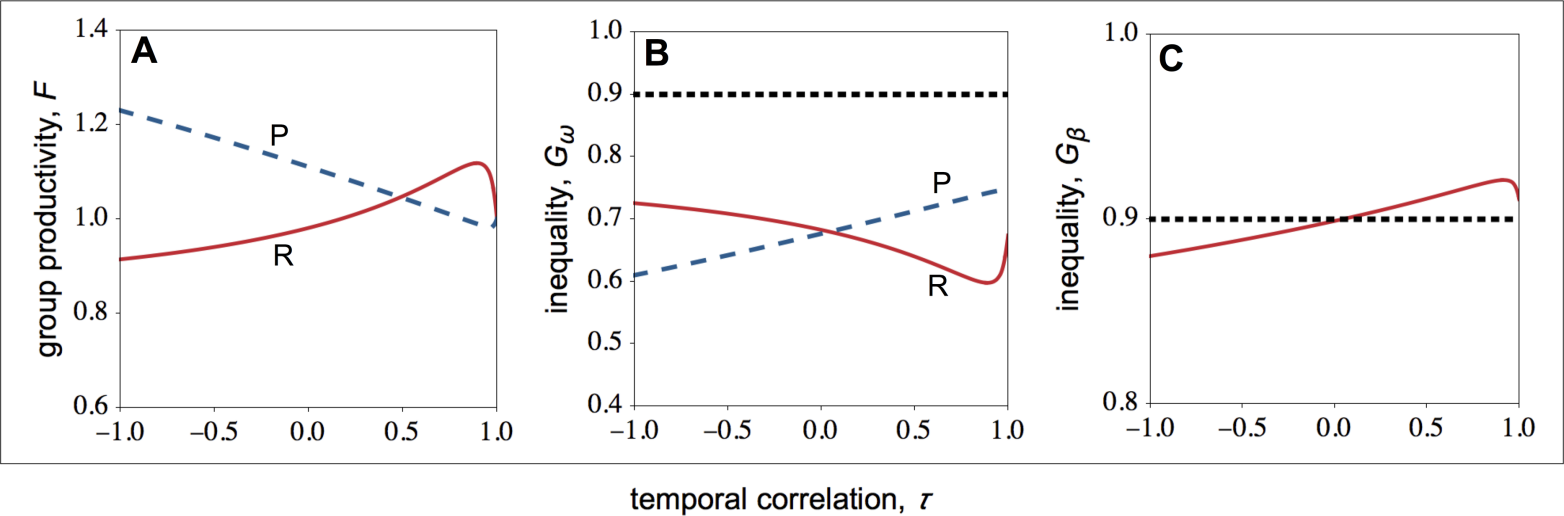
Group productivity, within-group inequality, and between-group inequality as a function of the temporal correlation. Group productivity (*F*), within-group inequality (*G*_*ω*_), and between-group inequality (*G*_*β*_) as a function of the temporal correlation (*τ*). Group productivity is measured relative to the productivity of a patch in stable environments (i.e., *τ* = 1) for each patch type (i.e., *F* = *F*(*τ*)∕*F*_0_, with *F*_0_ = *F*(*τ* = 1)). The dotted horizontal lines represent the initial levels of within-group inequality (B), and between-group inequality (C). (A) Temporal variation in resource availability leads to lower productivity in resource-rich patches, while it leads to higher productivity in resource-poor patches. (B) In resource-rich patches, inequality decreases as the environment becomes more stable. In resource-poor patches, inequality increases as the environment becomes more stable. (C) Between-group inequality rises as the environment becomes more stable. Parameter values: *c* = 0.9, *d* = 0.1, *p* = 0.5, *q*_*HR*_ = *q*_*HP*_ = 1.0, *q*_*LR*_ = *q*_*LP*_ = 0.1, *μ*_*R*_ = 1.0, *μ*_*P*_ = 0.1.

How do optimal competitive strategies influence individual, group productivity, and inequality? As shown in Fig. 2C, in resource-rich patches both the fecundity of high- and low-quality individuals rises as the environment becomes more stable. However, the fecundity of low-quality individuals rises more rapidly than the fecundity of high-quality individuals. As a result, within-group inequality among social partners falls as the environment becomes more stable in resource-rich patches (Fig. 3B). In resource-poor patches, both the fecundity of high- and low-quality individuals falls as the environment becomes more stable, as shown in Fig. 2D. However, the fecundity of low-quality individuals falls more rapidly than the fecundity of high-quality individuals. As a result, within-group inequality rises as the environment becomes more stable in resource-poor patches (Fig. 3B).

Overall, I find that both in resource-rich and resource-poor patches group productivity and within-group inequality are negatively correlated (compare Figs. 3A with 3B). Thus, while temporal stability leads to higher group productivity in resource-rich patches, in resource-poor patches it leads to lower group productivity (Fig. 3A). As a result, temporal stability leads to higher inequality between groups (Fig. 3C). In other words, in temporally stable environments, groups in resource-rich habitats become relatively more productive (i.e., the total number of offspring produced in each of the resource-rich habitats) than groups in resource-poor habitats as a result of their evolved social behaviours.

Finally, I find that social interactions always reduce the initial levels of within-group inequality, when I compare the initial level of inequality with the level of inequality at behavioural equilibrium (Fig. 3B). This is partially because social interactions have an additive effect on baseline fecundity. As a result, because the baseline fecundity of low-quality individuals is relatively smaller, an additive effect has a disproportional effect on the fecundity of low-quality individuals. By contrast, the change in the level of inequality between types of patches depends on the coefficient of temporal correlation (Fig. 3C). When the environment is relatively stable (*τ* > 0), between-group inequality rises relative to the initial levels of inequality. By contrast, when the environment is relatively unstable (*τ* < 0), between-group inequality falls relative to the initial levels of inequality.

## Extensions of the main model

So far I have considered that each patch has two breeders and that there is one high- and one low-quality breeder per patch. Here, I relax these two assumptions. First, I consider cases in which individuals acquire their quality early in life, and therefore patches can accommodate any combination of high- and low-quality individuals. Second, I consider scenarios in which patches can contain more than two individuals.

### Early-life acquisition of individual quality

My main model assumes that each patch accommodates exactly one high- and one low-quality individual. This mode of quality acquisition echoes scenarios in which social interactions mediate the state (or quality) of each group member, such as in the hierarchical structures observed in social insects or mammals. Alternatively, quality may be acquired early on during development, in which case patches need not have precisely one high-quality and one low-quality individual. Here, I modify my model to consider this scenario. In particular, I assume that offspring become high-quality with probability *Q* and low-quality with probability 1 – *Q*, and, for simplicity of analysis, I assume that *Q* = $ \frac{1}{2} $. Hence, the composition of each patch is determined by a random variable. More specifically, patches can be either pure patches and accommodate two high- or two low-quality individuals (i.e., HH or LL patches), or mixed patches and accommodate one high- and one low-quality individual (i.e., HL patches). The frequency of each patch type, denoted by *p*_*ij*_, is determined by the binomial distribution ${p}_{ij}= \left( {\scriptsize \begin{array}{@{}c@{}} \displaystyle n\\ \displaystyle k \end{array}} \right) {Q}^{k}{ \left( 1-Q \right) }^{n-k}$, where *k* is the number of high-quality individuals in the patch, *n* = 2 is the number of individuals per patch, and $ij\in \left\{ \mathrm{HH},\mathrm{HL},\mathrm{LL} \right\} $ (see Appendix S1 for more details).

**Figure 4:**
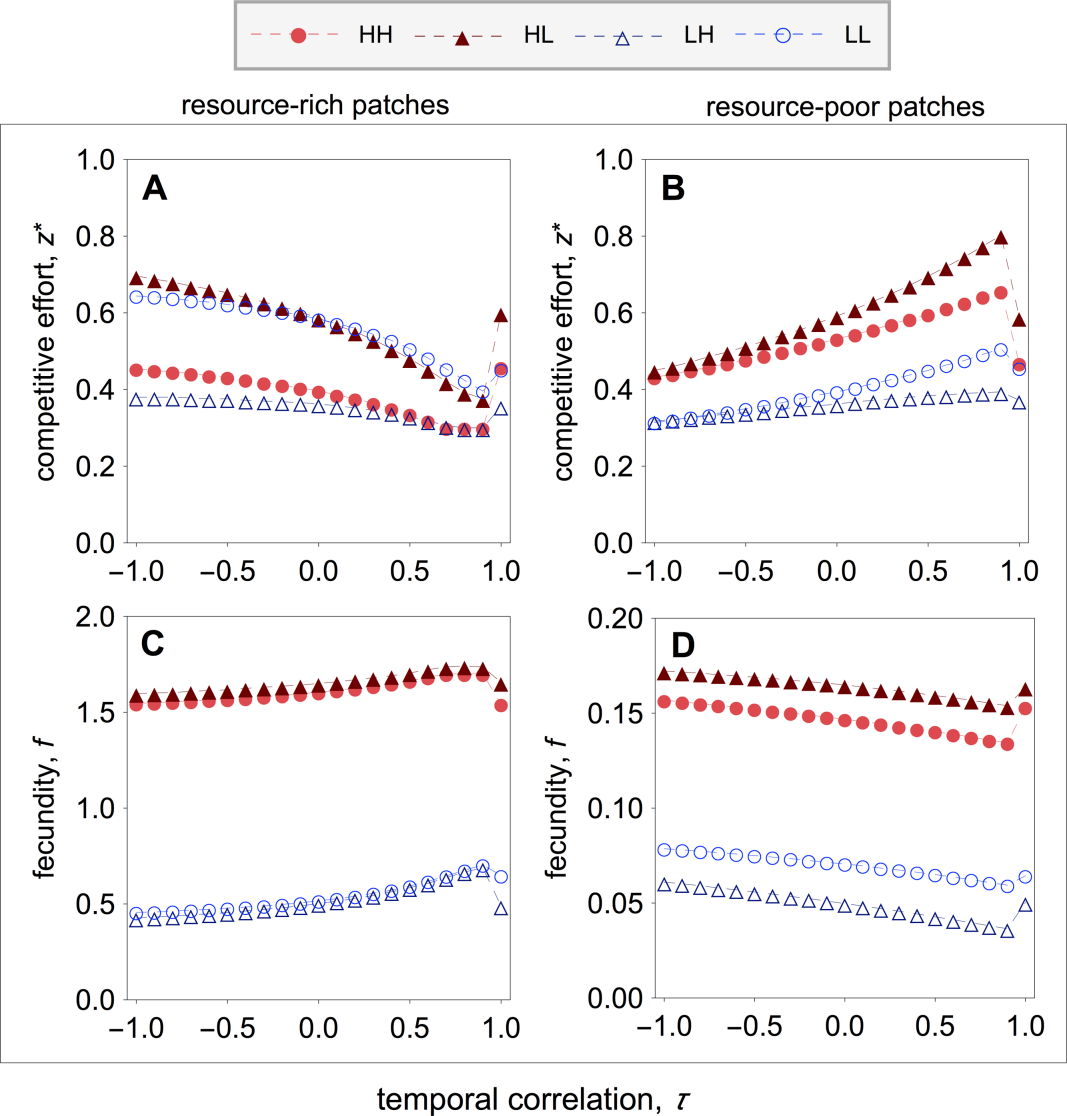
Optimal competitive effort and fecundity as a function of the temporal correlation. Optimal competitive effort (${z}_{\rho \varepsilon ,\sigma }^{\ast }$) and fecundity (*f*_*ρ*ε,*σ*_) of a quality-*ρ* individual in a type-*σ* patch, where the social partner is a quality-ε individual, as a function of the temporal correlation (*τ*). (A, B) In resource-rich patches, temporal variation in resource availability increases investment in competitive effort. (C, D) In resource-poor patches, temporal variation in resource availability decreases investment in competitive effort. Temporal variation leads to lower fecundity in resource-rich patches, whilst it leads to higher fecundity in resource-poor patches. Parameter values: *c* = 0.9, *d* = 0.1, *p* = 0.5, *q*_*HR*_ = *q*_*HP*_ = 1.0, *q*_*LR*_ = *q*_*LP*_ = 0.1, *μ*_*R*_ = 1.0, *μ*_*P*_ = 0.1.

I find that early-life acquisition of quality leads to patterns of investment in competitive effort that are similar to those of the main model (compare Figs. 2 with 4). More specially, I find that in resource-rich patches competitive effort is lowest when the environment is relatively stable, and rises as the environment becomes unstable (Fig. 4A). Within resource-rich patches, however, there are noticeable differences in the average levels of competitive effort that strongly depend on patch composition. In particular, the highest and lowest levels of competitive effort are observed in mixed patches, where high-quality individuals express the highest levels of competitive effort and low-quality individuals express the lowest levels of competitive effort (Fig. 4A). Within pure patches, competitive effort is highest among low-quality breeders and lowest among high-quality breeders (Fig. 4A). Turning the attention to resource-poor patches, I find that competitive effort is highest when the environment is relatively stable, and falls as the environment becomes unstable (Fig. 4B). As in resource-rich patches, the highest and lowest levels of competitive effort are observed in mixed patches, where high-quality individuals express the highest levels of competitive effort, and low-quality breeders express the lowest levels of competitive effort (Fig. 4B). Within pure patches, competitive effort is highest among high-quality breeders and lowest among low-quality breeders (Fig. 4B).

**Figure 5:**
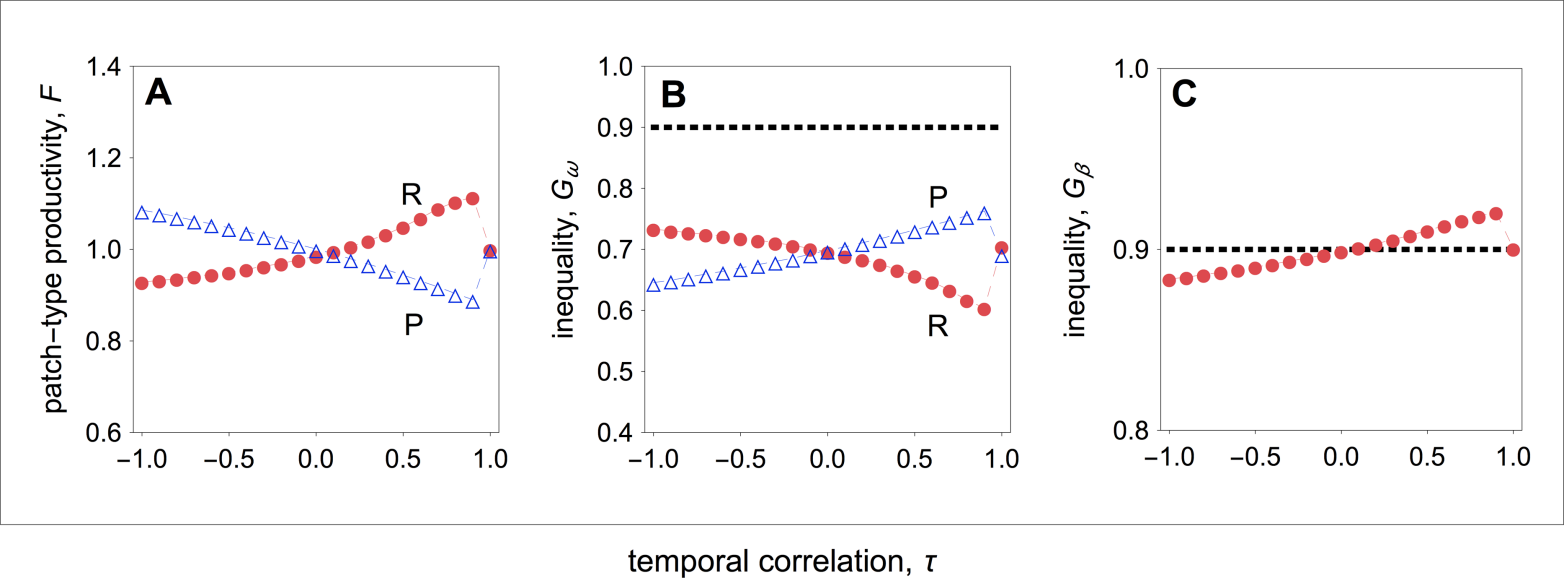
Group productivity, within-group inequality, and between-group inequality as a function of the temporal correlation. Group productivity (*F*_*σ*_), within-group inequality (*G*_*σ*,*ω*_), and between-group inequality (*G*_*β*_) as a function of the temporal correlation (*τ*). Group productivity is measured relative to the productivity of patches in stable environments (i.e., *τ* = 1) for each patch type (i.e., *F*_*σ*_ = *F*_*σ*_(*τ*)∕*F*_0,*σ*_, with *F*_0,*σ*_ = *F*_*σ*_(*τ* = 1)). The dotted horizontal lines represent the initial levels of within-group inequality (B), and between-group inequality (C). (A) Temporal variation in resource availability leads to lower productivity in resource-rich patches, while it leads to higher productivity in resource-poor patches. (B) In resource-rich patches, inequality is lowest when the environment is relatively stable. In resource-poor patches, inequality is highest when the environment is relatively stable. (C) Between-group inequality is lowest in fluctuating environment and highest when the environment is relatively stable. Parameter values: *c* = 0.9, *d* = 0.1, *p* = 0.5, *q*_*HR*_ = *q*_*HP*_ = 1.0, *q*_*LR*_ = *q*_*LP*_ = 0.1, *μ*_*R*_ = 1.0, *μ*_*P*_ = 0.1.

These patterns of competitive effort lead to patterns of group productivity and inequality as a function of temporal correlation that are similar to those found in the main model (compare Figs. 3 with 5). In resource-rich patches, productivity is highest when the environment is relatively stable, and decreases as environmental instability increases (Fig. 5A). In resource-poor patches, productivity is lowest when the environment is relatively stable, and increases as environmental instability increases (Fig. 5A). Both in resource-rich and resource-poor patches, inequality is inversely correlated with patch productivity (Figs. 5A and 5B). In resource-rich patches, inequality is lowest when the environment is relatively stable, and it increases as environmental instability increases (Fig. 5B). In resource-poor patches, inequality is highest when the environment is relatively stable, and decreases as environmental instability increases (Fig. 5B). As a result, I find that inequality between patch types is highest when the environment is relatively stable, and decreases as environmental instability increases (Fig. 5C).

### The effect of patch size

My main model assumes that each patch accommodates exactly two breeders. More generally, group size can be much larger than two, a factor that affects the genetic structure of the population, and therefore has implications for the evolution of social behaviour. Here, I contemplate this scenario by assuming that group size can be greater than two. As in the main model, I assume that half of the group members are high-quality and half are low-quality. I find that as patch size increases the relatedness between group mates decreases, which leads to higher levels of competitive effort. As a result, the amount of public good available to group members decreases, and therefore inequality increases (see Figure I1 in the Appendix S1). Despite this, I find that the patterns of competitive effort as a function of the coefficient of temporal correlation remain similar to those obtained in the main model.

## Discussion

The expression of social behaviour among group members and the level of inequality within societies varies widely between species, ranging from egalitarian societies (Cashdan, 1980; Cant, 2000; Packer, Pusey & Eberly, 2001) to extremely unequal societies (Jarvis, 1981;  Boone, 1986; Rood, 1986; Boehm, 1999; Clutton-Brock et al., 2001; Charpentier et al., 2005). In the present study, I have investigated how spatial and temporal variation in resource availability shapes the evolution of social behaviour within groups, and how this, in turn, influences the levels of inequality both within and between groups. I found that within each group, high-quality mothers produce more offspring and therefore experience higher levels of kin competition. Thus, to reduce the degree of competition for local resources experienced by their own offspring, they are selected to suppress the reproduction of their poorer counterparts. Conversely, low-quality mothers produce fewer offspring and therefore experience lower levels of kin competition. As a result, they optimise their inclusive fitness by directing their helping effort towards higher-status individuals. I showed that spatial and temporal variation in resource availability between societies has a considerable impact on these selective pressures, and therefore it is a significant factor mediating the transfers of resources that take place within societies.

My analysis predicts that in temporally stable environments, wealthier societies show a lower degree of inequality. By contrast, poorer societies show a higher degree of inequality. This pattern is reversed when the environment becomes unstable, in which case inequality rises within wealthier societies but falls within poorer societies. In wealthier societies, temporal stability causes lower inequality and higher group productivity. In poorer societies, by contrast, temporal stability causes greater inequality but lower group productivity. Thus, my model predicts a negative association between inequality and the productivity of a society. Because inequality is correlated with lower productivity, this leads to rising inequality between societies when the environment becomes more stable, with wealthier societies becoming relatively more productive than poorer societies. Temporal stability impacts competitive effort in two separate ways. First, temporal stability increases relatedness in resource-rich patches, which leads to the expression of lower levels of competitive effort, which in turn benefits group productivity. In addition, higher relatedness has a disproportionate impact on the competitive effort of high-quality individuals, which results in slightly lower levels of inequality. These findings highlight the role of relatedness as a major factor in the evolution of cooperation and inequality.

My model shows that in resource-rich patches selection on helping peaks when the environment is relatively stable and gradually decreases as the environment becomes more unstable. In resource-poor patches, selection on helping is lowest when the environment is relatively stable and gradually increases as the environment becomes more unstable. These findings parallel those obtained by Rodrigues & Gardner (2012), who found a similar pattern of selection on helping as a function of temporal stability. Rodrigues & Gardner (2012), however, have not considered variation in individual quality within patches. My model shows that the patterns of helping as a function of temporal stability depend strongly on the quality of individuals within each patch.

I have shown that low-quality individuals invest more into helping than high-quality individuals, irrespective of patch quality. This is in line with the results of Rodrigues & Gardner (2013a), who have reached a similar conclusion. However, these predictions contrast with the results of previous work (e.g., Frank, 1996; Frank, 2010). For instance, in the context of policing (i.e., suppression of competition), Frank (1996) has shown that more vigorous individuals invest disproportionally more into policing. Because investment into policing is costly to the actor, but beneficial to the group, policing acts as an equalising force within the society, such that at equilibrium all individuals exhibit the same fitness. As in Frank’s model, the social interactions in my model also have an equalising effect within societies, with the level of inequality at equilibrium being less than their initial values. However, I never observe zero within-group inequality at equilibrium, and inequality always remains relatively high. This may be because of the particular behavioural functions that have been chosen to model the fecundity of each individual. In my model, I have considered that social interactions have an additive effect on baseline fecundity, and therefore I have controlled for ‘vigour’ by disentangling costs and benefits from the ‘baseline’ quality of individuals. In Frank’s model there is an association between quality and costs and benefits, an effect that ultimately drives the level of inequality within the group.

In general, my model generates a set of reaction norms that describe multiple key variables, such as relatedness, kin competition, social behaviour, inequality, and group productivity, as a function of resource availability. This comprehensive set of results can be used to explain and understand observational and experimental data in different species. For example, in the banded mongoose *Mungos mungo*, high resource availability is associated with less inequality (Nichols et al., 2012). When resource availability is high, dominant females do not suppress the reproduction of subordinate females. When resources are scarce, by contrast, dominant females suppress the breeding attempts of subordinate females. Thus, higher resource availability is associated with lower aggressive behaviour by dominant individuals, and higher competitiveness by subordinate individuals. This cluster of associations between different variables is in agreement with the model results if we assume a temporally stable environment.

There are, however, alternative explanations for these observations. For instance, we may suppose that the fecundity of dominant mothers is limited by their physiology and not by resource availability, as I assumed in my model. In meerkats, for instance, dominant females are more likely to suppress the reproduction of subordinate females when pregnant (Clutton-Brock et al., 2010). Under such scenario, an increase in resource availability does not necessarily increase the intensity of kin competition for dominant females, as their capacity to produce offspring may not scale proportionally with the abundance of resources. I would then expect that natural selection would act against dominant females who tend to suppress the reproductive output of subordinate females. This effect would further reinforce the findings of my model. More generally, this is an intriguing hypothesis that should be explored further.

The biology of the social amoeba *Dictyostelium discoideum* provides additional insights regarding the significance of my results. In this social amoeba, higher resource availability is associated with lower inequality (Castillo, Queller & Strassmann, 2011). In this case, however, lower inequality may be mediated by a change in the composition of the group, rather than a decrease in the intensity of kin competition. Higher resource-availability leads to a change in the fraction of high-quality cells, and high-quality cells have a greater tendency to become reproductive cells rather than altruistic spore cells. In my model, by contrast, the proportion of high- and low-quality individuals remains constant, irrespective of the amount of available resources. Including changes in group composition with the availability of resources remains an important question and should be the focus of more studies.

My results predict high variation in social behaviour and inequality between different groups in the same populations. Plasticity in social and reproductive behaviour has been observed in intermediate-scale human societies. My predictions are consistent with the data from the human demographic transition, in which intermediate-scale societies already exhibit some degree of inequality among group members (e.g., Prentiss et al., 2007). The model also predicts an association been resource availability and the coefficient of relatedness within each society, together with an increase in the selfish tendencies of dominant individuals, an increase in submissive behaviours by subordinate individuals, and an increase in competition for local resources. It is very likely that these correlations were also present in intermediate-scale human societies, thus, the selective pressures outlined in my model may have contributed to the patterns of inequality in these societies. Notwithstanding, it is very likely that other forces have played a significant role in the patterns of inequality in human societies. In particular, increasing costs of dispersal, together with transfers of wealth between generations may have exacerbated inequality within societies. Moreover, the size of groups, the number of social classes, and the distribution of resources among the different classes can vary significantly in human societies. All these factors are likely to influence the social behaviour of group members, and consequently the evolution of inequality. Extending my model in these different directions can provide exciting opportunities for future research.

My analysis provides a framework to understand how spatial and temporal variation in resource availability mediate the evolution of wealth transfers among group members in an explicit demographic setting. Given the generality of the approach, my model applies to a wide range of species, including humans. Future work might focus on particular species and study the influence of species-specific factors on social behaviour and wealth transfers among group members. For instance, one might consider a model with overlapping generations in which age mediates the reproductive value and dominance-rank of individuals (e.g., Rodrigues, 2018). In addition, one might also consider a model where group size is mediated by resource availability, and where the dispersal rates of group members co-evolve with cooperation (e.g., Rodrigues & Gardner, 2016). Furthermore, future studies could explore how the history of resource availability in each patch influences the expression of social behaviour. Lastly, one might consider cases where groups can become extinct, and new groups can be founded either by immigrants of different origins or by groups composed of family members.

##  Supplemental Information

10.7717/peerj.5488/supp-1 Appendix S1 Appendix

10.7717/peerj.5488/supp-2 Supplemental Information 1 Mathematica code

## Additional Information and Declarations

### Data Availability

The following information was supplied regarding data availability: The Supplemental File includes the code used to implement the model.

## References

[ref-1] Alizon S, Taylor P (2008). Empty sites can promote altruistic behavior. Evolution.

[ref-2] Bao M, Wild G (2012). Reproductive skew can provide a net advantage in both conditional and unconditional social interactions. Theoretical Population Biology.

[ref-3] Boehm C (1999). Hierarchy in the forest: the evolution of egalitarian behaviour.

[ref-4] Boomsma JJ (2009). Lifetime monogamy and the evolution of eusociality. Philosophical Transactions of the Royal Society of London, Series B.

[ref-5] Boone JL (1986). Parental investment and elite family structure in preindustrial states: a case study of late medieval-early modern Portuguese genealogies. American Anthropologist.

[ref-6] Bourke AFG (2011). Principles of social evolution.

[ref-7] Callaway RM, Brooker RW, Choler P, Kikvidze Z, Lortie CJ, Michalet R, Paolini L, Pugnaire FI, Newingham B, Aschehoug ET, Armas C, Kikodze D, Cook BJ (2002). Positive interactions among alpine plants increase with stress. Nature.

[ref-8] Cant MA (2000). Social control of reproduction in banded mongooses. Animal Behavior.

[ref-9] Cashdan EA (1980). Egalitarianism among hunters and gatherers. American Anthropologist.

[ref-10] Castillo DI, Queller DC, Strassmann JE (2011). Cell condition, competition, and chimerism in the social amoeba Dictyostelium discoideum. Ethology Ecology & Evolution.

[ref-11] Charnov EL (1977). An elementary treatment of the genetical theory of kin-selection. Journal of Theoretical Biology.

[ref-12] Charpentier M, Peignot P, Hossaert-McKey M, Gimenez O, Setchell JM, Wickings EJ (2005). Constraints on control: factors influencing reproductive success in male mandrills (*Mandrillus sphinx*). Behavioral Ecology.

[ref-13] Christiansen FB (1991). On conditions for evolutionary stability for a continuously varying character. The American Naturalist.

[ref-14] Clutton-Brock T, Brotherton PNM, Russell AF, O’Riain MJ, Gaynor D, Kansky R, Griffin A, Manser M, Sharpe L, McIlrath GM, Small T, Moss A, Monfort S (2001). Cooperation, control, and concession in meerkat groups. Science.

[ref-15] Clutton-Brock TH, Hodge SJ, Flower TP, Spong GF, Young AJ (2010). Adaptive suppression of subordinate reproduction in cooperative mammals. The American Naturalist.

[ref-16] Cornwallis CK, West SA, Davis KE, Griffin AS (2010). Promiscuity and the evolutionary transition to complex societies. Nature.

[ref-17] Cote J, Fogarty S, Tymen B, Sih A, Brodin T (2013). Personality-dependent dispersal cancelled under predation risk. Proceedings of the Royal Society Biological Sciences Series B.

[ref-18] Covas R, Du Plessis MA, Doutrelant C (2008). Helpers in colonial cooperatively breeding sociable weavers Philetairus socius contribute to buffer the effects of adverse breeding conditions. Behavioral Ecology and Sociobiology.

[ref-19] Craig R (1983). Subfertility and the evolution of eusociality by kin selection. Journal of Theoretical Biology.

[ref-20] Davies NB, Krebs JR, West SA (2012). An introduction to behavioural ecology.

[ref-21] Débarre F, Hauert C, Doebeli M (2014). Social evolution in structured populations. Nature Communications.

[ref-22] Ellis L (1995). Dominance and reproductive success among nonhuman animals: a cross-species comparison. Ethology and Sociobiology.

[ref-23] Emlen ST (1982). The evolution of helping. I. An ecological constraints model. The American Naturalist.

[ref-24] Eshel I (1996). On the changing concept of evolutionary population stability as a reflection of a changing point of view in the quantitative theory of evolution. Journal of Mathematical Biology.

[ref-25] Fisher RA (1930). The genetical theory of natural selection.

[ref-26] Flannery K, Marcus J (2012). The creation of inequality: how our prehistoric ancestors set the stage for monarchy, slavery, and empire.

[ref-27] Frank SA (1994). Kin selection and virulence in the evolution of protocells and parasites. Proceedings of the Royal Society Biological Sciences Series B.

[ref-28] Frank SA (1996). Policing and group cohesion when resources vary. Animal Behavior.

[ref-29] Frank SA (1998). Foundations of social evolution.

[ref-30] Frank SA (2010). A general model of the public goods dilemma. Journal of Evolutionary Biology.

[ref-31] Gardner A, West SA (2006). Demography, altruism, and the benefits of budding. Journal of Evolutionary Biology.

[ref-32] Grafen A (2006). A theory of Fisher’s reproductive value. Journal of Mathematical Biology.

[ref-33] Guinote A, Cotzia I, Sandhu S, Siwa P (2015). Social status modulates prosocial behavior and egalitarianism in preschool children and adults. Proceedings of the National Academy of Sciences of the United States of America.

[ref-34] Hamilton WD (1964). The genetical evolution of social behaviour. I & II. Journal of Theoretical Biology.

[ref-35] Hughes WOH, Oldroyd BP, Beekman M, Ratnieks FLW (2008). Ancestral monogamy shows kin selection is key to the evolution of eusociality. Science.

[ref-36] Jarvis JU (1981). Eusociality in a mammal—cooperative breeding in naked mole-rat colonies. Science.

[ref-37] Jetz W, Rubenstein DR (2011). Environmental uncertainty and the global biogeography of cooperative breeding in birds. Current Biology.

[ref-38] Krams I, Bērziņs A, Krama T, Wheatcroft D, IgAune K, Rantala MJ (2010). The increased risk of predation enhances cooperation. Proceedings of the Royal Society Biological Sciences Series B.

[ref-39] Kropotkin P (1902). Mutual aid.

[ref-40] Lehmann L, Rousset F (2010). How life history and demography promote or inhibit the evolution of helping behaviours. Philosophical Transactions of the Royal Society of London. Series B.

[ref-41] Lion S, Gandon S (2009). Habitat saturation and the spatial evolutionary ecology of altruism. Journal of Evolutionary Biology.

[ref-42] Lukas D, Clutton-Brock T (2012). Cooperative breeding and monogamy in mammalian societies. Proceedings of the Royal Society Biological Sciences Series B.

[ref-43] Markiewicz DA, O’Donnell S (2001). Social dominance, task performance and nutrition: implications for reproduction in eusocial wasps. Journal of Comparative Physiology. A, Sensory, Neural, and Behavioral Physiology.

[ref-44] Nichols HJ, Bell M, Hodge SJ, Cant MA (2012). Resource limitation moderates the adaptive suppression of subordinate breeding in a cooperatively breeding mongoose. Behavioral Ecology.

[ref-45] Packer C, Pusey AE, Eberly LE (2001). Egalitarianism in female African lions. Science.

[ref-46] Peña J, Nöldeke G, Lehmann L (2015). Evolutionary dynamics of collective action in spatially structured populations. Journal of Theoretical Biology.

[ref-47] Pettay JE, Lahdenperä M, Rotkirch A, Lummaa V (2016). Costly reproductive competition between co-resident females in humans. Behavioral Ecology.

[ref-48] Powers ST, Lehmann L (2013). The co-evolution of social institutions, demography, and large-scale human cooperation. Ecology Letters.

[ref-49] Prentiss AM, Lyons N, Harris LE, Burns MRP, Godin TM (2007). The emergence of status inequality in intermediate scale societies: a demographic and socio-economic history of the Keatley Creek site, British Columbia. Journal of Anthropological Archaeology.

[ref-50] Przepiorka W, Diekmann A (2013). Individual heterogeneity and costly punishment: a volunteer’s dilemma. Proceedings of the Royal Society Biological Sciences Series B.

[ref-51] Rodrigues AMM (2018). Demography, life history and the evolution of age-dependent social behaviour. Journal of Evolutionary Biology.

[ref-52] Rodrigues AMM, Gardner A (2012). Evolution of helping and harming in heterogeneous populations. Evolution.

[ref-53] Rodrigues AMM, Gardner A (2013a). Evolution of helping and harming in heterogeneous groups. Evolution.

[ref-54] Rodrigues AMM, Gardner A (2013b). Evolution of helping and harming in viscous populations when group size varies. The American Naturalist.

[ref-55] Rodrigues AMM, Gardner A (2016). The constant philopater hypothesis: a new life history invariant for dispersal evolution. Journal of Evolutionary Biology.

[ref-56] Rodrigues AMM, Kokko H (2016). Models of social evolution: can we do better to predict who helps whom to achieve what?. Philosophical Transactions of the Royal Society of London. Series B.

[ref-57] Rood JP, Rubenstein DI, Wrangham RW (1986). Ecology and social evolution of the dwarf mongoose. Ecological aspects of social evolution.

[ref-58] Rousset F (2004). Genetic structure and selection in subdivided populations.

[ref-59] Rousset F, Ronce O (2004). Inclusive fitness for traits affecting metapopulation demography. Theoretical Population Biology.

[ref-60] Scheidel W (2017). The great leveler: violence and the history of inequality from the Stone Age to the twenty-first century.

[ref-61] Stacey PB, Ligon JD (1991). The benefits-of-philopatry hypothesis for the evolution of cooperative breeding: variation in trerritory quality and group size effects. The American Naturalist.

[ref-62] Surbeck M, Langergraber KE, Fruth B, Vigilant L, Hohmann G (2017). Male reproductive skew is higher in bonobos than chimpanzees. Current Biology.

[ref-63] Taylor PD (1990). Allele-frequency change in a class-structured population. American Naturalist.

[ref-64] Taylor PD (1992). Altruism in viscous populations—an inclusive fitness model. Evolutionary Ecology.

[ref-65] Taylor PD (1996). Inclusive fitness arguments in genetic models of behaviour. Journal of Mathematical Biology.

[ref-66] Taylor PD, Frank SA (1996). How to make a kin selection model. Journal of Theoretical Biology.

[ref-67] Uematsu K, Kutsukake M, Fukatsu T, Shimada M, Shibao H (2010). Altruistic colony defense by menopausal female insects. Current Biology.

[ref-68] Vehrencamp SL (1983). Optimal degree of skew in cooperative societies. American Zoologist.

[ref-69] Weesie J (1993). Asymmetry and timing in the volunteer’s dilemma. Journal of Conflict Resolution.

[ref-70] West SA, Griffin AS, Gardner A (2007). Evolutionary explanations for cooperation. Current Biology.

[ref-71] West SA, Murray MG, Machado CA, Griffin AS, Herre EA (2001). Testing Hamilton’s rule with competition between relatives. Nature.

[ref-72] West SA, Pen I, Griffin AS (2002). Cooperation and competition between relatives. Science.

[ref-73] West-Eberhard MJ, Alexander RD, Tinkle DW (1981). Intragroup selection and the evolution of insect societies. Natural selection and social behavior.

[ref-74] Whitlock MC, Davis BH, Yeaman S (2007). The costs and benefits of resource sharing: reciprocity requires resource heterogeneity. Journal of Evolutionary Biology.

[ref-75] Wright S (1931). Evolution in mendelian populations. Genetics.

