# Supplementary material for: Resource availability and adjustment of social behaviour influence patterns of inequality and productivity across societies
Source: PeerJ. 2018 Oct 2;6:e5488. doi: 10.7717/peerj.5488 (PMC6173167; doi:10.7717/peerj.5488)
Supplement: Appendix S1 [file peerj-06-5488-s001.pdf]

Resource availability and adjustment of social behaviour influence patterns of inequality and productivity across societies

Supplementary material

Antonio M. M. Rodrigues[1,2,*]

1. Department of Zoology, University of Cambridge, Downing Street, CB2 3EJ Cambridge, United Kingdom.
2. Wolfson College, Barton Road, Cambridge, CB3 9BB United Kingdom.

[*] corresponding author, email: ammr3@cam.ac.uk

Contents:

Appendix A - Ecology
Appendix B - Reproductive success
Appendix C - Marginal fitness
Appendix D - Stable-class frequencies and reproductive value
Appendix E - Relatedness
Appendix F - Optimal behaviour and convergence stability
Appendix G - Fecundity and social interactions
Appendix H - Early-life acquisition of individual quality
Appendix I - Patch size
References

Appendix A. Ecology

Given the life-cycle outline in the main text, the ecological dynamics can be described by a transition matrix (Rodrigues and Gardner 2012) that is given by

$$\mathbf{E} = \begin{pmatrix} p_{\mathrm{R}\to\mathrm{R}} & p_{\mathrm{P}\to\mathrm{R}} \\ p_{\mathrm{R}\to\mathrm{P}} & p_{\mathrm{P}\to\mathrm{P}} \end{pmatrix}. \tag{A1}$$

The right-eigenvector of matrix **E** gives the fraction of each patch type at ecological equilibrium, which is given by

$$p_{\mathrm{R}} = \frac{1-p_{\mathrm{P}\to\mathrm{P}}}{2-(p_{\mathrm{R}\to\mathrm{R}}+p_{\mathrm{P}\to\mathrm{P}})}, \text{ and} \tag{A2}$$

$$p_{\mathrm{P}} = 1 - p_{\mathrm{R}} = \frac{1-p_{\mathrm{R}\to\mathrm{R}}}{2-(p_{\mathrm{R}\to\mathrm{R}}+p_{\mathrm{P}\to\mathrm{P}})}, \tag{A3}$$

for resource-rich and resource-poor patches, respectively. If we define a random variable T_{t}, denoting the state of a focal patch in season t, then the coefficient of correlation between two successive seasons, denoted by τ, is defined as $\tau \equiv \mathrm{cov}(T_{\mathrm{t}},T_{\mathrm{t+1}})\sqrt{}(\mathrm{var}(T_{\mathrm{t}})\mathrm{var}(T_{\mathrm{t+1}}))$. Expanding the right-hand side of this equation, the coefficient of correlation becomes

$$\tau = p_{\mathrm{R}\to\mathrm{R}} - (1 - p_{\mathrm{P}\to\mathrm{P}}), \tag{A4}$$

where $-1 \leq \tau \leq 1$ (Rodrigues and Gardner 2012).

Appendix B. Reproductive success

Here I define the expressions for the class-specific reproductive success of breeders, i.e. $w_{\rho\pi\rightarrow\eta\gamma}$. Let us denote the probability that a patch in state π becomes a patch in state γ in the next generation by $p_{\pi\rightarrow\gamma}$. The reproductive success of a mother in condition ρ in a patch of quality π through her offspring that become breeders in condition η in patches of quality γ is then given by

$$w_{\rho\pi\rightarrow\eta\gamma} = f_{\rho\pi}(x_{\mathrm{H}\pi}, x_{\mathrm{L}\pi})\Big(w_{\pi}^{\phi}(x_{\mathrm{H}\pi}, x_{\mathrm{L}\pi})p_{\pi\rightarrow\gamma} + \Big(\sum_{\gamma'\in\{\mathrm{R,P}\}} p_{\gamma'}p_{\gamma'\rightarrow\gamma}w_{\gamma'}^{\delta}(z_{\mathrm{H}\gamma'}, z_{\mathrm{L}\gamma'})\Big)\Big) \quad, \pi \in \{\mathrm{R,P}\}, \tag{B1}$$

where

$$w_{\pi}^{\phi}(x_{\mathrm{H}\pi}, x_{\mathrm{L}\pi}) = \frac{1-d}{\left(\sum_{\rho\in\{\mathrm{H,L}\}} f_{\rho\pi}(x_{\mathrm{H}\pi}, x_{\mathrm{L}\pi})\right)(1-d) + \left(\sum_{\gamma\in\{\mathrm{R,P}\}} p_{\gamma} \sum_{\rho\in\{\mathrm{H,L}\}} f_{\rho\gamma}(z_{\mathrm{H}\gamma}, z_{\mathrm{L}\gamma})\right)d(1-c)}, \tag{B2}$$

is the reproductive success of a single philopatric offspring competing for a breeding site in a type-π patch, and

$$w_{\gamma'}^{\delta}(z_{\mathrm{H}\gamma'}, z_{\mathrm{L}\gamma'}) = \frac{d(1-c)}{\left(\sum_{\rho\in\{\mathrm{H,L}\}} f_{\rho\gamma'}(z_{\mathrm{H}\gamma'}, z_{\mathrm{L}\gamma'})\right)(1-d) + \left(\sum_{\gamma\in\{\mathrm{R,P}\}} p_{\gamma} \sum_{\rho\in\{\mathrm{H,L}\}} f_{\rho\gamma}(z_{\mathrm{H}\gamma}, z_{\mathrm{L}\gamma})\right)d(1-c)}, \tag{B3}$$

is the reproductive success of a single dispersed offspring competing for a breeding site in a type-γ' patch.

Appendix C. Marginal fitness

As we have seen in the main text, the average fitness of a random recipient is given by

$$\frac{\mathrm{d}\bar{w}}{\mathrm{d}g_{\alpha\sigma}} = \sum_{\pi\in\{\mathrm{R,P}\}} \sum_{\rho\in\{\mathrm{H,L}\}} u_{\rho\pi} v_{\rho\pi} \frac{\mathrm{d}W_{\rho\pi}}{\mathrm{d}g_{\alpha\sigma}}. \quad \text{(C1)}$$

From equation (3) in the main text, expanding the right-hand side of equation (C1), and because social interactions occur within the local patch only, we get

$$\frac{\mathrm{d}\bar{w}}{\mathrm{d}g_{\alpha\sigma}} = \sum_{\rho\in\{\mathrm{H,L}\}} u_{\rho\pi} \left(\sum_{\gamma\in\{\mathrm{R,P}\}} \sum_{\eta\in\{\mathrm{H,L}\}} \sum_{\lambda\in\{\mathrm{H,L}\}} \frac{\partial w_{\rho\varphi\to\eta\gamma}}{\partial x_{\lambda\sigma}} \frac{\mathrm{d}x_{\lambda\sigma}}{\mathrm{d}g_{\lambda\sigma}} \frac{\mathrm{d}g_{\lambda\sigma}}{\mathrm{d}g_{\alpha\sigma}} v_{\eta\gamma} \right), \quad \text{(C2)}$$

where: the partial derivatives represent the effect of the phenotype on the class-specific reproductive success; the derivatives of the phenotype with respect to the breeding value, denoted by g, represent the phenotype-to-genotype mapping, which, without loss of generality can be set to one; and the derivatives of breeding values with respect to breeding values represent the coefficient of consanguinity, $g_{\alpha\lambda,\sigma}$, between the actor α and the recipient λ (Bulmer 1994; Rodrigues and Gardner 2016). If we expand the right-hand side of equation (C2) and if we normalise it with respect to the coefficient of consanguinity between the actor and herself, we obtain Hamilton's rule given by equation (6) in the main text.

Appendix D. Stable-class frequencies and reproductive value

From the expressions for the reproductive success of breeding females, as derived above in section B, we define a matrix of expected fitness, which is given by

$$\mathbf{w} = \begin{pmatrix} w_{\mathrm{HR}\to\mathrm{HR}} & w_{\mathrm{LR}\to\mathrm{HR}} & w_{\mathrm{HP}\to\mathrm{HR}} & w_{\mathrm{LP}\to\mathrm{HR}} \\ w_{\mathrm{HR}\to\mathrm{LR}} & w_{\mathrm{LR}\to\mathrm{LR}} & w_{\mathrm{HP}\to\mathrm{LR}} & w_{\mathrm{LP}\to\mathrm{LR}} \\ w_{\mathrm{HR}\to\mathrm{HP}} & w_{\mathrm{LR}\to\mathrm{HP}} & w_{\mathrm{HP}\to\mathrm{HP}} & w_{\mathrm{LP}\to\mathrm{HP}} \\ w_{\mathrm{HR}\to\mathrm{LP}} & w_{\mathrm{LR}\to\mathrm{LP}} & w_{\mathrm{HP}\to\mathrm{LP}} & w_{\mathrm{LP}\to\mathrm{LP}} \end{pmatrix}. \quad \text{(D1)}$$

The stable-class frequencies (u) are given by the elements of the right-eigenvector corresponding to the leading eigenvector of this matrix. The reproductive value of a breeding adult (v) is given by the elements of the left-eigenvector corresponding to the leading eigenvalue of the fitness matrix **w**, where we assume a neutral population (i.e. $x = z$; Fisher 1930; Taylor 1990; Taylor and Frank 1996). Note that the reproductive value of a quality-α breeder in a type-σ patch, $v_{\alpha\sigma}$, is given by the total number of offspring she has, $f_{\alpha\sigma}$, times the reproductive value of each offspring, denoted by $V_{\alpha\sigma}$. Therefore, the reproductive value of an offspring of a quality-α individual in a type-σ patch is simply given by

$$V_{\alpha\sigma} = \frac{v_{\alpha\sigma}}{f_{\alpha\sigma}}. \quad \text{(D2)}$$

The reproductive success of a focal quality-α breeder in a type-σ patch via offspring that remain in the native patch is then given by

$$w^{\phi}_{\alpha\sigma\to\eta\gamma} = f_{\alpha\sigma}(z_{\mathrm{H}\sigma}, z_{\mathrm{L}\sigma}) w^{\phi}_{\sigma} p_{\sigma\to\gamma}. \quad \text{(D3)}$$

This is simply the total number of offspring produced by a focal breeder multiplied by the probability that the offspring obtains a breeding site, which must be also multiplied by the probability that the patch becomes a type-γ patch. Finally, the philopatric component of a breeder's reproductive value is given by

$$v^{\phi}_{\alpha\sigma} = \sum_{\gamma\in\{\mathrm{R,P}\}}\sum_{\eta\in\{\mathrm{H,L}\}} w^{\phi}_{\alpha\sigma\rightarrow\eta\gamma}v_{\eta\gamma}. \quad \text{(D4)}$$

This is the reproductive success of each breeder times the reproductive value of each successful offspring according to their class.

Appendix E. Relatedness

To determine the relatedness coefficient between the high- and low-quality individuals I define recursion equations that describe how the genetic structure of the population change from one generation to the next (Bulmer 1994; Rodrigues and Gardner 2013a,b). The probability that a resource-rich patch (or a resource-poor patch) was a resource-rich patch in the previous generation is $p_{\mathrm{R}\rightarrow\mathrm{R}}$ (or $p_{\mathrm{P}\rightarrow\mathrm{R}}$), while the probability that it was a low-quality patch in the previous generation is $p_{\mathrm{R}\rightarrow\mathrm{P}}$ (or $p_{\mathrm{P}\rightarrow\mathrm{P}}$). The probability that two adults sampled at random are both born in a resource-rich patch (or in a resource-poor patch) is

$$\varphi_{\sigma} = \left(\frac{(f_{\mathrm{H}\sigma}+f_{\mathrm{L}\sigma})(1-d)}{(f_{\mathrm{H}\sigma}+f_{\mathrm{L}\sigma})(1-d)+\big(p(f_{\mathrm{HR}}+f_{\mathrm{LR}})+(1-p)(f_{\mathrm{HP}}+f_{\mathrm{LP}})\big)(1-c)}\right)^{2}. \quad \text{(E1)}$$

The probability that a philopatric juvenile is offspring of the high-quality breeder in a resource-rich patch (or in a resource-poor patch) is $U_{\mathrm{HR}} = f_{\mathrm{HR}}/(f_{\mathrm{HR}}+f_{\mathrm{LP}})$ (or $U_{\mathrm{HP}} = f_{\mathrm{HP}}/(f_{\mathrm{HP}}+f_{\mathrm{LP}})$), and the probability that a philopatric juvenile is offspring of the low-quality breeder in a resource-rich patch (or in a resource-poor patch) is $U_{\mathrm{LR}} = f_{\mathrm{LR}}/(f_{\mathrm{HR}}+f_{\mathrm{LR}})$ (or $U_{\mathrm{LP}} = f_{\mathrm{LP}}/(f_{\mathrm{HP}}+f_{\mathrm{LP}})$). In resource-rich patches (or in resource-poor patches), with probability U_{HR}^2 (or U_{HP}^2) and with probability U_{LR}^2 (or U_{LP}^2) two juveniles sampled at random are siblings, in which case their relatedness is 1. In resource-rich patches (or in resource-poor patches), with

probability $2U_{\mathrm{HR}}U_{\mathrm{LR}}$ (or $2U_{\mathrm{HP}}U_{\mathrm{LP}}$) two juveniles sampled at random are not siblings, in which case their relatedness is r_{R} (or r_{P}). The recursion equations are the given by

$$r_\sigma' = \sum_{\gamma\in\{\mathrm{R,P}\}} p_{\gamma|\sigma}\, \varphi_\gamma \left({U_{\mathrm{H}\gamma}}^2 + {U_{\mathrm{L}\gamma}}^2 + 2U_{\mathrm{H}\gamma}U_{\mathrm{L}\gamma}r_\gamma\right) \quad ,\sigma \in \{\mathrm{R,P}\} \tag{E2}$$

At equilibrium, the relatedness coefficients among breeders will not change between successive generations, in which case $r_{\mathrm{R}}' = r_{\mathrm{R}}$, and $r_{\mathrm{P}}' = r_{\mathrm{P}}$. We can then solve this system of equation to find the relatedness coefficients among breeders.

Appendix F. Optimal behaviour and convergence stability

Here I determine the convergence stability (Christiansen 1991; Eshel 1996; Taylor 1996) of the optimal competitive effort strategies. To determine if a set of optimal competitive effort strategies is convergence stable we define the matrix:

$$\left.\begin{pmatrix}
\frac{\partial(S_{\mathrm{HR}}|_{x=z})}{\partial z_{\mathrm{HR}}} & \frac{\partial(S_{\mathrm{HR}}|_{x=z})}{\partial z_{\mathrm{LR}}} & \frac{\partial(S_{\mathrm{HR}}|_{x=z})}{\partial z_{\mathrm{HP}}} & \frac{\partial(S_{\mathrm{HR}}|_{x=z})}{\partial z_{\mathrm{LP}}} \\
\frac{\partial(S_{\mathrm{LR}}|_{x=z})}{\partial z_{\mathrm{HR}}} & \frac{\partial(S_{\mathrm{LR}}|_{x=z})}{\partial z_{\mathrm{LR}}} & \frac{\partial(S_{\mathrm{LR}}|_{x=z})}{\partial z_{\mathrm{HP}}} & \frac{\partial(S_{\mathrm{LR}}|_{x=z})}{\partial z_{\mathrm{LP}}} \\
\frac{\partial(S_{\mathrm{HP}}|_{x=z})}{\partial z_{\mathrm{HR}}} & \frac{\partial(S_{\mathrm{HP}}|_{x=z})}{\partial z_{\mathrm{LR}}} & \frac{\partial(S_{\mathrm{HP}}|_{x=z})}{\partial z_{\mathrm{HP}}} & \frac{\partial(S_{\mathrm{HP}}|_{x=z})}{\partial z_{\mathrm{LP}}} \\
\frac{\partial(S_{\mathrm{LP}}|_{x=z})}{\partial z_{\mathrm{HR}}} & \frac{\partial(S_{\mathrm{LP}}|_{x=z})}{\partial z_{\mathrm{LR}}} & \frac{\partial(S_{\mathrm{LP}}|_{x=z})}{\partial z_{\mathrm{HP}}} & \frac{\partial(S_{\mathrm{LP}}|_{x=z})}{\partial z_{\mathrm{LP}}}
\end{pmatrix}\right|_{z=z^*} . \tag{F1}$$

where $S_{\rho\sigma}$ represents the left-hand side of Hamilton's rule for the expression of the behaviour of each individual depending on its status and patch type. The set of optimal strategies (z_{HR}^*, z_{LR}^*, z_{HP}^*, and z_{LP}^*) are convergence stable if the eigenvalues of matrix (F1) have negative real parts (Otto and Day 2007).

Appendix G. Fecundity and social interactions

G1. General behavioural function

In general, the fecundity of a focal individual depends on the resources available in the local patch, denoted by μ_σ, on its personal quality, denoted by $q_{\rho\sigma}$, and on social interactions, denoted by $s_{\rho\sigma}$. Thus, the fecundity of a focal class-ρ individual in a type-σ patch is given by

$$f_{\rho\sigma} = \mu_\sigma\left(q_{\rho\sigma} + s_{\rho\sigma}\right). \quad \text{(G1.1)}$$

As described in the main text, I assume that the behavioural function for social interactions depends on a personal component, denoted by Ψ, and on a group component, denoted by Θ. The personal component depends on the phenotype $x_{\rho\sigma}$ of the focal individual, whereas the group component depends on the phenotype of all individuals in the group, which I represent by the vector of phenotypes $\boldsymbol{x}_\sigma$. Thus, the social interactions term take the following functional form:

$$s_{\rho\sigma} = \Psi\left(x_{\rho\sigma}\right)\Theta(\boldsymbol{x}_\sigma). \quad \text{(G1.2)}$$

The fecundity cost of the behaviour is then given by the effect of the focal's phenotype on the personal component of the social interactions. Thus

$$C_{\rho\sigma} = -\frac{\partial f_{\rho\sigma}}{\partial x_{\rho\sigma}} = -\mu_\sigma \frac{\partial \Psi(x_{\rho\sigma})}{\partial x_{\rho\sigma}} \Theta(\boldsymbol{x}_\sigma). \quad \text{(G1.3)}$$

The fecundity benefit of the behaviour is given by the effect of the actor's phenotype on the group component of the social interactions. Thus

$$B_{\rho\sigma} = \frac{\partial f_{\rho\sigma}}{\partial x_{\alpha\sigma}} = \mu_\sigma \Psi(z_{\rho\sigma}) \frac{\partial \Theta(x_\sigma)}{\partial x_{\alpha\sigma}}. \quad \text{(G1.4)}$$

In the main text, I consider that the behavioural functions give form to a competitive effort game between group mates. Below, I explore different functional forms for the social interactions among group mates.

G2. Investment in a public good

Let us consider the evolution of investment in a public good. I assume that investment in a public good is costly to the actor, but increases the pool of publically available resources. The pool of available resources is assumed to be fairly distributed among all social partners, including the contributor. Investment in the public good is costly, in the sense that the actor's ability to access the pool of publically available resources is impaired by the expression of the behaviour. Given these assumptions, the behavioural function of the social interactions takes the form

$$s_{\rho\sigma} = (1 - x_{\rho\sigma}) \frac{1}{n_\sigma} \sum_{\eta \in \{H,L\}} x_{\eta\sigma}, \quad \text{(G2.1)}$$

where: $\Psi(x_{\rho\sigma}) = (1 - x_{\rho\sigma})$; $\Theta(\boldsymbol{x}_\sigma) = \frac{1}{n_\sigma} \sum_{\eta \in \{H,L\}} x_{\eta\sigma}$; and $n_\sigma = 2$ is the number of individuals in the patch. From equations (G1.3) and (G2.1), the cost of the investment in the public good is given by

$$C_{\rho,\sigma} = \frac{1}{n_\sigma} \sum_{\eta \in \{H,L\}} x_{\eta,\sigma}, \quad \text{(G2.2)}$$

which means that the cost paid by the actor is given by the amount of public good available to the focal recipient. From equations (G1.4) and (G2.1), we find that the benefit provided by the focal individual to social partners is given by

$$B_{\rho\eta,\sigma} = \left(1 - x_{\eta,\sigma}\right)\frac{1}{n_{\sigma}}. \qquad \text{(G2.3)}$$

In Fig. G2.1 and Fig. G2.2 I present the results for this behavioural function. We find that the results are qualitatively similar to those obtained in the main text for a different behavioural function. In particular, we find a patch productivity and inequality are negatively correlated.

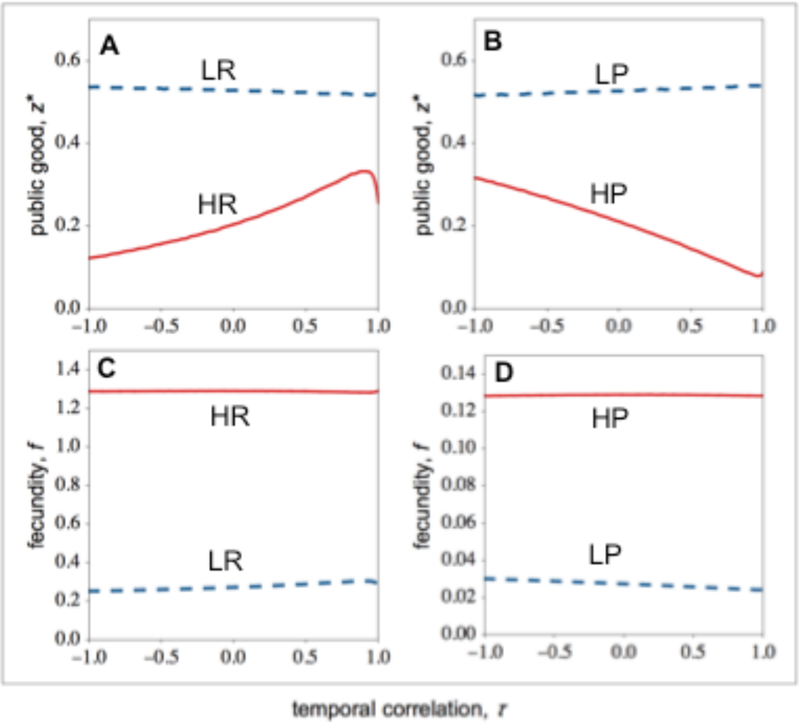

Figure G2.1. Optimal investment in public good (z^*) and fecundity (f) of high- (solid lines) and low-quality (dashed lines) individuals as a function of the temporal correlation (τ). [A,C] In resource-rich patches, average investment in the public good increases as the environment becomes more stable. [B,D] In resource-poor patches, average investment in the public good decreases as the environment become more stable. Environmental stability leads to higher average fecundity in resource-rich patches, while it leads to lower average fecundity in resource-poor patches. Parameter values: $c = 0.9$, $d = 0.1$, $p = 0.5$, $q_{HR} = q_{HP} = 1.0$, $q_{LR} = q_{LP} = 0.1$, $\mu_R = 1.0$, $\mu_P = 0.1$.

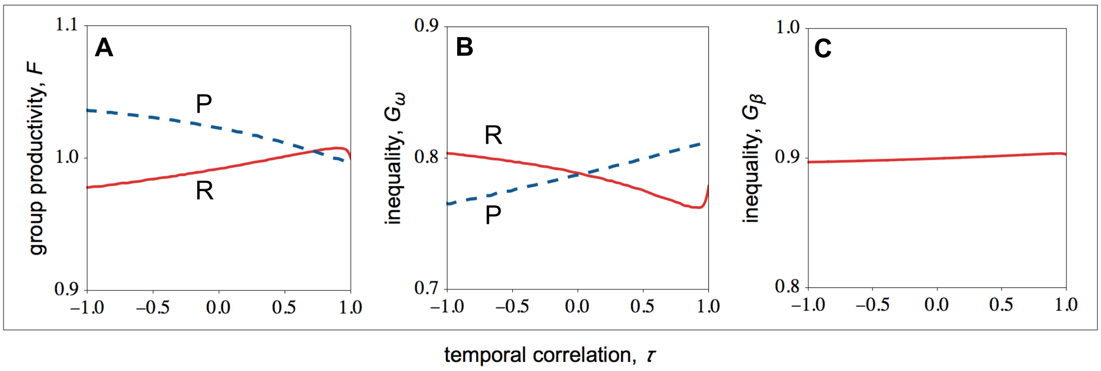

Figure G2.2. Relative group productivity (F), within-group inequality (G_{ω}), and between-group inequality (G_{β}) as a function of the temporal correlation (τ). Group productivity is plotted relative to the baseline group productivity in each patch. Baseline group productivity is given by group productivity in a stable environment (i.e. $F_0 = F(\tau = 1)$). Thus, $F = F(\tau)/F_0$. [A] Environmental stability in resource-availability leads to higher productivity in resource-rich patches, but to lower productivity in resource-poor patches. [B] In resource-rich patches, inequality decreases as the environment becomes more stable. In resource-poor patches, inequality increases as the environment becomes more stable. [C] Productivity in resource-rich patches increases, relative to that of resource-poor patches, as the environment becomes more stable. Parameter values: $c = 0.9$, $d = 0.1$, $p = 0.5$, $q_{HR} = q_{HP} = 1.0$, $q_{LR} = q_{LP} = 0.1$, $\mu_R = 1.0$, $\mu_P = 0.1$.

G3. Simple competitive effort

In the main text, I considered a competitive effort behavioural function. In this section, I consider a variation of this behavioural function. More specifically, I consider a case in which the fraction of resources obtained by the focal individual is not relative to the average competitive effort in the local group, as in the main text (cf Frank 1994). Thus, I assume that competitive effort, denoted by $x_{\rho\sigma}$, directly increases the fraction of resources obtained by the focal individual, but it reduces the focal's contribution to the common pool of resources. The fecundity of a focal individual through social interactions is then given by

$$s_{\rho\sigma} = x_{\rho\sigma}\left(1 - \frac{1}{n_\sigma}\sum_{\eta\in\{\mathrm{H,L}\}} x_{\eta\sigma}\right). \qquad \text{(G3.1)}$$

where: $\Psi(x_{\rho\sigma}) = x_{\rho\sigma}$ and $\Theta(\boldsymbol{x}_\sigma) = \left(1 - \frac{1}{n_\sigma}\sum_{\eta\in\{\mathrm{H,L}\}} x_{\eta\sigma}\right)$. From equations (G1.3) and (G3.1), the cost of competitive effort becomes

$$C_{\rho\sigma} = -\left(1 - \frac{1}{n_\sigma}\sum_{\eta\in\Omega_\sigma} x_{\eta\sigma}\right). \qquad \text{(G3.2)}$$

Note that the cost is negative. That is the investment in competitive effort confers a direct benefit to the recipient that is proportional to the pool of resources available to the recipient. From equations (G1.4) and (G3.1), the benefit provided by the focal actor to a group mate is given by

$$B_{\alpha\rho,\sigma} = -z_{\rho,\sigma}\frac{1}{n_\sigma}. \qquad \text{(G3.3)}$$

Note that the benefit is negative. That is an additional investment in competitive effort reduces the amount of resources publically available to each of the social partners. In Fig. G3.1 and Fig. G3.2, I present the results for this behavioural function. We find that the results are qualitatively similar to those obtained in the main text for a different behavioural function. In particular, we find a patch productivity and inequality are negatively correlated.

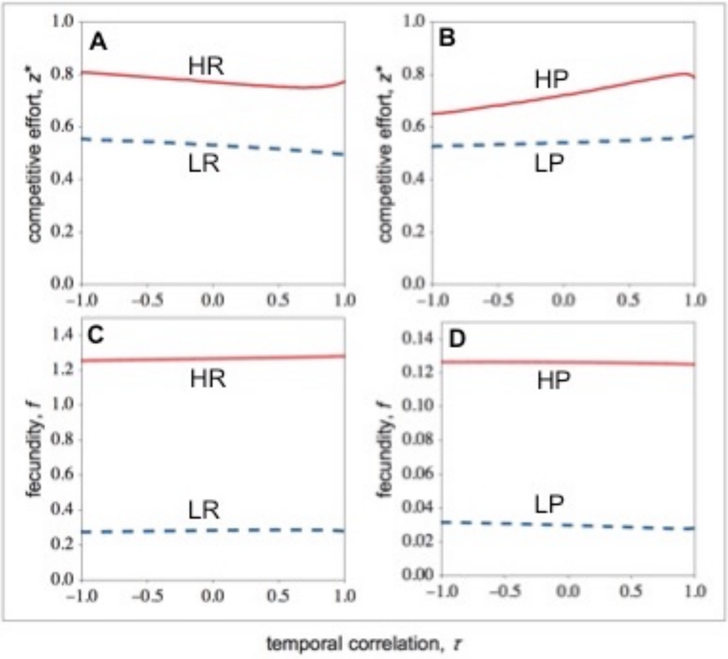

Figure G3.1. Competitive effort (z) and fecundity (f) of high- (solid lines) and low-quality (dashed lines) individuals as a function of the temporal correlation (τ). [A,C] In resource-rich patches, temporal stability in resource-availability decreases average investment in competitive effort. [C,D] In resource-poor patches, temporal stability in resource-availability increases average investment in competitive effort. Temporal stability leads to higher average fecundity in resource-rich patches, while it leads to lower average fecundity in resource-poor patches. Parameter values: $c = 0.9$, $d = 0.1$, $p = 0.5$, $q_{HR} = q_{HP} = 1.0$, $q_{LR} = q_{LP} = 0.1$, $\mu_R = 1.0$, $\mu_P = 0.1$.

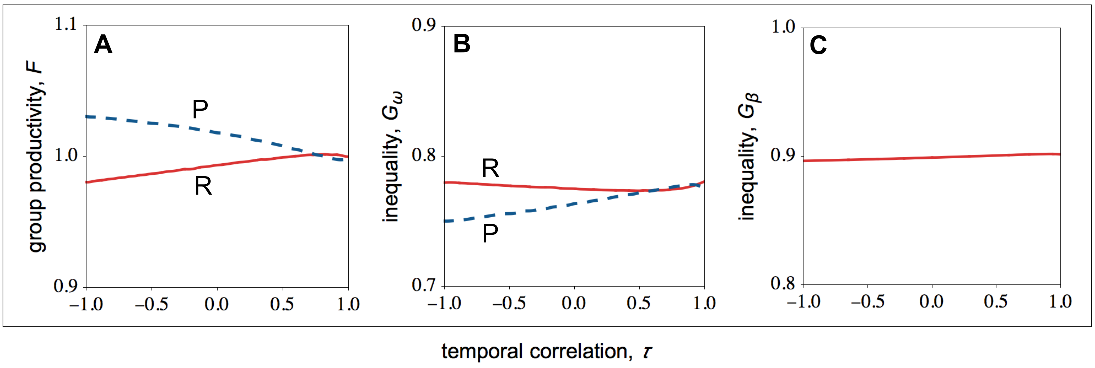

Figure G3.2. Relative group productivity (F), within-group inequality (G_{ω}), and between-group inequality (G_{β}) as a function of the temporal correlation (τ). Group productivity is plotted relative to the baseline group productivity for each patch. Baseline group productivity is given by group productivity in a stable environment (i.e. $F_0 = F(\tau = 1)$). Thus, $F = F(\tau)/F_0$. [A] Temporal stability in resource-availability leads to lower productivity in resource-rich patches, while it leads to higher productivity in resource-poor patches. [B] In resource-rich patches, inequality decreases as the environment becomes more stable. In resource-poor patches, inequality increases as the environment becomes more stable. [C] Productivity in resource-rich patches increases, relative to that of resource-poor patches, as the environment becomes more stable such that inequality between patch types increases. Parameter values: $c = 0.9$, $d = 0.1$, $p = 0.5$, $q_{HR} = q_{HP} = 1.0$, $q_{LR} = q_{LP} = 0.1$, $\mu_R = 1.0$, $\mu_P = 0.1$.

Appendix H. Early-life acquisition of individual quality

Reproductive success and reproductive value

We assume that mothers produce a fraction Q of high-quality offspring, and a fraction $1 - Q$ of low-quality offspring, irrespective of a mother's quality. As a result, the total proportion of high-quality offspring competing for breeding sites in any focal patch is given by Q while the proportion of low-quality offspring competing for breeding sites is given by $1 - Q$. Thus, the probability that the focal patch becomes a type-$\kappa\lambda$ patch is given by

$$p_{\kappa\lambda} = \binom{n}{m} Q^m (1 - Q)^{n-m}, \tag{H1}$$

where κ and λ are the quality of each breeder, m is the number of high-quality breeders in the patch, and n is the patch size, which we set to 2.

Given the state of the patch, we can now define the number of breeding spots for each type of offspring quality. Let us denote the number of quality-i breeding spots in a type-ij patch by o_{ij}. Thus,

$$o_{ij} = \begin{cases} 2 & , i = \mathrm{H}, j = \mathrm{H} \\ 1 & , i = \mathrm{H}, j = \mathrm{L} \\ 1 & , i = \mathrm{L}, j = \mathrm{H} \\ 2 & , i = \mathrm{L}, j = \mathrm{L} \end{cases}. \tag{H2}$$

Let us define the set $\Omega = \{\mathrm{HH}, \mathrm{HL}, \mathrm{LL}\}$, which includes the set of all patch types, noting that the type-HL patch is the same as the type-LH patch. The reproductive success of a quality-ρ

mothers with a quality-μ mother as social partner in a type-π patch (where π denotes the resources available in the patch, i.e. either ‘R’ or ‘P’) through offspring that remain philopatric is given by

$$w^{\phi}_{\rho\mu,\pi}\left(x_{\mathrm{H}\pi}, x_{\mathrm{L}\pi}\right) = \frac{f_{\rho\mu,\pi}(1-d)}{\left(f_{\rho\mu,\pi}+f_{\mu\rho,\pi}\right)(1-d)+\left(\sum_{\gamma\in\{\mathrm{R,P}\}} p_{\gamma} \sum_{\mu\rho\in\Omega} p_{\mu\rho}\left(f_{\mu\rho,\gamma}+f_{\mu\rho,\gamma}\right)\right)d(1-c)}. \quad \text{(H3)}$$

The reproductive success of a quality-ρ mothers with a quality-μ mother as social partner in a type-π patch through offspring that disperse away from the natal patch and arrive at a type-ω patch with a quality-ε and a quality-ϱ individual is given by

$$w^{\delta}_{\rho\mu,\pi\to\varepsilon\varrho,\omega}\left(x_{\mathrm{H}\pi}, x_{\mathrm{L}\pi}\right) = \frac{f_{\rho\mu,\pi} d(1-c)}{\left(f_{\varepsilon\varrho,\omega}+f_{\varrho\varepsilon,\omega}\right)(1-d)+\left(\sum_{\gamma\in\{\mathrm{R,P}\}} p_{\gamma} \sum_{\mu\rho\in\Omega} p_{\mu\rho}\left(f_{\mu\rho,\gamma}+f_{\mu\rho,\gamma}\right)\right)d(1-c)}. \quad \text{(H4)}$$

That is, the focal quality-ρ mother produces $f_{\rho\mu,\pi}$, a fraction d of which disperse, and where only a fraction $1 - c$ survive dispersal.

The reproductive success of a quality-ρ mother with a quality-μ mother as a social partner in a type-π patch through offspring that become quality-κ mothers with quality-λ mothers as social partners in a type-ψ patch is given by

$$\begin{aligned} w_{\rho\mu,\pi\to\kappa\lambda,\psi} = \; & w^{\phi}_{\rho\mu,\pi} p_{\pi\to\psi} p_{\kappa\lambda} o_{\kappa\lambda} \\ & +\left(\sum_{\gamma\in\{\mathrm{R,P}\}} p_{\gamma} p_{\gamma\to\psi} \sum_{\varepsilon\varrho\in\Omega} p_{\varepsilon\varrho} w^{\delta}_{\rho\mu,\pi\to\varepsilon\varrho,\gamma}\right) p_{\kappa\lambda} o_{\kappa\lambda} \end{aligned}. \quad \text{(H5)}$$

That is, a quality-ρ focal mother with a quality-μ social partner in a type-π patch wins a breeding spot in the local patch with probability $w^{\phi}_{\rho\mu,\pi}$. With probability $p_{\pi\to\psi}$ the type-π

patch becomes a type-ψ patch. The probability that the patch becomes a patch with a quality-κ and a quality-λ breeder is given by $p_{\kappa\lambda}$, in which case there are $o_{\kappa\lambda}$ quality-κ breeding spots available.

The focal quality-ρ breeder also derives fitness from offspring that disperse from the local patch. With probability p_{γ} the dispersed offspring arrive at a type-γ patch, which becomes a type-ψ patch with probability $p_{\gamma\rightarrow\psi}$. With probability $p_{\varepsilon\varrho}$ the type-γ patch has a quality-ε breeder and a quality-ϱ breeder, in which case the probability that the focal quality-ρ breeder wins a breeding spot through the dispersed offspring is given by $w^{\delta}_{\rho\mu,\pi\rightarrow\varepsilon\varrho,\gamma}$. Finally, the type-$\gamma$ patches accommodate a quality-κ and a quality-λ breeder with probability $p_{\kappa\lambda}$, in which case there are $o_{\kappa\lambda}$ quality-κ breeding spots.

From equations (A2-A4), we can then define the 8×8 fitness matrix, denoted by **w**, as

$$\mathbf{w} = \left(w_{\rho\mu,\pi\rightarrow\kappa\lambda,\psi}\right)_{8\times8}. \tag{H6}$$

From the fitness matrix, we find the reproductive value of each mother by calculating the left eigenvector associated with the eigenvalue one.

<u>Relatedness</u>

The relatedness coefficient depends on the previous state of the patch. The probability that a type-κ patch was a type-π patch is given by $p_{\pi\rightarrow k}$. The probability that the type-π patch was a type-$\mu\rho$ is given by $p_{\mu\rho}$. The probability that two random offspring are both offspring of the

quality-ρ breeder is given by $\left(w^{\phi}_{\rho\mu,\pi}\right)^2$ and the probability that two random offspring are both offspring of the quality-μ breeder is given by $\left(w^{\phi}_{\mu\rho,\pi}\right)^2$, in both cases the relatedness coefficient is 1. The probability that both juveniles are offspring of different parents is given by $2w^{\phi}_{\rho\mu,\pi}w^{\phi}_{\mu\rho,\pi}$, in which case the relatedness coefficient is $r_{\mu\rho,\pi}$. The recursion equation that gives the relatedness coefficient between a quality-i and a quality-j breeder in a type-κ patch is given by

$$r_{\mathrm{ij},\kappa} = \sum_{\pi\in\{R,L\}} p_{\pi\to\mathrm{k}} \left(\sum_{\mu\rho\in\Omega} p_{\mu\rho} \left(\left(w^{\phi}_{\rho\mu,\pi}\right)^2 + \left(w^{\phi}_{\mu\rho,\pi}\right)^2 + 2w^{\phi}_{\rho\mu,\pi}w^{\phi}_{\mu\rho,\pi}r_{\mu\rho,\pi}\right)\right). \qquad \text{(H7)}$$

This gives a system of recursion equations that can be solved for the relatedness coefficients in each patch.

<u>Hamilton's rule</u>

To define Hamilton's rule, it is useful to define reproductive value of a breeder through offspring that remain in the local patch (i.e. the philopatric component of reproductive value). With probability $p_{\pi\to\omega}$ the focal type-π patch becomes a type-ω patch. With probability $w^{\phi}_{\rho\mu,\pi}$ an offspring of the focal breeder wins a breeding site. Finally, with probability $p_{\kappa\lambda}$ the focal patch accommodates a quality-κ and a quality-λ breeder, in which case there are $o_{\kappa\lambda}$ quality-κ breeding sites available, and in which case the focal breeder derives a reproductive value $v_{\kappa\lambda,\omega}$. Collecting all these terms together, we obtain

$$v^{\phi}_{\rho\mu,\pi} = \sum_{\omega\in\{\mathrm{R,P}\}} p_{\pi\to\omega}\left(\sum_{\kappa\in\{\mathrm{H,L}\}} \sum_{\lambda\in\{\mathrm{H,L}\}} w^{\phi}_{\rho\mu,\pi} p_{\kappa\lambda} o_{\kappa\lambda}\right). \qquad \text{(H8)}$$

It is also useful to define the probability that a single offspring remains in the local patch and wins a breeding site, which is given by

$$W^{\phi}_{\rho\mu,\pi}(x_{\mathrm{H}\pi}, x_{\mathrm{L}\pi}) = \frac{(1-d)}{(f_{\rho\mu,\pi}+f_{\mu\rho,\pi})(1-d)+(\sum_{\gamma\in\{\mathrm{R,P}\}} p_{\gamma} \sum_{\mu\rho\in\Omega} p_{\mu\rho})d(1-c)}. \quad \text{(H9)}$$

Hamilton's rule, is then given by

$$-C_{\mathrm{ij},\kappa}V_{\mathrm{ij},\kappa} + B_{\mathrm{ij},\kappa}V_{\mathrm{ij},\kappa}r_{\mathrm{ij},\kappa} - (B_{\mathrm{ij},\kappa} - C_{\mathrm{ij},\kappa})W^{\phi}_{\mathrm{ij},\kappa}\left(v^{\phi}_{\mathrm{ij},\kappa} + v^{\phi}_{\mathrm{ji},\kappa}r_{\mathrm{ij},\kappa}\right) > 0. \quad \text{(H10)}$$

Appendix I. Patch size

In the main text, I assumed that each patch has exactly two breeders, one high- and one low-quality breeder. Here, I consider that patch size can be greater than two, but I keep the proportion of high- and low-quality breeders in each patch constant, such that the number of high-quality and low-quality breeders is given by a, with $a = n/2$. This extension of the model does not change the calculation of reproductive values, as the increase in competition for breeder sites, owing to the increase in the number of breeders, is exactly compensated by the increase in the number of breeding sites available. However, this extension of the model does change the calculation of relatedness.

The probability that a type-κ patch was a type-π patch is given by $p_{\pi\to\mathrm{k}}$. The probability that two random offspring are both philopatric offspring is given by φ_{π}. The probability that two random offspring are both offspring of a focal quality-ρ breeder is given by $f_{\rho\pi}/(af_{\mathrm{H}\pi} + (1-a)f_{\mathrm{L}\pi})$. If the two random philopatric offspring do not share the same mother, then the coefficient of relatedness is given by r_{π}. The recursion equation that gives the relatedness coefficient in a type-κ patch is then given by

$$r_{\kappa}' = \sum_{\pi \in \{R,L\}} p_{\pi \to \kappa} \varphi_{\pi} \left(\sum_{\rho \in \{H,L\}} a \left(\frac{f_{\rho\pi}}{a f_{H\pi} + a f_{L\pi}} \right)^2 + \left(1 - \sum_{\rho \in \{H,L\}} a \left(\frac{f_{\rho\pi}}{a f_{H\pi} + a f_{L\pi}} \right)^2 \right) r_{\pi} \right). \quad \text{(I1)}$$

This gives a system of recursion equations that can be solved for the relatedness coefficients in each patch.

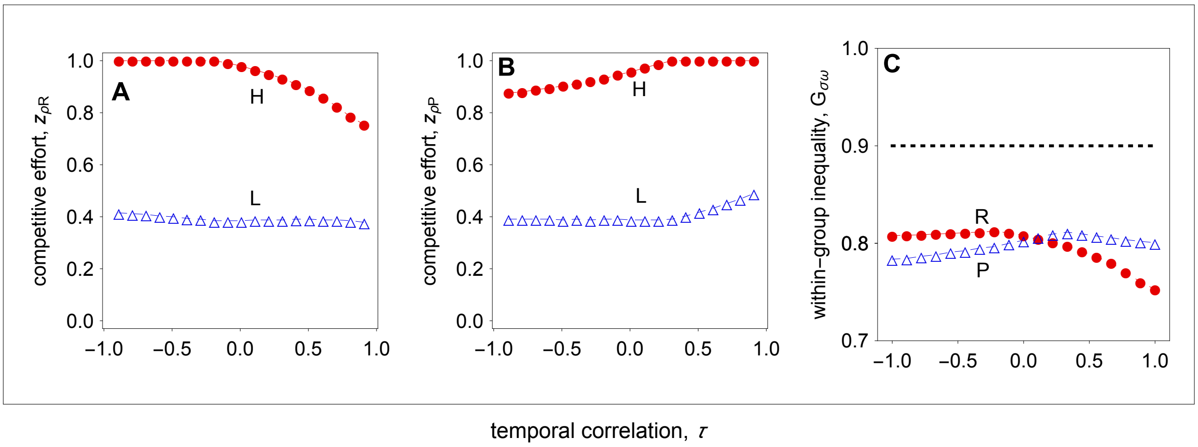

Figure I1. A. Competitive effort of high-quality and low-quality individuals as a function of the temporal correlation in resource-rich patches. **B.** Competitive effort of high-quality and low-quality individuals as a function of the temporal correlation in resource-poor patches. **C.** Coefficient of within-group inequality in resource-rich and resource-poor patches as a function of the temporal correlation. Parameter values: $n = 4$, $d = 0.1$, $c = 0.5$, $p = 0.5$, $q_{HR} = q_{HP} = 1.0$, $q_{LR} = q_{LP} = 0.1$, $\mu_R = 1.0$, $\mu_P = 0.1$.

References

Bulmer, M.G. 1994. *Theoretical evolutionary ecology*. Sinauer, Sunderland, Massachusetts.

Christiansen, F.B. 1991. On conditions for evolutionary stability for a continuously varying character. *Am. Nat.* 138:37–50.

Eshel, I. 1996. On the changing concept of evolutionary population stability as a reflection of a changing point of view in the quantitative theory of evolution. *J. Math. Biol.* 34:485–510.

Fisher, R.A. 1930. *The genetical theory of natural selection*. Clarendon Press, Oxford, UK.

Frank, S.A. 1994. Kin selection and virulence in the evolution of protocells and parasites. *Proc. R. Soc. B* 258:153–161.

Rodrigues, A.M.M., and Gardner, A. 2012. Evolution of helping and harming in heterogeneous populations. *Evolution* 66:2065-2079.

Rodrigues, A.M.M., and Gardner, A. 2013a. Evolution of helping and harming in viscous populations when group size varies. *Am. Nat.* 181:609-622.

Rodrigues, A. M. M., and Gardner, A. 2013b. Evolution of helping and harming in heterogeneous groups. *Evolution* 67:2284-2298.

Rodrigues, A.M.M., and Gardner, A. 2016. The constant philopater hypothesis: a new life history invariant for dispersal evolution. *J. Evol. Biol.* 29:153–166.

Taylor, P.D. 1990. Allele-Frequency change in a class-structured population. *Am. Nat.* 135:95–106.

Taylor, P.D. 1996 Inclusive fitness arguments in genetic models of behaviour. *J. Math. Biol.* 34:654–674.

Taylor, P.D., and Frank, S.A. 1996. How to make a kin selection model. *J. Theor. Biol.* 180:27–37.

Otto, S., and Day, T. 2007. *A biologist's guide to mathematical modeling in ecology and evolution*. Princeton University Press, Princeton, NJ.
