# Supplementary material for: Resource availability and adjustment of social behaviour influence patterns of inequality and productivity across societies
Source: PeerJ. 2018 Oct 2;6:e5488. doi: 10.7717/peerj.5488 (PMC6173167; doi:10.7717/peerj.5488)

Wealth and adjustment of social behaviour influence patterns of inequality and productivity across animal societies

Mathematica Code

Antonio M. M. Rodrigues
Department of Zoology, University of Cambridge, Cambridge CB2 3EJ, United Kingdom

Ecology

In[9872]:=
```
Clear[α, p]
Eigensystem[(pRR  1 - pPP
             1 - pRR  pPP)]
Solve[{p == (1 - pRR)/(2 - (pRR + pPP)), τ == pRR - (1 - pPP)}, {pRR, pPP}]
```

Out[9873]= $\left\{\{1, -1 + pPP + pRR\}, \left\{\left\{-\frac{1 - pPP}{-1 + pRR}, 1\right\}, \{-1, 1\}\right\}\right\}$

Out[9874]= $\{\{pRR \to 1 - p + p\,\tau,\ pPP \to p + \tau - p\,\tau\}\}$

Relatedness

In[5118]:=
```
Clear[fLR, fLP]
Solve[
 {rR == (1 - p + p τ) φR ((fHR/(fHR + fLR))^2 + (fLR/(fHR + fLR))^2 + 2 fHR/(fHR + fLR) fLR/(fHR + fLR) rR) +
     (1 - (1 - p + p τ)) φP
      ((fHP/(fHP + fLP))^2 + (fLP/(fHP + fLP))^2 + 2 fHP/(fHP + fLP) fLP/(fHP + fLP) rP),
   rP == (p + τ - p τ) φP ((fHP/(fHP + fLP))^2 + (fLP/(fHP + fLP))^2 + 2 fHP/(fHP + fLP) fLP/(fHP + fLP) rP) +
     (1 - (p + τ - p τ)) φR
      ((fHR/(fHR + fLR))^2 + (fLR/(fHR + fLR))^2 + 2 fHR/(fHR + fLR) fLR/(fHR + fLR) rR)},
 {rR, rP}]
```

Out[5119]=

$$\Bigg\{\Bigg\{rR \to -\Bigg(\Bigg(-\left(1-\frac{2\,fHP\,fLP\,(p+\tau-p\,\tau)\,\varphi P}{(fHP+fLP)^2}\right)\left(-\frac{fHP^2\,(p-p\,\tau)\,\varphi P}{(fHP+fLP)^2}-\frac{fLP^2\,(p-p\,\tau)\,\varphi P}{(fHP+fLP)^2}-\frac{fHR^2\,(1-p+p\,\tau)\,\varphi R}{(fHR+fLR)^2}-\frac{fLR^2\,(1-p+p\,\tau)\,\varphi R}{(fHR+fLR)^2}\right)-\frac{1}{(fHP+fLP)^2}\,2\,fHP\,fLP\,(p-p\,\tau)\,\varphi P\left(-\frac{fHP^2\,(p+\tau-p\,\tau)\,\varphi P}{(fHP+fLP)^2}-\frac{fLP^2\,(p+\tau-p\,\tau)\,\varphi P}{(fHP+fLP)^2}-\frac{fHR^2\,(1-p-\tau+p\,\tau)\,\varphi R}{(fHR+fLR)^2}-\frac{fLR^2\,(1-p-\tau+p\,\tau)\,\varphi R}{(fHR+fLR)^2}\right)\Bigg)\Bigg/$$

$$\left(\frac{4\,fHP\,fHR\,fLP\,fLR\,(p-p\,\tau)\,(1-p-\tau+p\,\tau)\,\varphi P\,\varphi R}{(fHP+fLP)^2\,(fHR+fLR)^2}-\left(1-\frac{2\,fHP\,fLP\,(p+\tau-p\,\tau)\,\varphi P}{(fHP+fLP)^2}\right)\left(1-\frac{2\,fHR\,fLR\,(1-p+p\,\tau)\,\varphi R}{(fHR+fLR)^2}\right)\right)\Bigg),$$

$$rP \to -\big((fHP^2\,fHR^2\,p\,\varphi P + fHR^2\,fLP^2\,p\,\varphi P + 2\,fHP^2\,fHR\,fLR\,p\,\varphi P + 2\,fHR\,fLP^2\,fLR\,p\,\varphi P +$$
$$fHP^2\,fLR^2\,p\,\varphi P + fLP^2\,fLR^2\,p\,\varphi P + fHP^2\,fHR^2\,\tau\,\varphi P + fHR^2\,fLP^2\,\tau\,\varphi P +$$
$$2\,fHP^2\,fHR\,fLR\,\tau\,\varphi P + 2\,fHR\,fLP^2\,fLR\,\tau\,\varphi P + fHP^2\,fLR^2\,\tau\,\varphi P + fLP^2\,fLR^2\,\tau\,\varphi P -$$
$$fHP^2\,fHR^2\,p\,\tau\,\varphi P - fHR^2\,fLP^2\,p\,\tau\,\varphi P - 2\,fHP^2\,fHR\,fLR\,p\,\tau\,\varphi P - 2\,fHR\,fLP^2\,fLR\,p\,\tau\,\varphi P -$$
$$fHP^2\,fLR^2\,p\,\tau\,\varphi P - fLP^2\,fLR^2\,p\,\tau\,\varphi P + fHP^2\,fHR^2\,\varphi R + 2\,fHP\,fHR^2\,fLP\,\varphi R +$$
$$fHR^2\,fLP^2\,\varphi R + fHP^2\,fLR^2\,\varphi R + 2\,fHP\,fLP\,fLR^2\,\varphi R + fLP^2\,fLR^2\,\varphi R - fHP^2\,fHR^2\,p\,\varphi R -$$
$$2\,fHP\,fHR^2\,fLP\,p\,\varphi R - fHR^2\,fLP^2\,p\,\varphi R - fHP^2\,fLR^2\,p\,\varphi R - 2\,fHP\,fLP\,fLR^2\,p\,\varphi R -$$
$$fLP^2\,fLR^2\,p\,\varphi R - fHP^2\,fHR^2\,\tau\,\varphi R - 2\,fHP\,fHR^2\,fLP\,\tau\,\varphi R - fHR^2\,fLP^2\,\tau\,\varphi R -$$
$$fHP^2\,fLR^2\,\tau\,\varphi R - 2\,fHP\,fLP\,fLR^2\,\tau\,\varphi R - fLP^2\,fLR^2\,\tau\,\varphi R + fHP^2\,fHR^2\,p\,\tau\,\varphi R +$$
$$2\,fHP\,fHR^2\,fLP\,p\,\tau\,\varphi R + fHR^2\,fLP^2\,p\,\tau\,\varphi R + fHP^2\,fLR^2\,p\,\tau\,\varphi R + 2\,fHP\,fLP\,fLR^2\,p\,\tau\,\varphi R +$$
$$fLP^2\,fLR^2\,p\,\tau\,\varphi R - 2\,fHP^2\,fHR\,fLR\,\tau\,\varphi P\,\varphi R - 2\,fHR\,fLP^2\,fLR\,\tau\,\varphi P\,\varphi R)\,\Big/$$
$$(-fHP^2\,fHR^2 - 2\,fHP\,fHR^2\,fLP - fHR^2\,fLP^2 - 2\,fHP^2\,fHR\,fLR - 4\,fHP\,fHR\,fLP\,fLR -$$
$$2\,fHR\,fLP^2\,fLR - fHP^2\,fLR^2 - 2\,fHP\,fLP\,fLR^2 - fLP^2\,fLR^2 + 2\,fHP\,fHR^2\,fLP\,p\,\varphi P +$$
$$4\,fHP\,fHR\,fLP\,fLR\,p\,\varphi P + 2\,fHP\,fLP\,fLR^2\,p\,\varphi P + 2\,fHP\,fHR^2\,fLP\,\tau\,\varphi P +$$
$$4\,fHP\,fHR\,fLP\,fLR\,\tau\,\varphi P + 2\,fHP\,fLP\,fLR^2\,\tau\,\varphi P - 2\,fHP\,fHR^2\,fLP\,p\,\tau\,\varphi P -$$
$$4\,fHP\,fHR\,fLP\,fLR\,p\,\tau\,\varphi P - 2\,fHP\,fLP\,fLR^2\,p\,\tau\,\varphi P + 2\,fHP^2\,fHR\,fLR\,\varphi R +$$
$$4\,fHP\,fHR\,fLP\,fLR\,\varphi R + 2\,fHR\,fLP^2\,fLR\,\varphi R - 2\,fHP^2\,fHR\,fLR\,p\,\varphi R -$$
$$4\,fHP\,fHR\,fLP\,fLR\,p\,\varphi R - 2\,fHR\,fLP^2\,fLR\,p\,\varphi R + 2\,fHP^2\,fHR\,fLR\,p\,\tau\,\varphi R +$$
$$4\,fHP\,fHR\,fLP\,fLR\,p\,\tau\,\varphi R + 2\,fHR\,fLP^2\,fLR\,p\,\tau\,\varphi R - 4\,fHP\,fHR\,fLP\,fLR\,\tau\,\varphi P\,\varphi R)\big)\Bigg\}\Bigg\}$$

```
Clear[d, rR, rP]
c = 0.9;
d = 0.1;
μR = 1;
μP = 0.1;
qHR = 1;
qLR = 0.1;
qHP = 1;
qLP = 0.1;
hlabel = {{-1, "-1.0", {0, 0.01}}, {-0.5, "-0.5", {0, 0.01}},
   {0, "0.0", {0, 0.01}}, {0.5, "0.5", {0, 0.01}},
```

```
    {1.0, "1.0", {0, 0.01}}};
vlabel = Join[{{0.0, "0.0", {0, 0.01}}},
    Table[{i, ToString[N[i]], {0, 0.01}}, {i, 2/10, .8, 2/10}],
    {{1, "1.0", {0, 0.01}}}];
pticks = Join[Table[{i, ToString[i], {0, 0.01}}, {i, -0.8, -0.2, 0.2}],
    {{0, "0.0", {0, 0.01}}},
    Table[{i, ToString[i], {0, 0.01}}, {i, 0.2, 0.8, 0.2}]];

colour = {{"#DA3232", "#12588E"}, {"#DA3232", "#12588E"}};
style1 = {Directive[RGBColor[colour[[1]][[1]]]],
    Directive[RGBColor[colour[[1]][[2]]], Dashing[{0.03, 0.05}],
     Thickness[0.015]]};
```

$$fHR := \mu R \left(qHR + \frac{xHR}{\frac{1}{2}(xHR + xLR)}\left(1 - \frac{1}{2}(xHR + xLR)\right)\right);$$

$$fLR := \mu R \left(qLR + \frac{xLR}{\frac{1}{2}(xHR + xLR)}\left(1 - \frac{1}{2}(xHR + xLR)\right)\right);$$

$$fHP := \mu P \left(qHP + \frac{xHP}{\frac{1}{2}(xHP + xLP)}\left(1 - \frac{1}{2}(xHP + xLP)\right)\right);$$

$$fLP := \mu P \left(qLP + \frac{xLP}{\frac{1}{2}(xHP + xLP)}\left(1 - \frac{1}{2}(xHP + xLP)\right)\right);$$

$$sH = \frac{fLR}{fHR};\ sL = \frac{fLP}{fHP};\ \sigma = \frac{fHP}{fHR};$$

$$\varphi R := \left(((1-d)\ (fHR + fLR))\ /\ ((1-d)\ (fHR + fLR) + (p\ (fHR + fLR) + (1-p)\ (fHP + fLP))\ d\ (1-c))\right)^2;$$

$$\varphi P := \left(((1-d)\ (fHP + fLP))\ /\ ((1-d)\ (fHP + fLP) + (p\ (fHR + fLR) + (1-p)\ (fHP + fLP))\ d\ (1-c))\right)^2;$$

$$rR[\tau\_] :=$$

$$-\left(\left(-\left(1 - \frac{2\ fHP\ fLP\ (p + \tau - p\ \tau)\ \varphi P}{(fHP + fLP)^2}\right)\right.\right.$$

$$\left(-\frac{\mathrm{fHP}^2\ (p-p\,\tau)\ \varphi\mathrm{P}}{(\mathrm{fHP}+\mathrm{fLP})^2}-\frac{\mathrm{fLP}^2\ (p-p\,\tau)\ \varphi\mathrm{P}}{(\mathrm{fHP}+\mathrm{fLP})^2}-\frac{\mathrm{fHR}^2\ (1-p+p\,\tau)\ \varphi\mathrm{R}}{(\mathrm{fHR}+\mathrm{fLR})^2}-\frac{\mathrm{fLR}^2\ (1-p+p\,\tau)\ \varphi\mathrm{R}}{(\mathrm{fHR}+\mathrm{fLR})^2}\right)-$$

$$\left(2\ \mathrm{fHP}\ \mathrm{fLP}\ (p-p\,\tau)\ \varphi\mathrm{P}\left(-\frac{\mathrm{fHP}^2\ (p+\tau-p\,\tau)\ \varphi\mathrm{P}}{(\mathrm{fHP}+\mathrm{fLP})^2}-\frac{\mathrm{fLP}^2\ (p+\tau-p\,\tau)\ \varphi\mathrm{P}}{(\mathrm{fHP}+\mathrm{fLP})^2}-\frac{\mathrm{fHR}^2\ (1-p-\tau+p\,\tau)\ \varphi\mathrm{R}}{(\mathrm{fHR}+\mathrm{fLR})^2}-\frac{\mathrm{fLR}^2\ (1-p-\tau+p\,\tau)\ \varphi\mathrm{R}}{(\mathrm{fHR}+\mathrm{fLR})^2}\right)\middle/\ (\mathrm{fHP}+\mathrm{fLP})^2\right)\Bigg/$$

$$\left(\frac{4\ \mathrm{fHP}\ \mathrm{fHR}\ \mathrm{fLP}\ \mathrm{fLR}\ (p-p\,\tau)\ (1-p-\tau+p\,\tau)\ \varphi\mathrm{P}\ \varphi\mathrm{R}}{(\mathrm{fHP}+\mathrm{fLP})^2\ (\mathrm{fHR}+\mathrm{fLR})^2}-\left(1-\frac{2\ \mathrm{fHP}\ \mathrm{fLP}\ (p+\tau-p\,\tau)\ \varphi\mathrm{P}}{(\mathrm{fHP}+\mathrm{fLP})^2}\right)\left(1-\frac{2\ \mathrm{fHR}\ \mathrm{fLR}\ (1-p+p\,\tau)\ \varphi\mathrm{R}}{(\mathrm{fHR}+\mathrm{fLR})^2}\right)\right)\Bigg)\Bigg)$$

```
rP[τ_] :=
 -((fHP^2 fHR^2 p φP + fHR^2 fLP^2 p φP + 2 fHP^2 fHR fLR p φP + 2 fHR fLP^2 fLR p φP +
      fHP^2 fLR^2 p φP + fLP^2 fLR^2 p φP + fHP^2 fHR^2 τ φP + fHR^2 fLP^2 τ φP +
      2 fHP^2 fHR fLR τ φP + 2 fHR fLP^2 fLR τ φP + fHP^2 fLR^2 τ φP +
      fLP^2 fLR^2 τ φP - fHP^2 fHR^2 p τ φP - fHR^2 fLP^2 p τ φP - 2 fHP^2 fHR fLR p τ φP -
      2 fHR fLP^2 fLR p τ φP - fHP^2 fLR^2 p τ φP - fLP^2 fLR^2 p τ φP +
      fHP^2 fHR^2 φR + 2 fHP fHR^2 fLP φR + fHR^2 fLP^2 φR + fHP^2 fLR^2 φR +
      2 fHP fLP fLR^2 φR + fLP^2 fLR^2 φR - fHP^2 fHR^2 p φR - 2 fHP fHR^2 fLP p φR -
      fHR^2 fLP^2 p φR - fHP^2 fLR^2 p φR - 2 fHP fLP fLR^2 p φR - fLP^2 fLR^2 p φR -
      fHP^2 fHR^2 τ φR - 2 fHP fHR^2 fLP τ φR - fHR^2 fLP^2 τ φR - fHP^2 fLR^2 τ φR -
      2 fHP fLP fLR^2 τ φR - fLP^2 fLR^2 τ φR + fHP^2 fHR^2 p τ φR +
      2 fHP fHR^2 fLP p τ φR + fHR^2 fLP^2 p τ φR + fHP^2 fLR^2 p τ φR +
      2 fHP fLP fLR^2 p τ φR + fLP^2 fLR^2 p τ φR - 2 fHP^2 fHR fLR τ φP φR -
      2 fHR fLP^2 fLR τ φP φR) /
    (-fHP^2 fHR^2 - 2 fHP fHR^2 fLP - fHR^2 fLP^2 - 2 fHP^2 fHR fLR -
      4 fHP fHR fLP fLR - 2 fHR fLP^2 fLR - fHP^2 fLR^2 - 2 fHP fLP fLR^2 -
      fLP^2 fLR^2 + 2 fHP fHR^2 fLP p φP + 4 fHP fHR fLP fLR p φP +
      2 fHP fLP fLR^2 p φP + 2 fHP fHR^2 fLP τ φP + 4 fHP fHR fLP fLR τ φP +
      2 fHP fLP fLR^2 τ φP - 2 fHP fHR^2 fLP p τ φP - 4 fHP fHR fLP fLR p τ φP -
      2 fHP fLP fLR^2 p τ φP + 2 fHP^2 fHR fLR φR + 4 fHP fHR fLP fLR φR +
      2 fHR fLP^2 fLR φR - 2 fHP^2 fHR fLR p φR - 4 fHP fHR fLP fLR p φR -
      2 fHR fLP^2 fLR p φR + 2 fHP^2 fHR fLR p τ φR + 4 fHP fHR fLP fLR p τ φR +
      2 fHR fLP^2 fLR p τ φR - 4 fHP fHR fLP fLR τ φP φR))
```

```
Plot[{rR[τ] /. {xHR → 1, xLR → 1, xHP → 1, xLP → 1}}, {τ, -1, 1},
 PlotRange → {{-1, 1}, {0, 1}}, Frame → True, AxesOrigin → {-1, -0.0},
```

```
 FrameLabel → {{Text[Style["relatedness, rR"]], None}, {None, None}},
 FrameTicks → {{vlabel, None}, {hlabel, None}}, AspectRatio → 1,
 ImageSize → 200]
Plot[{rP[τ] /. {xHR → 1, xLR → 1, xHP → 1, xLP → 1}}, {τ, -1, 1},
 PlotRange → {{-1, 1}, {0, 1}}, Frame → True, AxesOrigin → {-1, -0.0},
 FrameLabel → {{Text[Style["relatedness, rP"]], None}, {None, None}},
 FrameTicks → {{vlabel, None}, {hlabel, None}}, AspectRatio → 1,
 ImageSize → 200]
```

Out[8828]=

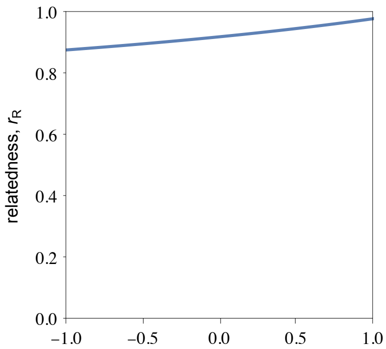

Out[8829]=

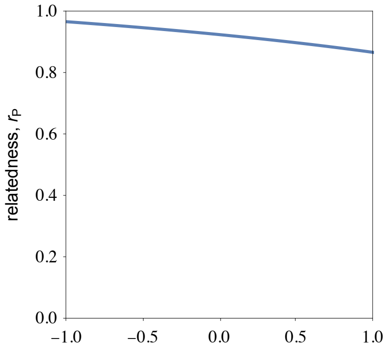

Reproductive value

calculations

```
wHRHR[τ_] :=
  ((1 - d) / ((1 + sR) (1 - d) + (p (1 + sR) + (1 - p) σ (1 + sP)) d (1 - c)))
    (p + τ - p τ) +
   ((d (1 - c) p) / ((1 + sR) (1 - d) + (p (1 + sR) + (1 - p) σ (1 + sP)) d (1 - c)))
    (p + τ - p τ) +
   ((d (1 - c) (1 - p)) /
      (σ (1 + sP) (1 - d) + (p (1 + sR) + (1 - p) σ (1 + sP)) d (1 - c)))
    (1 - (1 - p + p τ));
wHRLR[τ_] :=
  ((1 - d) / ((1 + sR) (1 - d) + (p (1 + sR) + (1 - p) σ (1 + sP)) d (1 - c)))
    (p + τ - p τ) +
   ((d (1 - c) p) / ((1 + sR) (1 - d) + (p (1 + sR) + (1 - p) σ (1 + sP)) d (1 - c)))
    (p + τ - p τ) +
```

```
    ((d (1 - c) (1 - p)) /
       (σ (1 + sP) (1 - d) + (p (1 + sR) + (1 - p) σ (1 + sP)) d (1 - c)))
     (1 - (1 - p + p τ));


wHRHP[τ_] :=
  ((1 - d) / ((1 + sR) (1 - d) + (p (1 + sR) + (1 - p) σ (1 + sP)) d (1 - c)))
     (1 - (p + τ - p τ)) +
    ((d (1 - c) p) / ((1 + sR) (1 - d) + (p (1 + sR) + (1 - p) σ (1 + sP)) d (1 - c)))
     (1 - (p + τ - p τ)) +
    ((d (1 - c) (1 - p)) /
       (σ (1 + sP) (1 - d) + (p (1 + sR) + (1 - p) σ (1 + sP)) d (1 - c))) (1 - p + p τ);
wHRLP[τ_] :=
  ((1 - d) / ((1 + sR) (1 - d) + (p (1 + sR) + (1 - p) σ (1 + sP)) d (1 - c)))
     (1 - (p + τ - p τ)) +
    ((d (1 - c) p) / ((1 + sR) (1 - d) + (p (1 + sR) + (1 - p) σ (1 + sP)) d (1 - c)))
     (1 - (p + τ - p τ)) +
    ((d (1 - c) (1 - p)) /
       (σ (1 + sP) (1 - d) + (p (1 + sR) + (1 - p) σ (1 + sP)) d (1 - c))) (1 - p + p τ);


wHPHP[τ_] :=
  ((σ (1 - d)) / (σ (1 + sP) (1 - d) + (p (1 + sR) + (1 - p) σ (1 + sP)) d (1 - c)))
     (1 - p + p τ) +
    ((σ d (1 - c) p) / ((1 + sR) (1 - d) + (p (1 + sR) + (1 - p) σ (1 + sP)) d (1 - c)))
     (1 - (p + τ - p τ)) +
    ((σ d (1 - c) (1 - p)) /
       (σ (1 + sP) (1 - d) + (p (1 + sR) + (1 - p) σ (1 + sP)) d (1 - c))) (1 - p + p τ);
wHPLP[τ_] :=
  ((σ (1 - d)) / (σ (1 + sP) (1 - d) + (p (1 + sR) + (1 - p) σ (1 + sP)) d (1 - c)))
     (1 - p + p τ) +
    ((σ d (1 - c) p) / ((1 + sR) (1 - d) + (p (1 + sR) + (1 - p) σ (1 + sP)) d (1 - c)))
     (1 - (p + τ - p τ)) +
    ((σ d (1 - c) (1 - p)) /
       (σ (1 + sP) (1 - d) + (p (1 + sR) + (1 - p) σ (1 + sP)) d (1 - c))) (1 - p + p τ);


wHPHR[τ_] :=
  ((σ (1 - d)) / (σ (1 + sP) (1 - d) + (p (1 + sR) + (1 - p) σ (1 + sP)) d (1 - c)))
     (1 - (1 - p + p τ)) +
    ((σ d (1 - c) p) / ( (1 + sR) (1 - d) + (p  (1 + sR) + (1 - p) σ  (1 + sP)) d (1 - c)))
     (p + τ - p τ) +
    ((σ d (1 - c) (1 - p)) /
```

```
      (σ (1 + sP) (1 - d) + (p (1 + sR) + (1 - p) σ (1 + sP)) d (1 - c)))
    (1 - (1 - p + p τ));
wHPLR[τ_] :=
  ((σ (1 - d)) / (σ (1 + sP) (1 - d) + (p (1 + sR) + (1 - p) σ (1 + sP)) d (1 - c)))
    (1 - (1 - p + p τ)) +
   ((σ d (1 - c) p) / ( (1 + sR) (1 - d) + (p  (1 + sR) + (1 - p) σ  (1 + sP)) d (1 - c)))
    (p + τ - p τ) +
   ((σ d (1 - c) (1 - p)) /
      (σ (1 + sP) (1 - d) + (p (1 + sR) + (1 - p) σ (1 + sP)) d (1 - c)))
    (1 - (1 - p + p τ));


wLRLR[τ_] :=
  ((sR (1 - d)) / ( (1 + sR) (1 - d) + (p (1 + sR) + (1 - p) σ (1 + sP)) d (1 - c)))
    (p + τ - p τ) +
   ((sR d (1 - c) p) / ( (1 + sR) (1 - d) + (p (1 + sR) + (1 - p) σ (1 + sP)) d (1 - c)))
    (p + τ - p τ) +
   ((sR d (1 - c) (1 - p)) /
      (σ (1 + sP) (1 - d) + (p (1 + sR) + (1 - p) σ (1 + sP)) d (1 - c)))
    (1 - (1 - p + p τ));
wLRHR[τ_] :=
  ((sR (1 - d)) / ( (1 + sR) (1 - d) + (p (1 + sR) + (1 - p) σ (1 + sP)) d (1 - c)))
    (p + τ - p τ) +
   ((sR d (1 - c) p) / ( (1 + sR) (1 - d) + (p (1 + sR) + (1 - p) σ (1 + sP)) d (1 - c)))
    (p + τ - p τ) +
   ((sR d (1 - c) (1 - p)) /
      (σ (1 + sP) (1 - d) + (p (1 + sR) + (1 - p) σ (1 + sP)) d (1 - c)))
    (1 - (1 - p + p τ));


wLRLP[τ_] :=
  ((sR (1 - d)) / ((1 + sR) (1 - d) + (p (1 + sR) + (1 - p) σ (1 + sP)) d (1 - c)))
    (1 - (p + τ - p τ)) +
   ((sR d (1 - c) p) / ((1 + sR) (1 - d) + (p (1 + sR) + (1 - p) σ (1 + sP)) d (1 - c)))
    (1 - (p + τ - p τ)) +
   ((sR d (1 - c) (1 - p)) /
      (σ (1 + sP) (1 - d) + (p (1 + sR) + (1 - p) σ (1 + sP)) d (1 - c))) (1 - p + p τ);
wLRHP[τ_] :=
  ((sR (1 - d)) / ((1 + sR) (1 - d) + (p (1 + sR) + (1 - p) σ (1 + sP)) d (1 - c)))
    (1 - (p + τ - p τ)) +
   ((sR d (1 - c) p) / ((1 + sR) (1 - d) + (p (1 + sR) + (1 - p) σ (1 + sP)) d (1 - c)))
    (1 - (p + τ - p τ)) +
   ((sR d (1 - c) (1 - p)) /
```

```
        (σ (1 + sP) (1 - d) + (p (1 + sR) + (1 - p) σ (1 + sP)) d (1 - c))) (1 - p + p τ);


wLPLP[τ_] :=
  ((σ sP (1 - d)) / (σ (1 + sP) (1 - d) + (p (1 + sR) + (1 - p) σ (1 + sP)) d (1 - c)))
    (1 - p + p τ) +
   ((σ sP d (1 - c) p) /
      ((1 + sR) (1 - d) + (p (1 + sR) + (1 - p) σ (1 + sP)) d (1 - c)))
    (1 - (p + τ - p τ)) +
   ((σ sP d (1 - c) (1 - p)) /
      (σ (1 + sP) (1 - d) + (p (1 + sR) + (1 - p) σ (1 + sP)) d (1 - c))) (1 - p + p τ);
wLPHP[τ_] :=
  ((σ sP (1 - d)) / (σ (1 + sP) (1 - d) + (p (1 + sR) + (1 - p) σ (1 + sP)) d (1 - c)))
    (1 - p + p τ) +
   ((σ sP d (1 - c) p) /
      ((1 + sR) (1 - d) + (p (1 + sR) + (1 - p) σ (1 + sP)) d (1 - c)))
    (1 - (p + τ - p τ)) +
   ((σ sP d (1 - c) (1 - p)) /
      (σ (1 + sP) (1 - d) + (p (1 + sR) + (1 - p) σ (1 + sP)) d (1 - c))) (1 - p + p τ);


wLPLR[τ_] :=
  ((σ sP (1 - d)) / (σ (1 + sP) (1 - d) + (p (1 + sR) + (1 - p) σ (1 + sP)) d (1 - c)))
    (1 - (1 - p + p τ)) +
   ((σ sP d (1 - c) p) /
      ( (1 + sR) (1 - d) + (p (1 + sR) + (1 - p) σ (1 + sP)) d (1 - c))) (p + τ - p τ) +
   ((σ sP d (1 - c) (1 - p)) /
      (σ (1 + sP) (1 - d) + (p (1 + sR) + (1 - p) σ (1 + sP)) d (1 - c)))
    (1 - (1 - p + p τ));
wLPHR[τ_] :=
  ((σ sP (1 - d)) / (σ (1 + sP) (1 - d) + (p (1 + sR) + (1 - p) σ (1 + sP)) d (1 - c)))
    (1 - (1 - p + p τ)) +
   ((σ sP d (1 - c) p) /
      ( (1 + sR) (1 - d) + (p (1 + sR) + (1 - p) σ (1 + sP)) d (1 - c))) (p + τ - p τ) +
   ((σ sP d (1 - c) (1 - p)) /
      (σ (1 + sP) (1 - d) + (p (1 + sR) + (1 - p) σ (1 + sP)) d (1 - c)))
    (1 - (1 - p + p τ));
```

$$
A[\tau\_] := \begin{pmatrix} wHRHR[\tau] & wLRHR[\tau] & wHPHR[\tau] & wLPHR[\tau] \\ wHRLR[\tau] & wLRLR[\tau] & wHPLR[\tau] & wLPLR[\tau] \\ wHRHP[\tau] & wLRHP[\tau] & wHPHP[\tau] & wLPHP[\tau] \\ wHRLP[\tau] & wLRLP[\tau] & wHPLP[\tau] & wLPLP[\tau] \end{pmatrix};
$$

```
AP = A[τ];

Eigenvalues[AP] // Simplify
Eigensystem[AP] // Simplify
Eigensystem[Transpose[AP]] // Simplify
```

```
Out[9578]= {0, 0, 1, (4 (-1 + d) (-1 + c d) (1 + sP) (1 + sR) σ τ) /
    ((-2 (1 + sR) + d (1 + sR - σ - sP σ + c (1 + sR + σ + sP σ)))
      (-2 (1 + sP) σ + d (-1 - sR + σ + sP σ + c (1 + sR + σ + sP σ))))}

Out[9579]= {{0, 0, 1, (4 (-1 + d) (-1 + c d) (1 + sP) (1 + sR) σ τ) /
    ((-2 (1 + sR) + d (1 + sR - σ - sP σ + c (1 + sR + σ + sP σ)))
      (-2 (1 + sP) σ + d (-1 - sR + σ + sP σ + c (1 + sR + σ + sP σ))))},
  {{0, 0, -sP, 1}, {-sR, 1, 0, 0}, {1, 1, 1, 1},
   {-(((1 + sP) σ (-2 (1 + sR) (-1 + τ) + d^2 (1 + sR - σ - sP σ + c^2 (1 + sR + σ + sP σ) - 2 c (1 + sR)
          τ) + 2 d (-1 + σ + sP σ + sR (-1 + τ) + τ - c (1 + sR + σ + sP σ - τ - sR τ)))) /
      ((1 + sR) (-2 (1 + sP) σ (-1 + τ) - 2 d (-1 - sR + σ + sP σ + c (1 + sR - (1 + sP) σ (-1 + τ)) -
          σ τ - sP σ τ) + d^2 (-1 - sR + σ + sP σ + c^2 (1 + sR + σ + sP σ) - 2 c (1 + sP) σ τ)))),
    -(((1 + sP) σ (-2 (1 + sR) (-1 + τ) + d^2 (1 + sR - σ - sP σ + c^2 (1 + sR + σ + sP σ) - 2 c (1 + sR)
          τ) + 2 d (-1 + σ + sP σ + sR (-1 + τ) + τ - c (1 + sR + σ + sP σ - τ - sR τ)))) /
      ((1 + sR) (-2 (1 + sP) σ (-1 + τ) - 2 d (-1 - sR + σ + sP σ +
          c (1 + sR - (1 + sP) σ (-1 + τ)) - σ τ - sP σ τ) +
        d^2 (-1 - sR + σ + sP σ + c^2 (1 + sR + σ + sP σ) - 2 c (1 + sP) σ τ)))), 1, 1}}}

Out[9580]= {{0, 0, 1, (4 (-1 + d) (-1 + c d) (1 + sP) (1 + sR) σ τ) /
    ((-2 (1 + sR) + d (1 + sR - σ - sP σ + c (1 + sR + σ + sP σ)))
      (-2 (1 + sP) σ + d (-1 - sR + σ + sP σ + c (1 + sR + σ + sP σ))))},
  {{0, 0, -1, 1}, {-1, 1, 0, 0}, {(-2 (1 + sP) σ (-1 + τ) -
       2 d (-1 - sR + σ + sP σ + c (1 + sR - (1 + sP) σ (-1 + τ)) - σ τ - sP σ τ) +
       d^2 (-1 - sR + σ + sP σ + c^2 (1 + sR + σ + sP σ) - 2 c (1 + sP) σ τ)) /
     (sP σ (-2 (1 + sR) (-1 + τ) + d^2 (1 + sR - σ - sP σ + c^2 (1 + sR + σ + sP σ) - 2 c (1 + sR) τ) +
        2 d (-1 + σ + sP σ + sR (-1 + τ) + τ - c (1 + sR + σ + sP σ - τ - sR τ)))),
    (sR (-2 (1 + sP) σ (-1 + τ) - 2 d (-1 - sR + σ + sP σ + c (1 + sR - (1 + sP) σ (-1 + τ)) -
          σ τ - sP σ τ) + d^2 (-1 - sR + σ + sP σ + c^2 (1 + sR + σ + sP σ) - 2 c (1 + sP) σ τ))) /
     (sP σ (-2 (1 + sR) (-1 + τ) + d^2 (1 + sR - σ - sP σ + c^2 (1 + sR + σ + sP σ) - 2 c (1 + sR) τ) +
        2 d (-1 + σ + sP σ + sR (-1 + τ) + τ - c (1 + sR + σ + sP σ - τ - sR τ)))),
    1/sP, 1}, {-(1 + sP)/(sP + sP sR), -(sR + sP sR)/(sP + sP sR), 1/sP, 1}}}
```

Plot

```
c = 0.9;
d = 0.1;
μR = 1;
μP = 0.1;
qHR = 1;
```

```
qLR = 0.1;
qHP = 1;
qLP = 0.1;
```

$$fHR := \mu R \left(qHR + \frac{xHR}{\frac{1}{2}(xHR + xLR)} \left(1 - \frac{1}{2}(xHR + xLR)\right)\right);$$

$$fLR := \mu R \left(qLR + \frac{xLR}{\frac{1}{2}(xHR + xLR)} \left(1 - \frac{1}{2}(xHR + xLR)\right)\right);$$

$$fHP := \mu P \left(qHP + \frac{xHP}{\frac{1}{2}(xHP + xLP)} \left(1 - \frac{1}{2}(xHP + xLP)\right)\right);$$

$$fLP := \mu P \left(qLP + \frac{xLP}{\frac{1}{2}(xHP + xLP)} \left(1 - \frac{1}{2}(xHP + xLP)\right)\right);$$

$$sH = \frac{fLR}{fHR};\ sL = \frac{fLP}{fHP};\ \sigma = \frac{fHP}{fHR};$$

```
vHR[τ_] := 1
vLR[τ_] :=
  (sH
      (-2 (1 + sL) σ (-1 + τ) -
        2 d (-1 - sH + σ + sL σ + c (1 + sH - (1 + sL) σ (-1 + τ)) - σ τ - sL σ τ) +
        d^2 (-1 - sH + σ + sL σ + c^2 (1 + sH + σ + sL σ) - 2 c (1 + sL) σ τ))) /
    (sL σ
      (-2 (1 + sH) (-1 + τ) +
        d^2 (1 + sH - σ - sL σ + c^2 (1 + sH + σ + sL σ) - 2 c (1 + sH) τ) +
        2 d (-1 + σ + sL σ + sH (-1 + τ) + τ - c (1 + sH + σ + sL σ - τ - sH τ)))) /
   (-2 (1 + sL) σ (-1 + τ) -
      2 d (-1 - sH + σ + sL σ + c (1 + sH - (1 + sL) σ (-1 + τ)) - σ τ - sL σ τ) +
      d^2 (-1 - sH + σ + sL σ + c^2 (1 + sH + σ + sL σ) - 2 c (1 + sL) σ τ)) /
    (sL σ
      (-2 (1 + sH) (-1 + τ) +
        d^2 (1 + sH - σ - sL σ + c^2 (1 + sH + σ + sL σ) - 2 c (1 + sH) τ) +
        2 d (-1 + σ + sL σ + sH (-1 + τ) + τ - c (1 + sH + σ + sL σ - τ - sH τ))))
vHP[τ_] :=
  1/sL /
   (-2 (1 + sL) σ (-1 + τ) -
      2 d (-1 - sH + σ + sL σ + c (1 + sH - (1 + sL) σ (-1 + τ)) - σ τ - sL σ τ) +
```

```
      d^2 (-1 - sH + σ + sL σ + c^2 (1 + sH + σ + sL σ) - 2 c (1 + sL) σ τ)) /
    (sL σ
      (-2 (1 + sH) (-1 + τ) +
        d^2 (1 + sH - σ - sL σ + c^2 (1 + sH + σ + sL σ) - 2 c (1 + sH) τ) +
        2 d (-1 + σ + sL σ + sH (-1 + τ) + τ - c (1 + sH + σ + sL σ - τ - sH τ))))
vLP[τ_] :=
 1/
  (-2 (1 + sL) σ (-1 + τ) -
      2 d (-1 - sH + σ + sL σ + c (1 + sH - (1 + sL) σ (-1 + τ)) - σ τ - sL σ τ) +
      d^2 (-1 - sH + σ + sL σ + c^2 (1 + sH + σ + sL σ) - 2 c (1 + sL) σ τ)) /
    (sL σ
      (-2 (1 + sH) (-1 + τ) +
        d^2 (1 + sH - σ - sL σ + c^2 (1 + sH + σ + sL σ) - 2 c (1 + sH) τ) +
        2 d (-1 + σ + sL σ + sH (-1 + τ) + τ - c (1 + sH + σ + sL σ - τ - sH τ))))

wHRϕ[τ_] :=
  ((1 - d) (fHR)) /
   ((1 - d) (fHR + fLR) + (p (fHR + fLR) + (1 - p) (fHP + fLP)) d (1 - c))
wLRϕ[τ_] :=
  ((1 - d) (fLR)) /
   ((1 - d) (fHR + fLR) + (p (fHR + fLR) + (1 - p) (fHP + fLP)) d (1 - c))
wHPϕ[τ_] :=
  ((1 - d) (fHP)) /
   ((1 - d) (fHP + fLP) + (p (fHR + fLR) + (1 - p) (fHP + fLP)) d (1 - c))
wLPϕ[τ_] :=
  ((1 - d) (fLP)) /
   ((1 - d) (fHP + fLP) + (p (fHR + fLR) + (1 - p) (fHP + fLP)) d (1 - c))

vHRϕ[τ_] :=
 wHRϕ[τ] ((1 - p + p τ) (vHR[τ] + vLR[τ]) + (1 - (1 - p + p  τ)) (vHP[τ] + vLP[τ]))
vLRϕ[τ_] :=
 wLRϕ[τ] ((1 - p + p τ) (vHR[τ] + vLR[τ]) + (1 - (1 - p + p τ)) (vHP[τ] + vLP[τ]))
vHPϕ[τ_] :=
 wHPϕ[τ] ((1 - (p + τ - p τ)) (vHR[τ] + vLR[τ]) + (p + τ - p τ) (vHP[τ] + vLP[τ]))
vLPϕ[τ_] :=
 wLPϕ[τ] ((1 - (p + τ - p τ)) (vHR[τ] + vLR[τ]) + (p + τ - p τ) (vHP[τ] + vLP[τ]))

Plot[{vHRϕ[τ] /. {xHR → 1, xLR → 1, xHP → 1, xLP → 1},
   vLRϕ[τ] /. {xHR → 1, xLR → 1, xHP → 1, xLP → 1}}, {τ, -1, 1},
  PlotRange → {{-1, 1}, {0, 1}}, Frame → True, AxesOrigin → {-1, -0.0},
  FrameLabel → {{Text[Style["reproductive value, v^ϕ"]], None},
```

```
    {None, None}}, FrameTicks → {{vlabel, None}, {hlabel, None}},
  PlotStyle → style1, AspectRatio → 1, ImageSize → 200]
Plot[{vHPϕ[τ] /. {xHR → 1, xLR → 1, xHP → 1, xLP → 1},
   vLPϕ[τ] /. {xHR → 1, xLR → 1, xHP → 1, xLP → 1}}, {τ, -1, 1},
  PlotRange → {{-1, 1}, {0, 1}}, Frame → True, AxesOrigin → {-1, -0.0},
  FrameLabel → {{Text[Style["reproductive value, vϕ"]], None},
    {None, None}}, FrameTicks → {{vlabel, None}, {hlabel, None}},
  PlotStyle → style1, AspectRatio → 1, ImageSize → 200]
```

Out[8848]=

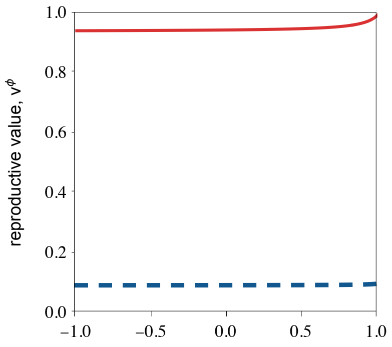

Out[8849]=

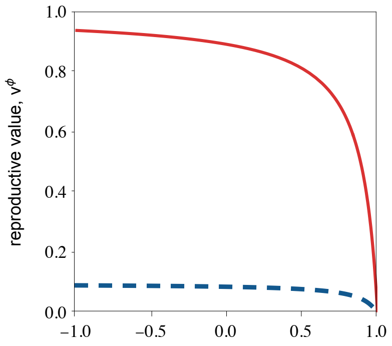

Potential for helping

```
ωR[τ_] :=
  (1 - d) / ((1 - d) (fHR + fLR) + (p (fHR + fLR) + (1 - p) (fHP + fLP)) d (1 - c))

ωP[τ_] :=
  (1 - d) / ((1 - d) (fHP + fLP) + (p (fHR + fLR) + (1 - p) (fHP + fLP)) d (1 - c))
```

$$\text{AHR}[\tau\_] := \frac{\frac{\text{vLR}[\tau]}{\text{fLR}}\,\text{rR}[\tau] - \omega\text{R}[\tau]\ (\text{vHR}\phi[\tau] + \text{vLR}\phi[\tau]\ \text{rR}[\tau])}{\frac{\text{vHR}[\tau]}{\text{fHR}} - \omega\text{R}[\tau]\ (\text{vHR}\phi[\tau] + \text{vLR}\phi[\tau]\ \text{rR}[\tau])};$$

$$\text{ALR}[\tau\_] := \frac{\frac{\text{vHR}[\tau]}{\text{fHR}}\,\text{rR}[\tau] - \omega\text{R}[\tau]\ (\text{vHR}\phi[\tau]\ \text{rR}[\tau] + \text{vLR}\phi[\tau])}{\frac{\text{vLR}[\tau]}{\text{fLR}} - \omega\text{R}[\tau]\ (\text{vHR}\phi[\tau]\ \text{rR}[\tau] + \text{vLR}\phi[\tau])};$$

$$\text{AHP}[\tau\_] := \frac{\frac{\text{vLP}[\tau]}{\text{fLP}}\,\text{rP}[\tau] - \omega\text{P}[\tau]\ (\text{vHP}\phi[\tau] + \text{vLP}\phi[\tau]\ \text{rP}[\tau])}{\frac{\text{vHP}[\tau]}{\text{fHP}} - \omega\text{P}[\tau]\ (\text{vHP}\phi[\tau] + \text{vLP}\phi[\tau]\ \text{rP}[\tau])};$$

$$\text{ALP}[\tau\_] := \frac{\frac{\text{vHP}[\tau]}{\text{fHP}}\,\text{rP}[\tau] - \omega\text{P}[\tau]\ (\text{vHP}\phi[\tau]\ \text{rP}[\tau] + \text{vLP}\phi[\tau])}{\frac{\text{vLP}[\tau]}{\text{fLP}} - \omega\text{P}[\tau]\ (\text{vHP}\phi[\tau]\ \text{rP}[\tau] + \text{vLP}\phi[\tau])};$$

```
Plot[{AHR[τ] /. {xHR → 1, xLR → 1, xHP → 1, xLP → 1},
   ALR[τ] /. {xHR → 1, xLR → 1, xHP → 1, xLP → 1}}, {τ, -1, 1},
  PlotRange → {{-1, 1}, {-0.81, 0.81}}, Frame → True,
  FrameLabel → {{Text[Style["potential for helping, A"]], None},
    {None, None}}, AxesOrigin → {-1, -0.0},
  FrameTicks → {{pticks, None}, {hlabel, None}}, PlotStyle → style1,
  AspectRatio → 1, ImageSize → 200]
Plot[{AHP[τ] /. {xHR → 1, xLR → 1, xHP → 1, xLP → 1},
   ALP[τ] /. {xHR → 1, xLR → 1, xHP → 1, xLP → 1}}, {τ, -1, 1},
  PlotRange → {{-1, 1}, {-0.81, 0.81}}, Frame → True,
  FrameTicks → {{pticks, None}, {hlabel, None}},
  FrameLabel → {{Text[Style["potential for helping, A"]], None},
    {None, None}}, AxesOrigin → {-1, -0.0}, PlotStyle → style1,
  AspectRatio → 1, ImageSize → 200]
```

Out[8873]=

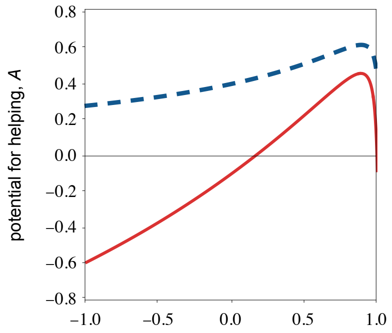

Out[8874]=

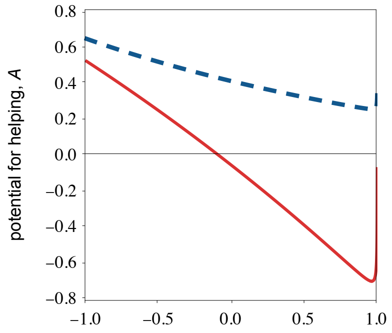

Optimal behaviour

```
c = 0.9;
d = 0.1;
μR = 1;
μP = 0.1;
qHR = 1;
qLR = 0.1;
qHP = 1;
qLP = 0.1;
```

$$fHR := \mu R \left(qHR + \frac{xHR}{\frac{1}{2}\,(xHR + xLR)} \left(1 - \frac{1}{2}\,(xHR + xLR)\right)\right);$$

$$fLR := \mu R \left(qLR + \frac{xLR}{\frac{1}{2}\,(xHR + xLR)} \left(1 - \frac{1}{2}\,(xHR + xLR)\right)\right);$$

$$fHP := \mu P \left(qHP + \frac{xHP}{\frac{1}{2}\,(xHP + xLP)} \left(1 - \frac{1}{2}\,(xHP + xLP)\right)\right);$$

$$fLP := \mu P \left(qLP + \frac{xLP}{\frac{1}{2}\,(xHP + xLP)} \left(1 - \frac{1}{2}\,(xHP + xLP)\right)\right);$$

$$sR = \frac{fLR}{fHR};\; sP = \frac{fLP}{fHP};\; \sigma = \frac{fHP}{fHR};$$

$$\text{cHR} := -\frac{1}{\frac{1}{2}}\left(1-\frac{1}{2}(\text{xHR}+\text{xLR})\right);$$

$$\text{bHR} := -\frac{\text{xHR}}{\frac{1}{2}(\text{xHR}+\text{xLR})};$$

$$\text{cLR} := -\frac{1}{\frac{1}{2}}\left(1-\frac{1}{2}(\text{xHR}+\text{xLR})\right);$$

$$\text{bLR} := -\frac{\text{xLR}}{\frac{1}{2}(\text{xHR}+\text{xLR})};$$

$$\text{cHP} := -\frac{1}{\frac{1}{2}}\left(1-\frac{1}{2}(\text{xHP}+\text{xLP})\right);$$

$$\text{bHP} := -\frac{\text{xHP}}{\frac{1}{2}(\text{xHP}+\text{xLP})};$$

$$\text{cLP} := -\frac{1}{\frac{1}{2}}\left(1-\frac{1}{2}(\text{xHP}+\text{xLP})\right);$$

$$\text{bLP} := -\frac{\text{xLP}}{\frac{1}{2}(\text{xHP}+\text{xLP})};$$

$$\text{vHR}[\tau\_] := 1$$

$$\begin{aligned}
&\text{vLR}[\tau_] := \\
&\quad \Big(\text{sR} \\
&\qquad \Big(-2\,(1+\text{sP})\,\sigma\,(-1+\tau) - \\
&\qquad\quad 2\,\text{d}\,(-1-\text{sR}+\sigma+\text{sP}\,\sigma+\text{c}\,(1+\text{sR}-(1+\text{sP})\,\sigma\,(-1+\tau))-\sigma\,\tau-\text{sP}\,\sigma\,\tau) + \\
&\qquad\quad \text{d}^2\,\Big(-1-\text{sR}+\sigma+\text{sP}\,\sigma+\text{c}^2\,(1+\text{sR}+\sigma+\text{sP}\,\sigma)-2\,\text{c}\,(1+\text{sP})\,\sigma\,\tau\Big)\Big)\Big) \Big/ \\
&\quad\ \Big(\text{sP}\,\sigma \\
&\qquad \Big(-2\,(1+\text{sR})\,(-1+\tau) + \\
&\qquad\quad \text{d}^2\,\Big(1+\text{sR}-\sigma-\text{sP}\,\sigma+\text{c}^2\,(1+\text{sR}+\sigma+\text{sP}\,\sigma)-2\,\text{c}\,(1+\text{sR})\,\tau\Big) + \\
&\qquad\quad 2\,\text{d}\,(-1+\sigma+\text{sP}\,\sigma+\text{sR}\,(-1+\tau)+\tau-\text{c}\,(1+\text{sR}+\sigma+\text{sP}\,\sigma-\tau-\text{sR}\,\tau))\Big)\Big) \Big/ \\
&\quad \Big(-2\,(1+\text{sP})\,\sigma\,(-1+\tau) - \\
&\qquad 2\,\text{d}\,(-1-\text{sR}+\sigma+\text{sP}\,\sigma+\text{c}\,(1+\text{sR}-(1+\text{sP})\,\sigma\,(-1+\tau))-\sigma\,\tau-\text{sP}\,\sigma\,\tau) + \\
&\qquad \text{d}^2\,\Big(-1-\text{sR}+\sigma+\text{sP}\,\sigma+\text{c}^2\,(1+\text{sR}+\sigma+\text{sP}\,\sigma)-2\,\text{c}\,(1+\text{sP})\,\sigma\,\tau\Big)\Big) \Big/ \\
&\quad\ \Big(\text{sP}\,\sigma \\
&\qquad \Big(-2\,(1+\text{sR})\,(-1+\tau) +
\end{aligned}$$

```
          d^2 (1 + sR - σ - sP σ + c^2 (1 + sR + σ + sP σ) - 2 c (1 + sR) τ) +
          2 d (-1 + σ + sP σ + sR (-1 + τ) + τ - c (1 + sR + σ + sP σ - τ - sR τ))))
vHP[τ_] :=
  1/sP /
   (-2 (1 + sP) σ (-1 + τ) -
       2 d (-1 - sR + σ + sP σ + c (1 + sR - (1 + sP) σ (-1 + τ)) - σ τ - sP σ τ) +
       d^2 (-1 - sR + σ + sP σ + c^2 (1 + sR + σ + sP σ) - 2 c (1 + sP) σ τ)) /
    (sP σ
      (-2 (1 + sR) (-1 + τ) +
        d^2 (1 + sR - σ - sP σ + c^2 (1 + sR + σ + sP σ) - 2 c (1 + sR) τ) +
        2 d (-1 + σ + sP σ + sR (-1 + τ) + τ - c (1 + sR + σ + sP σ - τ - sR τ))))
vLP[τ_] :=
  1/
   (-2 (1 + sP) σ (-1 + τ) -
       2 d (-1 - sR + σ + sP σ + c (1 + sR - (1 + sP) σ (-1 + τ)) - σ τ - sP σ τ) +
       d^2 (-1 - sR + σ + sP σ + c^2 (1 + sR + σ + sP σ) - 2 c (1 + sP) σ τ)) /
    (sP σ
      (-2 (1 + sR) (-1 + τ) +
        d^2 (1 + sR - σ - sP σ + c^2 (1 + sR + σ + sP σ) - 2 c (1 + sR) τ) +
        2 d (-1 + σ + sP σ + sR (-1 + τ) + τ - c (1 + sR + σ + sP σ - τ - sR τ))))

wHRϕ[τ_] :=
  ((1 - d) (fHR)) /
   ((1 - d) (fHR + fLR) + (p (fHR + fLR) + (1 - p) (fHP + fLP)) d (1 - c))
wLRϕ[τ_] :=
  ((1 - d) (fLR)) /
   ((1 - d) (fHR + fLR) + (p (fHR + fLR) + (1 - p) (fHP + fLP)) d (1 - c))
wHPϕ[τ_] :=
  ((1 - d) (fHP)) /
   ((1 - d) (fHP + fLP) + (p (fHR + fLR) + (1 - p) (fHP + fLP)) d (1 - c))
wLPϕ[τ_] :=
  ((1 - d) (fLP)) /
   ((1 - d) (fHP + fLP) + (p (fHR + fLR) + (1 - p) (fHP + fLP)) d (1 - c))

vHRϕ[τ_] :=
  wHRϕ[τ] ((1 - p + p τ) (vHR[τ] + vLR[τ]) + (1 - (1 - p + p  τ)) (vHP[τ] + vLP[τ]))
vLRϕ[τ_] :=
  wLRϕ[τ] ((1 - p + p τ) (vHR[τ] + vLR[τ]) + (1 - (1 - p + p τ)) (vHP[τ] + vLP[τ]))
vHPϕ[τ_] :=
  wHPϕ[τ] ((1 - (p + τ - p τ)) (vHR[τ] + vLR[τ]) + (p + τ - p τ) (vHP[τ] + vLP[τ]))
```

```
vLPϕ[τ_] :=
  wLPϕ[τ] ((1 - (p + τ - p τ)) (vHR[τ] + vLR[τ]) + (p + τ - p τ) (vHP[τ] + vLP[τ]))

ωR[τ_] :=
  (1 - d) / ((1 - d) (fHR + fLR) + (p (fHR + fLR) + (1 - p) (fHP + fLP)) d (1 - c))
ωP[τ_] :=
  (1 - d) / ((1 - d) (fHP + fLP) + (p (fHR + fLR) + (1 - p) (fHP + fLP)) d (1 - c))
```

$$SHR[\tau\_] := -\frac{vHR[\tau]}{fHR} cHR + \left(bHR \frac{vHR[\tau]}{fHR} + bLR \frac{vLR[\tau]}{fLR} rR[\tau]\right) - (bHR + bLR - cHR)\ \omega R[\tau]\ (vHR\phi[\tau] + vLR\phi[\tau]\ rR[\tau]);$$

$$SLR[\tau\_] := -\frac{vLR[\tau]}{fLR} cLR + \left(bHR \frac{vHR[\tau]}{fHR} rR[\tau] + bLR \frac{vLR[\tau]}{fLR}\right) - (bHR + bLR - cLR)\ \omega R[\tau]\ (vHR\phi[\tau]\ rR[\tau] + vLR\phi[\tau]);$$

$$SHP[\tau\_] := -\frac{vHP[\tau]}{fHP} cHP + \left(bHP \frac{vHP[\tau]}{fHP} + bLP \frac{vLP[\tau]}{fLP} rP[\tau]\right) - (bHP + bLP - cHP)\ \omega P[\tau]\ (vHP\phi[\tau] + vLP\phi[\tau]\ rP[\tau]);$$

$$SLP[\tau\_] := -\frac{vLP[\tau]}{fLP} cLP + \left(bHP \frac{vHP[\tau]}{fHP} rP[\tau] + bLP \frac{vLP[\tau]}{fLP}\right) - (bHP + bLP - cLP)\ \omega P[\tau]\ (vHP\phi[\tau]\ rP[\tau] + vLP\phi[\tau]);$$

In[9497]:=

```
Clear[zHR, zLR, zHP, zLP]
listHR = {};
listLR = {};
listHP = {};
listLP = {};
zHR = 0.1; zLR = 0.1; zHP = 0.1; zLP = 0.1;
Do[
 aux = N[FindRoot[{SHR[τ] == 0, SLR[τ] == 0, SHP[τ] == 0, SLP[τ] == 0},
    {{xHR, zHR}, {xLR, zLR}, {xHP, zHP}, {xLP, zLP}}]];
 listHR = Append[listHR, xHR /. aux];
 listLR = Append[listLR, xLR /. aux];
 listHP = Append[listHP, xHP /. aux];
 listLP = Append[listLP, xLP /. aux];
 zHR = xHR /. aux;
 zLR = xLR /. aux;
 zHP = xHP /. aux;
 zLP = xLP /. aux
 , {τ, -99/100, 99/100, 1/50}]
harmingHR = ListInterpolation[listHR, {{-99/100, 99/100}}]
harmingLR = ListInterpolation[listLR, {{-99/100, 99/100}}]
harmingHP = ListInterpolation[listHP, {{-99/100, 99/100}}]
harmingLP = ListInterpolation[listLP, {{-99/100, 99/100}}]
```

Out[9504]= InterpolatingFunction[Domain: {{-0.99, 0.99}} Output: scalar]

Out[9505]= InterpolatingFunction[Domain: {{-0.99, 0.99}} Output: scalar]

Out[9506]= InterpolatingFunction[Domain: {{-0.99, 0.99}} Output: scalar]

Out[9507]= InterpolatingFunction[Domain: {{-0.99, 0.99}} Output: scalar]

In[9508]:=

```
colour = {{"#DA3232", "#12588E"}, {"#DA3232", "#12588E"}};
style1 = {Directive[RGBColor[colour[[1]][[1]]], Thickness[0.01]],
   Directive[RGBColor[colour[[1]][[2]]], Dashing[{0.03, 0.05}],
    Thickness[0.012]]};
hlabel = {{-1, "-1.0", {0, 0.01}}, {-0.5, "-0.5", {0, 0.01}},
   {0, "0.0", {0, 0.01}}, {0.5, "0.5", {0, 0.01}},
   {1.0, "1.0", {0, 0.01}}};
vlabel = Join[{{0.0, "0.0", {0, 0.01}}},
   Table[{i, ToString[N[i]], {0, 0.01}}, {i, 2/10, .8, 2/10}],
   {{1, "1.0", {0, 0.01}}}];
pticks = Join[Table[{i, ToString[i], {0, 0.01}}, {i, -0.8, -0.2, 0.2}],
   {{0, "0.0", {0, 0.01}}},
   Table[{i, ToString[i], {0, 0.01}}, {i, 0.2, 0.8, 0.2}]];
vlabelR = Join[{{0.0, "0.0", {0, 0.01}}},
   Table[{i, ToString[N[i]], {0, 0.01}}, {i, 2/10, .8, 2/10}],
   {{1, "1.0", {0, 0.01}}},
   Table[{i, ToString[N[i]], {0, 0.01}}, {i, 12/10, 20/10, 2/10}]];
vlabelP = Join[{{0.0, "0.00", {0, 0.01}}},
   Table[{i, ToString[N[i]], {0, 0.01}}, {i, 2/100, .08, 2/100}],
   {{0.1, "0.10", {0, 0.01}}},
   Table[{i, ToString[N[i]], {0, 0.01}}, {i, 12/100, 20/100, 2/100}]];

Plot[{harmingHR[τ], harmingLR[τ]}, {τ, -1, 1},
 PlotRange → {{-1, 1}, {0, 1}}, Frame → True, AxesOrigin → {-1, 0},
 FrameTicks → {{vlabel, None}, {hlabel, None}},
 FrameLabel → {{Text[Style["competitive effort, z*"]], None},
   {None, None}}, PlotStyle → style1, AspectRatio → 1, ImageSize → 200]
Plot[{harmingHP[τ], harmingLP[τ]}, {τ, -1, 1},
 PlotRange → {{-1, 1}, {0, 1}}, Frame → True, AxesOrigin → {-1, 0},
 FrameTicks → {{vlabel, None}, {hlabel, None}},
 FrameLabel → {{Text[Style["competitive effort, z*"]], None},
   {None, None}}, PlotStyle → style1, AspectRatio → 1, ImageSize → 200]
```

Out[9515]=

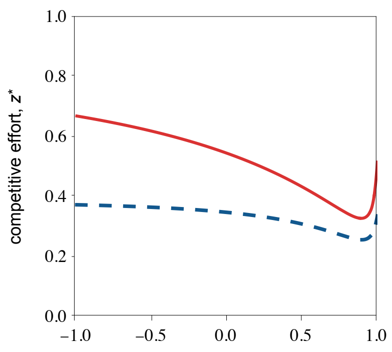

Out[9516]=

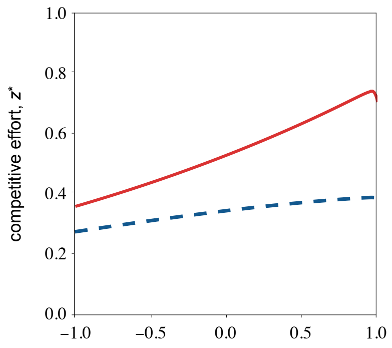

Convergence Stability (CS)

```
c = 0.9;
d = 0.1;
μR = 1;
μP = 0.1;
qHR = 1;
qLR = 0.1;
qHP = 1;
qLP = 0.1;
```

$$fHR := \mu R \left(qHR + \frac{xHR}{\frac{1}{2}(xHR + xLR)} \left(1 - \frac{1}{2}(xHR + xLR)\right)\right);$$

$$fLR := \mu R \left(qLR + \frac{xLR}{\frac{1}{2}(xHR + xLR)} \left(1 - \frac{1}{2}(xHR + xLR)\right)\right);$$

$$fHP := \mu P \left(qHP + \frac{xHP}{\frac{1}{2}(xHP + xLP)} \left(1 - \frac{1}{2}(xHP + xLP)\right)\right);$$

$$fLP := \mu P \left(qLP + \frac{xLP}{\frac{1}{2}(xHP + xLP)} \left(1 - \frac{1}{2}(xHP + xLP)\right)\right);$$

$$sR = \frac{fLR}{fHR};\ sP = \frac{fLP}{fHP};\ \sigma = \frac{fHP}{fHR};$$

$$cHR := -\frac{1}{\frac{1}{2}}\left(1 - \frac{1}{2}(xHR + xLR)\right);$$

$$bHR := -\frac{xHR}{\frac{1}{2}(xHR + xLR)};$$

$$cLR := -\frac{1}{\frac{1}{2}}\left(1 - \frac{1}{2}(xHR + xLR)\right);$$

$$bLR := -\frac{xLR}{\frac{1}{2}(xHR + xLR)};$$

$$cHP := -\frac{1}{\frac{1}{2}}\left(1 - \frac{1}{2}(xHP + xLP)\right);$$

$$bHP := -\frac{xHP}{\frac{1}{2}(xHP + xLP)};$$

$$cLP := -\frac{1}{\frac{1}{2}}\left(1 - \frac{1}{2}(xHP + xLP)\right);$$

$$bLP := -\frac{xLP}{\frac{1}{2}(xHP + xLP)};$$

$vHR[\tau\_] := 1$

$vLR[\tau\_] :=$

$\Big(sR$

$\quad\big(-2\ (1 + sP)\ \sigma\ (-1 + \tau) -$

$\quad\quad 2\ d\ (-1 - sR + \sigma + sP\ \sigma + c\ (1 + sR - (1 + sP)\ \sigma\ (-1 + \tau)) - \sigma\ \tau - sP\ \sigma\ \tau) +$

$\quad\quad d^2\ \big(-1 - sR + \sigma + sP\ \sigma + c^2\ (1 + sR + \sigma + sP\ \sigma) - 2\ c\ (1 + sP)\ \sigma\ \tau\big)\big)\Big) \Big/$

$\quad\Big(sP\ \sigma$

$\quad\big(-2\ (1 + sR)\ (-1 + \tau) +$

$\quad\quad d^2\ \big(1 + sR - \sigma - sP\ \sigma + c^2\ (1 + sR + \sigma + sP\ \sigma) - 2\ c\ (1 + sR)\ \tau\big) +$

$\quad\quad 2\ d\ (-1 + \sigma + sP\ \sigma + sR\ (-1 + \tau) + \tau - c\ (1 + sR + \sigma + sP\ \sigma - \tau - sR\ \tau))\big)\Big) \Big/$

$\big(-2\ (1 + sP)\ \sigma\ (-1 + \tau) -$

$\quad 2\ d\ (-1 - sR + \sigma + sP\ \sigma + c\ (1 + sR - (1 + sP)\ \sigma\ (-1 + \tau)) - \sigma\ \tau - sP\ \sigma\ \tau) +$

$\quad d^2\ \big(-1 - sR + \sigma + sP\ \sigma + c^2\ (1 + sR + \sigma + sP\ \sigma) - 2\ c\ (1 + sP)\ \sigma\ \tau\big)\big) \Big/$

```
    (sP σ
      (-2 (1 + sR) (-1 + τ) +
        d^2 (1 + sR - σ - sP σ + c^2 (1 + sR + σ + sP σ) - 2 c (1 + sR) τ) +
        2 d (-1 + σ + sP σ + sR (-1 + τ) + τ - c (1 + sR + σ + sP σ - τ - sR τ))))
vHP[τ_] :=
  1
  --/
  sP
   (-2 (1 + sP) σ (-1 + τ) -
      2 d (-1 - sR + σ + sP σ + c (1 + sR - (1 + sP) σ (-1 + τ)) - σ τ - sP σ τ) +
      d^2 (-1 - sR + σ + sP σ + c^2 (1 + sR + σ + sP σ) - 2 c (1 + sP) σ τ)) /
    (sP σ
      (-2 (1 + sR) (-1 + τ) +
        d^2 (1 + sR - σ - sP σ + c^2 (1 + sR + σ + sP σ) - 2 c (1 + sR) τ) +
        2 d (-1 + σ + sP σ + sR (-1 + τ) + τ - c (1 + sR + σ + sP σ - τ - sR τ))))
vLP[τ_] :=
  1 /
   (-2 (1 + sP) σ (-1 + τ) -
      2 d (-1 - sR + σ + sP σ + c (1 + sR - (1 + sP) σ (-1 + τ)) - σ τ - sP σ τ) +
      d^2 (-1 - sR + σ + sP σ + c^2 (1 + sR + σ + sP σ) - 2 c (1 + sP) σ τ)) /
    (sP σ
      (-2 (1 + sR) (-1 + τ) +
        d^2 (1 + sR - σ - sP σ + c^2 (1 + sR + σ + sP σ) - 2 c (1 + sR) τ) +
        2 d (-1 + σ + sP σ + sR (-1 + τ) + τ - c (1 + sR + σ + sP σ - τ - sR τ))))

wHRϕ[τ_] :=
  ((1 - d) (fHR)) /
   ((1 - d) (fHR + fLR) + (p (fHR + fLR) + (1 - p) (fHP + fLP)) d (1 - c))
wLRϕ[τ_] :=
  ((1 - d) (fLR)) /
   ((1 - d) (fHR + fLR) + (p (fHR + fLR) + (1 - p) (fHP + fLP)) d (1 - c))
wHPϕ[τ_] :=
  ((1 - d) (fHP)) /
   ((1 - d) (fHP + fLP) + (p (fHR + fLR) + (1 - p) (fHP + fLP)) d (1 - c))
wLPϕ[τ_] :=
  ((1 - d) (fLP)) /
   ((1 - d) (fHP + fLP) + (p (fHR + fLR) + (1 - p) (fHP + fLP)) d (1 - c))

vHRϕ[τ_] :=
  wHRϕ[τ] ((1 - p + p τ) (vHR[τ] + vLR[τ]) + (1 - (1 - p + p τ)) (vHP[τ] + vLP[τ]))
vLRϕ[τ_] :=
  wLRϕ[τ] ((1 - p + p τ) (vHR[τ] + vLR[τ]) + (1 - (1 - p + p τ)) (vHP[τ] + vLP[τ]))
```

```
vHPϕ[τ_] :=
 wHPϕ[τ] ((1 - (p + τ - p τ)) (vHR[τ] + vLR[τ]) + (p + τ - p τ) (vHP[τ] + vLP[τ]))
vLPϕ[τ_] :=
 wLPϕ[τ] ((1 - (p + τ - p τ)) (vHR[τ] + vLR[τ]) + (p + τ - p τ) (vHP[τ] + vLP[τ]))

ωR[τ_] :=
 (1 - d) / ((1 - d) (fHR + fLR) + (p (fHR + fLR) + (1 - p) (fHP + fLP)) d (1 - c))
ωP[τ_] :=
 (1 - d) / ((1 - d) (fHP + fLP) + (p (fHR + fLR) + (1 - p) (fHP + fLP)) d (1 - c))
```

$$SHR[\tau\_] := -\frac{vHR[\tau]}{fHR} cHR + \left(bHR \frac{vHR[\tau]}{fHR} + bLR \frac{vLR[\tau]}{fLR} rR[\tau]\right) - (bHR + bLR - cHR)\, \omega R[\tau]\, (vHR\phi[\tau] + vLR\phi[\tau]\, rR[\tau]);$$

$$SLR[\tau\_] := -\frac{vLR[\tau]}{fLR} cLR + \left(bHR \frac{vHR[\tau]}{fHR} rR[\tau] + bLR \frac{vLR[\tau]}{fLR}\right) - (bHR + bLR - cLR)\, \omega R[\tau]\, (vHR\phi[\tau]\, rR[\tau] + vLR\phi[\tau]);$$

$$SHP[\tau\_] := -\frac{vHP[\tau]}{fHP} cHP + \left(bHP \frac{vHP[\tau]}{fHP} + bLP \frac{vLP[\tau]}{fLP} rP[\tau]\right) - (bHP + bLP - cHP)\, \omega P[\tau]\, (vHP\phi[\tau] + vLP\phi[\tau]\, rP[\tau]);$$

$$SLP[\tau\_] := -\frac{vLP[\tau]}{fLP} cLP + \left(bHP \frac{vHP[\tau]}{fHP} rP[\tau] + bLP \frac{vLP[\tau]}{fLP}\right) - (bHP + bLP - cLP)\, \omega P[\tau]\, (vHP\phi[\tau]\, rP[\tau] + vLP\phi[\tau]);$$

$$eig = Table\Big[Re\Big[Eigenvalues\Big[\begin{pmatrix} D[SHR[\tau], xHR] & D[SHR[\tau], xLR] & D[SHR[\tau], xHP] & D[SHR[\tau], xLP] \\ D[SLR[\tau], xHR] & D[SLR[\tau], xLR] & D[SLR[\tau], xHP] & D[SLR[\tau], xLP] \\ D[SHP[\tau], xHR] & D[SHP[\tau], xLR] & D[SHP[\tau], xHP] & D[SHP[\tau], xLP] \\ D[SLP[\tau], xHR] & D[SLP[\tau], xLR] & D[SLP[\tau], xHP] & D[SLP[\tau], xLP] \end{pmatrix} /. \{xHR \to harmingHR[\tau], xLR \to harmingLR[\tau], xHP \to harmingHP[\tau], xLP \to harmingLP[\tau]\}\Big]\Big], \left\{\tau, -\frac{99}{100}, \frac{99}{100}, \frac{1}{50}\right\}\Big]$$

```
Out[9549]= {{-0.732379, -0.732379, -0.121681, -0.121681},
  {-0.736732, -0.736732, -0.121595, -0.121595},
  {-0.74114, -0.74114, -0.121506, -0.121506},
  {-0.745604, -0.745604, -0.121415, -0.121415},
  {-0.750125, -0.750125, -0.121321, -0.121321},
  {-0.754705, -0.754705, -0.121225, -0.121225},
```

```
{-0.759343, -0.759343, -0.121125, -0.121125},
{-0.764041, -0.764041, -0.121023, -0.121023},
{-0.7688, -0.7688, -0.120917, -0.120917},
{-0.773619, -0.773619, -0.120809, -0.120809},
{-0.778501, -0.778501, -0.120697, -0.120697},
{-0.783446, -0.783446, -0.120582, -0.120582},
{-0.788454, -0.788454, -0.120464, -0.120464},
{-0.793527, -0.793527, -0.120343, -0.120343},
{-0.798666, -0.798666, -0.120218, -0.120218},
{-0.803871, -0.803871, -0.12009, -0.12009},
{-0.809143, -0.809143, -0.119958, -0.119958},
{-0.814483, -0.814483, -0.119822, -0.119822},
{-0.819891, -0.819891, -0.119683, -0.119683},
{-0.825369, -0.825369, -0.119539, -0.119539},
{-0.830918, -0.830918, -0.119392, -0.119392},
{-0.836538, -0.836538, -0.11924, -0.11924},
{-0.842229, -0.842229, -0.119084, -0.119084},
{-0.847994, -0.847994, -0.118924, -0.118924},
{-0.853831, -0.853831, -0.11876, -0.11876},
{-0.859743, -0.859743, -0.118591, -0.118591},
{-0.86573, -0.86573, -0.118417, -0.118417},
{-0.871792, -0.871792, -0.118238, -0.118238},
{-0.87793, -0.87793, -0.118054, -0.118054},
{-0.884145, -0.884145, -0.117865, -0.117865},
{-0.890437, -0.890437, -0.117671, -0.117671},
{-0.896806, -0.896806, -0.117471, -0.117471},
{-0.903254, -0.903254, -0.117266, -0.117266},
{-0.90978, -0.90978, -0.117055, -0.117055},
{-0.916384, -0.916384, -0.116838, -0.116838},
{-0.923068, -0.923068, -0.116615, -0.116615},
{-0.92983, -0.92983, -0.116385, -0.116385},
{-0.936671, -0.936671, -0.116149, -0.116149},
{-0.943591, -0.943591, -0.115906, -0.115906},
{-0.950589, -0.950589, -0.115657, -0.115657},
{-0.957666, -0.957666, -0.115399, -0.115399},
{-0.96482, -0.96482, -0.115135, -0.115135},
{-0.97205, -0.97205, -0.114862, -0.114862},
{-0.979356, -0.979356, -0.114582, -0.114582},
{-0.986737, -0.986737, -0.114293, -0.114293},
{-0.994191, -0.994191, -0.113996, -0.113996},
{-1.00172, -1.00172, -0.113689, -0.113689},
{-1.00931, -1.00931, -0.113373, -0.113373},
{-1.01697, -1.01697, -0.113048, -0.113048},
{-1.0247, -1.0247, -0.112712, -0.112712},
{-1.03248, -1.03248, -0.112366, -0.112366},
{-1.04033, -1.04033, -0.112009, -0.112009},
{-1.04822, -1.04822, -0.111641, -0.111641},
{-1.05617, -1.05617, -0.11126, -0.11126},
{-1.06416, -1.06416, -0.110867, -0.110867},
{-1.07218, -1.07218, -0.110461, -0.110461},
{-1.08023, -1.08023, -0.110041, -0.110041},
```

```
{-1.0883, -1.0883, -0.109607, -0.109607},
{-1.09639, -1.09639, -0.109158, -0.109158},
{-1.10447, -1.10447, -0.108693, -0.108693},
{-1.11255, -1.11255, -0.108211, -0.108211},
{-1.1206, -1.1206, -0.107712, -0.107712},
{-1.12861, -1.12861, -0.107193, -0.107193},
{-1.13656, -1.13656, -0.106656, -0.106656},
{-1.14444, -1.14444, -0.106097, -0.106097},
{-1.15222, -1.15222, -0.105517, -0.105517},
{-1.15988, -1.15988, -0.104913, -0.104913},
{-1.16739, -1.16739, -0.104284, -0.104284},
{-1.17472, -1.17472, -0.103628, -0.103628},
{-1.18182, -1.18182, -0.102944, -0.102944},
{-1.18867, -1.18867, -0.102229, -0.102229},
{-1.19521, -1.19521, -0.101481, -0.101481},
{-1.20139, -1.20139, -0.100699, -0.100699},
{-1.20715, -1.20715, -0.0998779, -0.0998779},
{-1.21241, -1.21241, -0.099016, -0.099016},
{-1.2171, -1.2171, -0.0981096, -0.0981096},
{-1.22113, -1.22113, -0.0971547, -0.0971547},
{-1.22438, -1.22438, -0.096147, -0.096147},
{-1.22673, -1.22673, -0.0950815, -0.0950815},
{-1.22805, -1.22805, -0.0939525, -0.0939525},
{-1.22815, -1.22815, -0.0927536, -0.0927536},
{-1.22685, -1.22685, -0.0914773, -0.0914773},
{-1.22392, -1.22392, -0.0901152, -0.0901152},
{-1.21908, -1.21908, -0.0886573, -0.0886573},
{-1.21201, -1.21201, -0.0870921, -0.0870921},
{-1.20234, -1.20234, -0.0854061, -0.0854061},
{-1.1896, -1.1896, -0.0835834, -0.0835834},
{-1.17324, -1.17324, -0.081605, -0.081605},
{-1.15261, -1.15261, -0.0794481, -0.0794481},
{-1.12687, -1.12687, -0.0770855, -0.0770855},
{-1.09504, -1.09504, -0.0744838, -0.0744838},
{-1.05586, -1.05586, -0.0716018, -0.0716018},
{-1.00777, -1.00777, -0.0683887, -0.0683887},
{-0.948793, -0.948793, -0.06478, -0.06478},
{-0.876372, -0.876372, -0.0606936, -0.0606936},
{-0.78719, -0.78719, -0.0560229, -0.0560229},
{-0.676856, -0.676856, -0.050627, -0.050627},
{-0.539465, -0.539465, -0.0443156, -0.0443156},
{-0.366977, -0.366977, -0.0368241, -0.0368241},
{-0.148578, -0.148578, -0.0277734, -0.0277734}}
```

```
In[9550]:= ListLinePlot[Transpose[eig], PlotRange → {-2, 2}, Frame → True,
  FrameLabel →
   {{Text[Style["eigenvalues", FontSize → 12, FontFamily → "Arial"]],
     None}, {None, None}}]
```

Out[9550]=

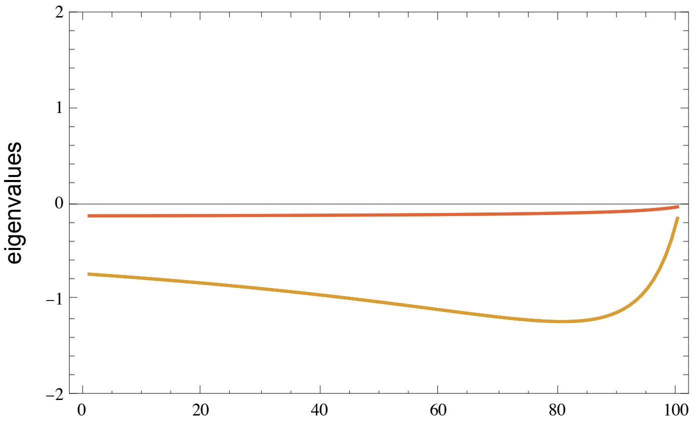

patch size, $n = 4$

code

```
In[1]:= Clear[w, vn, v, pn, p, rn, r, A, fecundity, z, cost, benefit,
          social, dyna, Smatrix, P, f, wϕ, wδ]
        n = 4; (* patch size *)
        a = 2; (* number of high-quality breeders *)
        t = 2; (* two qualities of individuals *)
        NP = 2; (* patch types - resource-rich and resource-poor *)
        tmax = 800;
        c = 5/10; (* cost of dispersal *)
        d = 1/10; (* dispersal rate *)
        GI[i_, k_] := If[i == 1, 1, 1/10] (* baseline fecundity *)
        GP[1] := 1; (* resource-availability in resource-rich patches *)
        GP[2] := 1/10; (* resource-availability in resource-poor patches *)

        cost[i_, q_] := - (1 - 1/n Sum[a z[[q]][[j]], {j, 1, t}]) / (1/n) (* cost of the behaviour *)

        benefit[i_, q_] := - n z[[q]][[i]] / Sum[a z[[q]][[j]], {j, 1, t}] (* benefit of the behaviour *)

        z = Table[1/10, {q, 1, NP}, {i, 1, t}]
        (* initial competitive effort values *)
        dyna = {z};

        f[i_, k_] :=
          GP[k] * (GI[i, k] + z[[k]][[i]] / (1/n Sum[(a z[[k]][[j]]), {j, 1, t}]) (1 - 1/n Sum[(a z[[k]][[j]]), {j, 1, t}]))
        (* fecundity of each individual *)

        upperH = {}; (* auxiliary vectors *)
        lowerH = {};
        upperL = {};
        lowerL = {};

        upperVH = {};
        lowerVH = {};
```

```
upperVL = {};
lowerVL = {};

upperZH = {};
lowerZH = {};
upperZL = {};
lowerZL = {};

rH = {};
rL = {};
```

$\theta = \frac{1}{2}$; (* proportion of resource-rich patches *)

$\tau = -\frac{99}{100}$; (* initial value of temporal correlation *)

Do[

$$P = \begin{pmatrix} \theta + \tau - \theta\,\tau & 1 - (\theta + \tau - \theta\,\tau) \\ 1 - (1 - \theta + \theta\,\tau) & 1 - \theta + \theta\,\tau \end{pmatrix};$$

Do[

$$pn[i\_] := \sum_{j=1}^{NP} p[j]\, P[[i]][[j]];$$

$$solp = NSolve\left[Join\left[Table[pn[i] == p[i], \{i, 1, NP\}], \left\{\left(\sum_{i=1}^{NP} p[i]\right) == 1\right\}\right], Table[p[i], \{i, 1, NP\}]\right][[1]];$$

```
Table[{p[i] = p[i] /. solp}, {i, 1, NP}];
```

$$w\phi[i\_, j\_] := ((1-d)\, f[i, j]) \Big/ \left(\left(\sum_{l=1}^{t} a\, f[l, j]\right) (1-d) + \left(\sum_{k=1}^{NP} p[k] \sum_{l=1}^{t} a\, f[l, k]\right) d\, (1-c)\right);$$

$$w\delta[i\_, j\_, e\_] := (f[i, j]\, d\, (1-c)) \Big/$$

$$\left(\left(\sum_{l=1}^{t} a\, f[l, e]\right)(1-d) + \left(\sum_{k=1}^{NP} p[k] \sum_{l=1}^{t} a\, f[l, k]\right) d\,(1-c)\right);$$

w[i_, j_, u_, q_] :=

$$w\phi[i, j]\; P[[j]][[q]] + \sum_{e=1}^{NP} p[e]\; w\delta[i, j, e]\; P[[e]][[q]];$$

$$vn[i\_, j\_] := If\left[i == j == 1, 1, \sum_{q=1}^{NP}\sum_{u=1}^{t} (w[i, j, u, q]\; a\, v[u, q])\right];$$

```
solrv = NSolve[Flatten[Table[vn[i, q] == v[i, q], {i, 1, t}, {q, 1, NP}]],
   Flatten[Table[v[i, q], {i, 1, t}, {q, 1, NP}]]][[1]];

Table[{v[i, q] = v[i, q] /. solrv}, {i, 1, t}, {q, 1, NP}];
```

ω[i_, q_] :=

$$(1-d) \Big/ \left(\left(\sum_{l=1}^{t} a\, f[l, q]\right)(1-d) + \left(\sum_{k=1}^{NP} p[k] \sum_{l=1}^{t} a\, f[l, k]\right) d\,(1-c)\right);$$

$$vp[y\_, e\_] := \sum_{q=1}^{NP}\sum_{u=1}^{t} w\phi[y, e]\; a\, P[[e]][[q]]\; v[u, q];$$

φ[j_] :=

$$\left(\left(\left(\sum_{i=1}^{t} (a\, f[i, j])\right)(1-d)\right) \Big/ \left(\left(\sum_{i=1}^{t} (a\, f[i, j])\right)(1-d) + \left(\sum_{k=1}^{NP} p[k] \sum_{i=1}^{t} (a\, f[i, k])\right) d\,(1-c)\right)\right)^{2};$$

rn[q_] :=

$$\sum_{k=1}^{NP} P[[k]][[q]]\; \varphi[k] \left(\left(a \sum_{l=1}^{t} \left(\frac{f[l, k]}{\sum_{e=1}^{t} a\, f[e, k]}\right)^{2}\right) + \left(1 - \left(a \sum_{l=1}^{t} \left(\left(\frac{f[l, k]}{\sum_{e=1}^{t} a\, f[e, k]}\right)^{2}\right)\right)\right) r[k]\right);$$

```
solg = NSolve[Flatten[Table[rn[q] == r[q], {q, 1, NP}]],
   Flatten[Table[r[q], {q, 1, NP}]]];
Table[{r[q] = r[q] /. solg[[1]]}, {q, 1, NP}];
```

```
ρ[i_, k_, q_] := If[i == k, 1/a + (a - 1)/a r[q], r[q]];

S[i_, q_] := -(v[i, q]/f[i, q]) cost[i, q] + Sum[a benefit[k, q] (v[k, q]/f[k, q]) ρ[i, k, q], {k, 1, t}] -
   (Sum[benefit[k, q] a ω[k, q], {k, 1, t}]) (Sum[a vp[l, q] ρ[i, l, q], {l, 1, t}]) +
   cost[i, q] ω[i, q] (Sum[a vp[k, q] ρ[i, k, q], {k, 1, t}]);

z =
  Clip[
   Table[N[Rationalize[z[[q]][[i]] + 0.0015 * Sign[S[i, q]], 0.00001]],
    {q, 1, NP}, {i, 1, t}], {0.0, 1.0}];

dyna = Append[dyna, z];
Clear[p, pn, r, rn, v, vn, ρ];

If[asd > 19, Break[]], {σ, tmax}];

upperZH = Append[upperZH, {τ, z[[1]][[1]]}];
lowerZH = Append[lowerZH, {τ, z[[1]][[2]]}];
upperZL = Append[upperZL, {τ, z[[2]][[1]]}];
lowerZL = Append[lowerZL, {τ, z[[2]][[2]]}];
Print[τ],

{τ, -99/100 + 10/100, 99/100, 10/100}]
```

Out[11]= $\left\{\left\{\frac{1}{10}, \frac{1}{10}\right\}, \left\{\frac{1}{10}, \frac{1}{10}\right\}\right\}$

plot - optimal competitive effort strategies

In[136]:=
```
panel1 = ListLinePlot[{upperZH, lowerZH},
  PlotRange → {{-1.01, 1.01}, {-0.03, 1.03}}, Frame → True,
  AxesOrigin → {-1, 0}, BaseStyle → {FontSize → 10, FontFamily → "Arial"},
  FrameLabel →
   {{Text[Style["competitive effort, zρR", FontSize → 10,
        FontFamily → "Arial"]], None}, {None, None}},
  FrameTicks →
   {{Join[{{0, "0.0", {0, 0.01}}},
      Table[{i, ToString[N[i]], {0, 0.01}}, {i, 0.2, 0.8, 0.2}],
      {{1, "1.0", {0, 0.01}}}], None},
    {{{-1, "-1.0", {0, 0.01}}, {-0.5, "-0.5", {0, 0.01}},
      {0, "0.0", {0, 0.01}}, {0.5, "0.5", {0, 0.01}},
      {1, "1.0", {0, 0.01}}}, None}},
  PlotStyle → {Directive[RGBColor[.9, 0, 0], Dashed, Thickness[0.001]],
    Directive[RGBColor[0, 0, .9], Dashed, Thickness[0.001]]},
  PlotMarkers → {{"●", 8}, {"△", 10}}, ImageSize → 200, AspectRatio → 1]

panel2 = ListLinePlot[{upperZL, lowerZL},
  PlotRange → {{-1.01, 1.01}, {-0.03, 1.03}}, Frame → True,
  AxesOrigin → {-1, 0}, BaseStyle → {FontSize → 10, FontFamily → "Arial"},
  FrameLabel →
   {{Text[Style["competitive effort, zρP", FontSize → 10,
        FontFamily → "Arial"]], None}, {None, None}},
  FrameTicks →
   {{Join[{{0, "0.0", {0, 0.01}}},
      Table[{i, ToString[N[i]], {0, 0.01}}, {i, 0.2, 0.8, 0.2}],
      {{1, "1.0", {0, 0.01}}}], None},
    {{{-1, "-1.0", {0, 0.01}}, {-0.5, "-0.5", {0, 0.01}},
      {0, "0.0", {0, 0.01}}, {0.5, "0.5", {0, 0.01}},
      {1, "1.0", {0, 0.01}}}, None}},
  PlotStyle → {Directive[RGBColor[.9, 0, 0], Dashed, Thickness[0.001]],
    Directive[RGBColor[0, 0, .9], Dashed, Thickness[0.001]]},
  PlotMarkers → {{"●", 8}, {"△", 10}}, ImageSize → 200, AspectRatio → 1]
```

Out[136]=

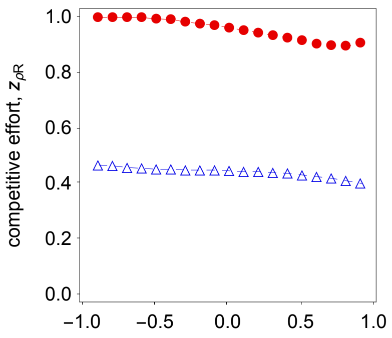

Out[137]=

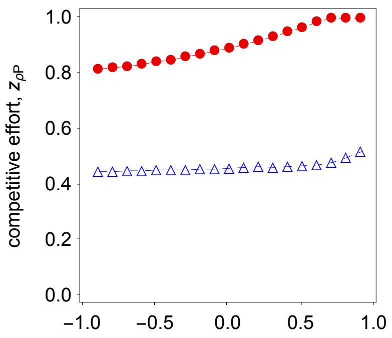


```
upperZH[[All, 2]]
lowerZH[[All, 2]]
```

{0.684874, 0.684874, 0.684874, 0.681905, 0.681922, 0.678922,
0.678922, 0.675926, 0.675926, 0.672922, 0.669935, 0.666937,
0.660944, 0.657971, 0.651961, 0.642981, 0.630952, 0.612943, 0.59194}

{0.153996, 0.153996, 0.156997, 0.153996, 0.156997, 0.153996, 0.154, 0.154004, 0.154,
0.150997, 0.154, 0.154, 0.151007, 0.151007, 0.148, 0.148, 0.145, 0.138996, 0.133005}

plot - inequality within patches

In[138]:=

```
fHR =
 GP[1] *
  (GI[1, 1] + upperZH[[All, 2]] / (1/n (a upperZH[[All, 2]] + a lowerZH[[All, 2]]))
     (1 - 1/n (a upperZH[[All, 2]] + a lowerZH[[All, 2]])))

fLR =
 GP[1] *
  (GI[2, 1] + lowerZH[[All, 2]] / (1/n (a upperZH[[All, 2]] + a lowerZH[[All, 2]]))
     (1 - 1/n (a upperZH[[All, 2]] + a lowerZH[[All, 2]])))

fHP =
 GP[2] *
  (GI[1, 2] + upperZL[[All, 2]] / (1/n (a upperZL[[All, 2]] + a lowerZL[[All, 2]]))
     (1 - 1/n (a upperZL[[All, 2]] + a lowerZL[[All, 2]])))

fLP =
 GP[2] *
```

```
(GI[2, 2] + lowerZL[[All, 2]] / (1/n (a upperZL[[All, 2]] + a lowerZL[[All, 2]]))
    (1 - 1/n (a upperZL[[All, 2]] + a lowerZL[[All, 2]])))
```

```
panel3 = ListLinePlot[{Table[0.9, {i, 1, Length[fLR]}], 1 - fLR/fHR, 1 - fLP/fHP},
  PlotRange → {{0, Length[fLR] + 1}, {0.7, 1}}, Frame → True,
  BaseStyle → {FontSize → 10, FontFamily → "Arial"},
  FrameLabel →
   {{Text[Style["within-group inequality, Gσω", FontSize → 10,
        FontFamily → "Arial"]], None}, {None, None}},
  FrameTicks →
   {{{{0.7, "0.7", {0, 0.01}}, {0.8, "0.8", {0, 0.01}},
      {0.8, "0.8", {0, 0.01}}, {0.9, "0.9", {0, 0.01}},
      {1.0, "1.0", {0, 0.01}}}, None},
    {{{1, "-1.0", {0, 0.01}}, {5.5, "-0.5", {0, 0.01}},
      {10, "0.0", {0, 0.01}}, {14.5, "0.5", {0, 0.01}},
      {19, "1.0", {0, 0.01}}}, None}},
  PlotStyle → {Directive[RGBColor[0, 0, 0], Dotted, Thickness[0.01]],
    Directive[RGBColor[.9, 0, 0], Dashed, Thickness[0.001]],
    Directive[RGBColor[0, 0, .9], Dashed, Thickness[0.001]]},
  PlotMarkers → {{"", 8}, {"●", 8}, {"△", 10}}, ImageSize → 200,
  AspectRatio → 1]
```

```
Out[138]= {1.36162, 1.36441, 1.37, 1.37281, 1.37784, 1.37954,
  1.38745, 1.39082, 1.39417, 1.40203, 1.40987, 1.41476,
  1.42254, 1.43033, 1.4411, 1.45347, 1.46271, 1.47364, 1.47682}

Out[139]= {0.269541, 0.269755, 0.270146, 0.270324, 0.27216, 0.273457,
  0.277509, 0.280143, 0.28281, 0.286949, 0.291096, 0.295216,
  0.299432, 0.303667, 0.307919, 0.313568, 0.316285, 0.31737, 0.31121}

Out[140]= {0.147492, 0.147226, 0.146789, 0.146384, 0.14567, 0.145396,
  0.144831, 0.144101, 0.14352, 0.142783, 0.141736, 0.140835, 0.140341,
  0.139099, 0.138003, 0.136566, 0.135055, 0.133433, 0.131593}

Out[141]= {0.0360413, 0.0357074, 0.0355461, 0.0350503, 0.0345638, 0.0342425,
  0.0336081, 0.0331366, 0.0325203, 0.0320591, 0.0313027, 0.0307044,
  0.0299962, 0.0291378, 0.02843, 0.0274672, 0.0268572, 0.0266791, 0.0264232}
```

Out[142]=

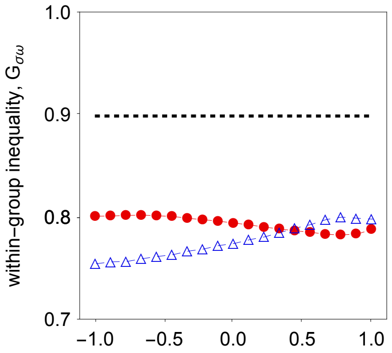

plot - figureI1

In[150]:=

```
figureI1 =
  Show[GraphicsGrid[{{panel1, panel2, panel3}}, Frame → True,
    FrameStyle → White], Frame → True, FrameTicks → None,
   FrameLabel →
    {{None, None},
     {Text[Style["temporal correlation, τ", FontSize → 11,
        FontFamily → "Arial"]], None}}]

Export["figureI1.tiff", figureI1, ImageResolution → 250]
Export["figureI1.pdf", figureI1]
```

Out[150]=

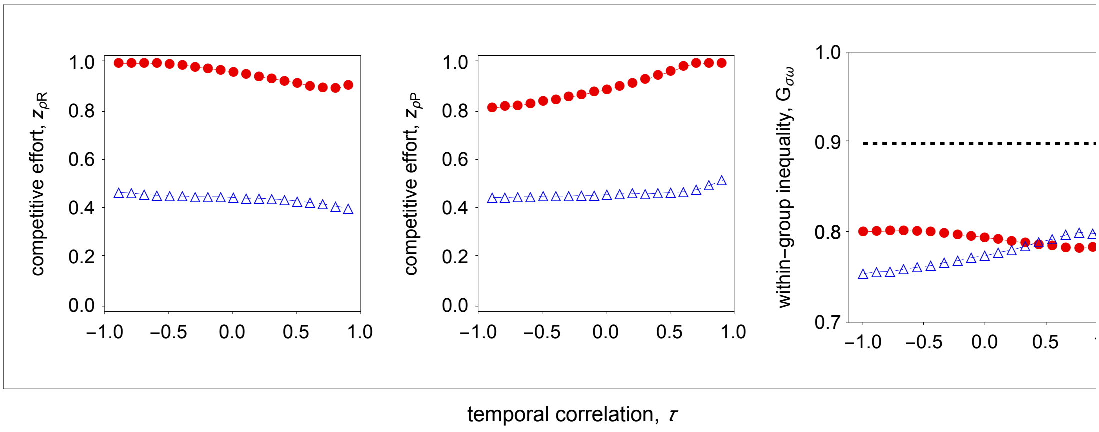

Out[151]= figureI1.tiff

Out[152]= figureI1.pdf

Early-life acquisition of quality

code

```
Clear[q, p, f, d, c, m, i, j, n, w, v, V, r, A, W, w, z, Wϕ, cost,
  benefit, SG]
τ = 0/10;
```

```
tmax = 300;
d = 1/10; c = 1/2; Q = 1/2; n = 2;
GP[1] := 1; GP[2] := 1/10;
GI[1, 1] := 1; GI[2, 1] := 1/10;
GI[1, 2] := 1; GI[2, 2] := 1/10;
z = Table[6/10, {k, 1, 2}, {i, 1, 2}, {j, 1, 2}]

o[1, 1] := 2; o[1, 2] := 1; o[2, 1] := 1; o[2, 2] := 2;

q[1, 1] := Q^2; q[1, 2] := 2 Q (1 - Q); q[2, 1] := 2 Q (1 - Q);
q[2, 2] := (1 - Q)^2;

p[1] := 1/2; p[2] := 1/2;
Prob[1, 1] := τ;
Prob[1, 2] := 1 - τ;
Prob[2, 1] := 1 - τ;
Prob[2, 2] := τ;

dyna = {z};

Do[

 cost[i_, j_, k_] := - (1 - 1/n (z[[k]][[i]][[j]] + z[[k]][[j]][[i]])) / (1/n);

 benefit[i_, j_, k_] := - n z[[k]][[i]][[j]] / (z[[k]][[i]][[j]] + z[[k]][[j]][[i]]);

 f[i_, j_, k_] :=
  GP[k] *
   (GI[i, k] + z[[k]][[i]][[j]] / (1/n (z[[k]][[i]][[j]] + z[[k]][[j]][[i]]))
      (1 - 1/n (z[[k]][[i]][[j]] + z[[k]][[j]][[i]])));
```

```
S := (Sum[p[l] Sum[q[e, u] (f[e, u, l] + f[u, e, l]), {e, 1, 2}, {u, e, 2}], {l, 1, 2}]) d (1 - c);

wϕ[i_, j_, k_] := (f[i, j, k] (1 - d))/((f[i, j, k] + f[j, i, k]) (1 - d) + S);

Wϕ[i_, j_, k_] := (1 - d)/((f[i, j, k] + f[j, i, k]) (1 - d) + S);

wδ[i_, j_, k_, m_, n_, e_] := (f[i, j, k] d (1 - c))/((f[m, n, e] + f[n, m, e]) (1 - d) + S);

w[i_, j_, a_, e_, u_, b_] :=
  (wϕ[i, j, a] Prob[a, b] +
      Sum[p[g] Prob[g, b] (Sum[wδ[i, j, a, l, m, g] q[l, m], {l, 1, 2}, {m, l, 2}]), {g, 1, 2}]) o[e, u]
    q[e, u];

wmatrix =
  Transpose[
   Partition[Flatten[Table[(w[i, j, k, e, u, l]), {k, 1, 2},
      {i, 1, 2}, {j, 1, 2}, {l, 1, 2}, {e, 1, 2}, {u, 1, 2}]], 8]];


vn[i_, j_, a_] :=
  If[i == j == a == 1, 1, Sum[w[i, j, a, e, u, b] v[e, u, b], {b, 1, 2}, {e, 1, 2}, {u, 1, 2}]];

solrv =
  NSolve[Flatten[Table[vn[i, j, a] == v[i, j, a], {a, 1, 2},
       {i, 1, 2}, {j, 1, 2}]],
     Flatten[Table[v[i, j, a], {a, 1, 2}, {i, 1, 2}, {j, 1, 2}]]][[1]];

Table[{v[i, j, a] = v[i, j, a] /. solrv}, {a, 1, 2}, {i, 1, 2},
  {j, 1, 2}];

Flatten[Table[V[i, j, k] = v[i, j, k]/f[i, j, k], {k, 1, 2}, {i, 1, 2}, {j, 1, 2}]];
```

```
rn[i_, j_, k_] :=
 Sum[Prob[g, k]
    (Sum[Sum[q[l, m] (wϕ[l, m, g]^2 + wϕ[m, l, g]^2 +
             2 wϕ[l, m, g] wϕ[m, l, g] r[l, m, g]), {m, l, 2}], {l, 1, 2}]), {g, 1, 2}];

solg = NSolve[{rn[1, 1, 1] == r[1, 1, 1], rn[1, 2, 1] == r[1, 2, 1],
    rn[2, 2, 1] == r[2, 2, 1], rn[1, 1, 2] == r[1, 1, 2],
    rn[1, 2, 2] == r[1, 2, 2], rn[2, 2, 2] == r[2, 2, 2]},
   {r[1, 1, 1], r[1, 2, 1], r[2, 2, 1], r[1, 1, 2], r[1, 2, 2],
    r[2, 2, 2]}];

Table[{r[i, j, k] = r[i, j, k] /. solg[[1]]}, {i, 1, 2}, {j, 1, 2},
  {k, 1, 2}];
r[2, 1, 1] = r[1, 2, 1];
r[2, 1, 2] = r[1, 2, 2];

vϕ[i_, j_, a_] :=
 Sum[Prob[a, g] Sum[Sum[wϕ[i, j, a] o[l, m] q[l, m] v[l, m, g], {m, 1, 2}], {l, 1, 2}], {g, 1, 2}];

A[i_, j_, k_] :=
 (V[j, i, k] r[i, j, k] -
     Wϕ[i, j, k] (vϕ[i, j, k] + vϕ[j, i, k] r[i, j, k])) /
   (V[i, j, k] - Wϕ[i, j, k] (vϕ[i, j, k] + vϕ[j, i, k] r[i, j, k]));

SG[i_, j_, k_] := -V[i, j, k] cost[i, j, k] +
   (benefit[i, j, k] V[i, j, k] + benefit[j, i, k] V[j, i, k] r[i, j, k]) -
   ((benefit[i, j, k] + benefit[j, i, k]) Wϕ[i, j, k]
      (vϕ[i, j, k] + vϕ[j, i, k] r[i, j, k])) +
   cost[i, j, k] Wϕ[i, j, k] (vϕ[i, j, k] + vϕ[j, i, k] r[i, j, k]);

z =
  Clip[
   Table[N[Rationalize[z[[k]][[i]][[j]] + 0.001 * Sign[SG[i, j, k]],
      0.00001]], {k, 1, 2}, {i, 1, 2}, {j, 1, 2}], {0.001, 0.999}];

dyna = Append[dyna, z];
Clear[r, rn, v, vn, rv, v];
```

```
 If[t > 19, Break[]], {σ, tmax}]

z111 = Table[dyna[[t]][[1]][[1]][[1]], {t, 1, Length[dyna]}];
z121 = Table[dyna[[t]][[1]][[1]][[2]], {t, 1, Length[dyna]}];
z211 = Table[dyna[[t]][[1]][[2]][[1]], {t, 1, Length[dyna]}];
z221 = Table[dyna[[t]][[1]][[2]][[2]], {t, 1, Length[dyna]}];
ListLinePlot[{z111, z121, z211, z221}, PlotRange → {0, 1},
 PlotLegends → Automatic]

z112 = Table[dyna[[t]][[2]][[1]][[1]], {t, 1, Length[dyna]}];
z122 = Table[dyna[[t]][[2]][[1]][[2]], {t, 1, Length[dyna]}];
z212 = Table[dyna[[t]][[2]][[2]][[1]], {t, 1, Length[dyna]}];
z222 = Table[dyna[[t]][[2]][[2]][[2]], {t, 1, Length[dyna]}];

ListLinePlot[{z112, z122, z212, z222}, PlotRange → {0, 1},
 PlotLegends → Automatic]

high11 = {Last[z111], Last[z121], Last[z211], Last[z221]}
low11 = {Last[z112], Last[z122], Last[z212], Last[z222]}
```

plot - optimal levels of competitive effort

```
high = {high1, high2a, high2, high3a, high3, high4a, high4, high5a,
   high5, high6a, high6, high7a, high7, high8a, high8, high9a,
   high9, high10a, high10, high11a, high11}
low = {low1, low2a, low2, low3a, low3, low4a, low4, low5a, low5,
   low6a, low6, low7a, low7, low8a, low8, low9a, low9, low10a,
   low10, low11a, low11}
base = Table[0.5, {i, 1, Length[high]}]

fig4A =
 ListLinePlot[{Reverse[Table[high[[i]][[1]], {i, 1, Length[high]}]],
    Reverse[Table[high[[i]][[2]], {i, 1, Length[high]}]],
    Reverse[Table[high[[i]][[3]], {i, 1, Length[high]}]],
    Reverse[Table[high[[i]][[4]], {i, 1, Length[high]}]]},
   PlotRange → {{0.5, Length[high] + 0.5}, {0, 1}}, Frame → True,
   FrameLabel →
    {{Text[Style["competitive effort, z*", FontSize → 11,
        FontFamily → "Arial"]], None}, {None, None}},
   FrameTicks →
    {{{{0, "0.0", {0, 0.01}}, {0.2, "0.2", {0, 0.01}},
       {0.4, "0.4", {0, 0.01}}, {0.6, "0.6", {0, 0.01}},
       {0.8, "0.8", {0, 0.01}}, {1, "1.0", {0, 0.01}}}, None},
     {{{1, "-1.0", {0, 0.01}}, {6, "-0.5", {0, 0.01}},
```

```
      {11, "0.0", {0, 0.01}}, {16, "0.5", {0, 0.01}},
      {21, "1.0", {0, 0.01}}}, None}},
  PlotStyle → {Directive[RGBColor[1, 0, 0], Dashed, Thickness[0.001]],
    Directive[RGBColor[.5, 0, 0], Dashed, Thickness[0.001]],
    Directive[RGBColor[0, 0, .5], Dashed, Thickness[0.001]],
    Directive[RGBColor[0, 0, 1], Dashed, Thickness[0.001]]},
  PlotMarkers → {{"●", 8}, {"■", 8}, {"□", 8}, {"○", 8}},
  AspectRatio → 1, ImageSize → 200]
fig4B =
 ListLinePlot[{Reverse[Table[low[[i]][[1]], {i, 1, Length[low]}]],
   Reverse[Table[low[[i]][[2]], {i, 1, Length[low]}]],
   Reverse[Table[low[[i]][[3]], {i, 1, Length[low]}]],
   Reverse[Table[low[[i]][[4]], {i, 1, Length[low]}]]},
  PlotRange → {{0.5, Length[high] + 0.5}, {0, 1}}, Frame → True,
  FrameLabel →
   {{Text[Style["competitive effort, z*", FontSize → 11,
       FontFamily → "Arial"]], None}, {None, None}},
  FrameTicks →
   {{{{0, "0.0", {0, 0.01}}, {0.2, "0.2", {0, 0.01}},
      {0.4, "0.4", {0, 0.01}}, {0.6, "0.6", {0, 0.01}},
      {0.8, "0.8", {0, 0.01}}, {1, "1.0", {0, 0.01}}}, None},
    {{{1, "-1.0", {0, 0.01}}, {6, "-0.5", {0, 0.01}},
      {11, "0.0", {0, 0.01}}, {16, "0.5", {0, 0.01}},
      {21, "1.0", {0, 0.01}}}, None}},
  PlotStyle → {Directive[RGBColor[1, 0, 0], Dashed, Thickness[0.001]],
    Directive[RGBColor[.5, 0, 0], Dashed, Thickness[0.001]],
    Directive[RGBColor[0, 0, .5], Dashed, Thickness[0.001]],
    Directive[RGBColor[0, 0, 1], Dashed, Thickness[0.001]]},
  PlotMarkers → {{"●", 8}, {"■", 8}, {"□", 8}, {"○", 8}},
  AspectRatio → 1, ImageSize → 200]
```

```
{{0.461905, 0.593857, 0.361905, 0.453947},
 {0.395904, 0.52795, 0.351893, 0.461929}, {0.383901, 0.515957, 0.351893, 0.475962},
 {0.383901, 0.517949, 0.357895, 0.489933}, {0.389937, 0.523952, 0.361905, 0.503968},
 {0.395904, 0.533937, 0.367893, 0.515957}, {0.403922, 0.545894, 0.373874, 0.52795},
 {0.411917, 0.555932, 0.379888, 0.539924}, {0.419913, 0.568, 0.385906, 0.549906},
 {0.425926, 0.58, 0.389908, 0.559965}, {0.433921, 0.589977, 0.393895, 0.567961},
 {0.439904, 0.599828, 0.397906, 0.576037}, {0.445902, 0.609865, 0.401914, 0.584},
 {0.451948, 0.617886, 0.405904, 0.59}, {0.457944, 0.627859, 0.407915, 0.59601},
 {0.46395, 0.635897, 0.411924, 0.60199}, {0.467925, 0.643939, 0.413934, 0.607955},
 {0.473934, 0.649971, 0.415909, 0.613971}, {0.477941, 0.657963, 0.41791, 0.617925},
 {0.481982, 0.664042, 0.419913, 0.621891}, {0.485944, 0.672, 0.421911, 0.625935}}
```

```
{{0.487923, 0.55597, 0.419913, 0.46595},
 {0.617886, 0.708, 0.443925, 0.507979}, {0.609929, 0.689956, 0.437931, 0.493927},
 {0.59596, 0.670034, 0.429936, 0.479943}, {0.583969, 0.647959, 0.423913, 0.46595},
 {0.57, 0.62987, 0.415909, 0.453947}, {0.555891, 0.609877, 0.40991, 0.441926},
 {0.543919, 0.591973, 0.403922, 0.429929}, {0.531943, 0.576, 0.395904, 0.419913},
 {0.519969, 0.55988, 0.389908, 0.407915}, {0.50996, 0.543919, 0.383901, 0.397906},
 {0.497992, 0.529954, 0.377907, 0.389908}, {0.487864, 0.515957, 0.371875, 0.379888},
 {0.477941, 0.501961, 0.365931, 0.371875}, {0.467949, 0.489914, 0.359882, 0.363905},
 {0.45993, 0.477941, 0.353909, 0.355882}, {0.449883, 0.46595, 0.347945, 0.347909},
 {0.441926, 0.455959, 0.343915, 0.339901}, {0.433898, 0.445902, 0.3379, 0.331878},
 {0.425952, 0.435967, 0.331878, 0.325893}, {0.41791, 0.425926, 0.327899, 0.319892}}
```

```
{0.5, 0.5, 0.5, 0.5, 0.5, 0.5, 0.5, 0.5, 0.5,
 0.5, 0.5, 0.5, 0.5, 0.5, 0.5, 0.5, 0.5, 0.5, 0.5, 0.5, 0.5}
```

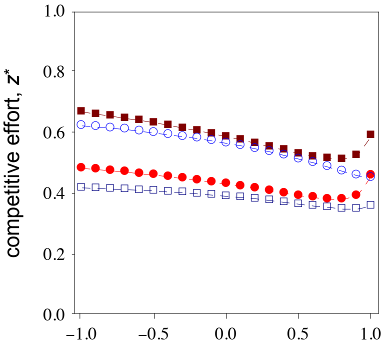

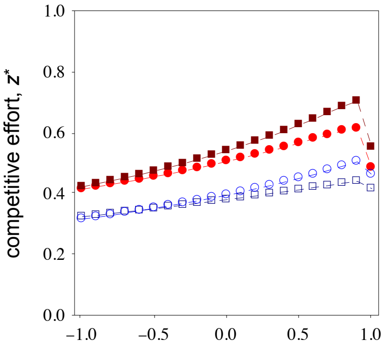

plot - fecundity

```
f111 =
 Table[
  GP[1] *
   (GI[1, 1] + high[[i]][[1]] / (1/2 (high[[i]][[1]] + high[[i]][[1]]))
     (1 - 1/2 (high[[i]][[1]] + high[[i]][[1]]))), {i, 1, Length[high]}]

f121 =
 Table[
  GP[1] *
```

```
(GI[1, 1] + high[[i]][[2]] / (1/2 (high[[i]][[2]] + high[[i]][[3]]))
    (1 - 1/2 (high[[i]][[2]] + high[[i]][[3]]))), {i, 1, Length[high]}]

f211 =
 Table[
  GP[1] *
   (GI[2, 1] + high[[i]][[3]] / (1/2 (high[[i]][[2]] + high[[i]][[3]]))
     (1 - 1/2 (high[[i]][[2]] + high[[i]][[3]]))), {i, 1, Length[high]}]

f221 =
 Table[
  GP[1] *
   (GI[2, 1] + high[[i]][[4]] / (1/2 (high[[i]][[4]] + high[[i]][[4]]))
     (1 - 1/2 (high[[i]][[4]] + high[[i]][[4]]))), {i, 1, Length[high]}]


f112 =
 Table[
  GP[2] *
   (GI[1, 2] + low[[i]][[1]] / (1/2 (low[[i]][[1]] + low[[i]][[1]]))
     (1 - 1/2 (low[[i]][[1]] + low[[i]][[1]]))), {i, 1, Length[low]}]

f122 =
 Table[
  GP[2] *
   (GI[1, 2] + low[[i]][[2]] / (1/2 (low[[i]][[2]] + low[[i]][[3]]))
     (1 - 1/2 (low[[i]][[2]] + low[[i]][[3]]))), {i, 1, Length[low]}]

f212 =
```

```
 Table[
  GP[2] *
   (GI[2, 2] + low[[i]][[3]] / (1/2 (low[[i]][[2]] + low[[i]][[3]]))
       (1 - 1/2 (low[[i]][[2]] + low[[i]][[3]]))), {i, 1, Length[low]}]
f222 =
 Table[
  GP[2] *
   (GI[2, 2] + low[[i]][[4]] / (1/2 (low[[i]][[4]] + low[[i]][[4]]))
       (1 - 1/2 (low[[i]][[4]] + low[[i]][[4]]))), {i, 1, Length[low]}]

fig4C = ListLinePlot[{Reverse[f111], Reverse[f121], Reverse[f211],
    Reverse[f221]}, PlotRange → {{0.5, Length[high] + 0.5}, {0, 2}},
   Frame → True,
   FrameLabel →
    {{Text[Style["fecundity, f", FontSize → 11, FontFamily → "Arial"]],
      None}, {None, None}},
   FrameTicks →
    {{{{0, "0.0", {0, 0.01}}, {0.5, "0.5", {0, 0.01}},
       {1, "1.0", {0, 0.01}}, {1.5, "1.5", {0, 0.01}},
       {2, "2.0", {0, 0.01}}}, None},
     {{{1, "-1.0", {0, 0.01}}, {6, "-0.5", {0, 0.01}},
       {11, "0.0", {0, 0.01}}, {16, "0.5", {0, 0.01}},
       {21, "1.0", {0, 0.01}}}, None}},
   PlotStyle → {Directive[RGBColor[1, 0, 0], Dashed, Thickness[0.001]],
     Directive[RGBColor[.5, 0, 0], Dashed, Thickness[0.001]],
     Directive[RGBColor[0, 0, .5], Dashed, Thickness[0.001]],
     Directive[RGBColor[0, 0, 1], Dashed, Thickness[0.001]]},
   PlotMarkers → {{"●", 8}, {"▲", 9}, {"△", 9}, {"○", 8}},
   AspectRatio → 1, ImageSize → 200]
fig4D = ListLinePlot[{Reverse[f112], Reverse[f122], Reverse[f212],
    Reverse[f222]}, PlotRange → {{0.5, Length[high] + 0.5}, {0, 0.2}},
   Frame → True,
   FrameLabel →
    {{Text[Style["fecundity, f", FontSize → 11, FontFamily → "Arial"]],
      None}, {None, None}},
```

```
FrameTicks →
  {{{{0, "0.00", {0, 0.01}}, {0.05, "0.05", {0, 0.01}},
     {0.1, "0.10", {0, 0.01}}, {0.15, "0.15", {0, 0.01}},
     {0.2, "0.20", {0, 0.01}}}, None},
   {{{1, "-1.0", {0, 0.01}}, {6, "-0.5", {0, 0.01}},
     {11, "0.0", {0, 0.01}}, {16, "0.5", {0, 0.01}},
     {21, "1.0", {0, 0.01}}}, None}},
PlotStyle → {Directive[RGBColor[1, 0, 0], Dashed, Thickness[0.001]],
  Directive[RGBColor[.5, 0, 0], Dashed, Thickness[0.001]],
  Directive[RGBColor[0, 0, .5], Dashed, Thickness[0.001]],
  Directive[RGBColor[0, 0, 1], Dashed, Thickness[0.001]]},
PlotMarkers → {{"●", 8}, {"▲", 9}, {"△", 9}, {"○", 8}},
AspectRatio → 1, ImageSize → 205]
```

```
{1.5381, 1.6041, 1.6161, 1.6161, 1.61006, 1.6041, 1.59608,
 1.58808, 1.58009, 1.57407, 1.56608, 1.5601, 1.5541, 1.54805,
 1.54206, 1.53605, 1.53208, 1.52607, 1.52206, 1.51802, 1.51406}

{1.64883, 1.67215, 1.67309, 1.66479, 1.65898, 1.65018, 1.64113,
 1.63219, 1.62289, 1.61599, 1.60932, 1.60255, 1.59567, 1.58917,
 1.58449, 1.57785, 1.57348, 1.56962, 1.56516, 1.56118, 1.55662}

{0.495407, 0.548006, 0.55906, 0.559363, 0.555168, 0.547988, 0.539101,
 0.531995, 0.523201, 0.514102, 0.506809, 0.499713, 0.492556, 0.48704,
 0.479738, 0.474324, 0.468644, 0.464496, 0.458966, 0.454866, 0.44947}

{0.646053, 0.638071, 0.624038, 0.610067, 0.596032, 0.584043,
 0.57205, 0.560076, 0.550094, 0.540035, 0.532039, 0.523963, 0.516, 0.51,
 0.50399, 0.49801, 0.492045, 0.486029, 0.482075, 0.478109, 0.474065}

{0.151208, 0.138211, 0.139007, 0.140404, 0.141603, 0.143, 0.144411,
 0.145608, 0.146806, 0.148003, 0.149004, 0.150201, 0.151214, 0.152206,
 0.153205, 0.154007, 0.155012, 0.155807, 0.15661, 0.157405, 0.158209}

{0.158345, 0.152125, 0.153349, 0.154824, 0.156106, 0.157472, 0.158621,
 0.159685, 0.16093, 0.161908, 0.162855, 0.163752, 0.164633, 0.165478,
 0.16631, 0.167116, 0.167904, 0.168412, 0.169189, 0.169959, 0.170411}

{0.0540668, 0.0426828, 0.043862, 0.0451787, 0.0467064, 0.0479496, 0.0494003,
 0.0507252, 0.0518794, 0.0531134, 0.0543632, 0.0554615, 0.0565839, 0.0577333,
 0.0587103, 0.0596987, 0.0607067, 0.0616009, 0.0624308, 0.0632562, 0.0642061}

{0.063405, 0.0592021, 0.0606073, 0.0620057, 0.063405, 0.0646053, 0.0658074,
 0.0670071, 0.0680087, 0.0692085, 0.0702094, 0.0710092, 0.0720112, 0.0728125,
 0.0736095, 0.0744118, 0.0752091, 0.0760099, 0.0768122, 0.0774107, 0.0780108}
```

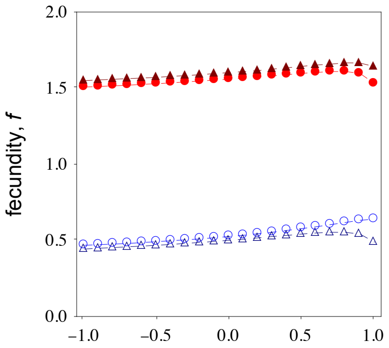
2.0
1.5
1.0
0.5
0.0
fecundity, f
−1.0
−0.5
0.0
0.5
1.0

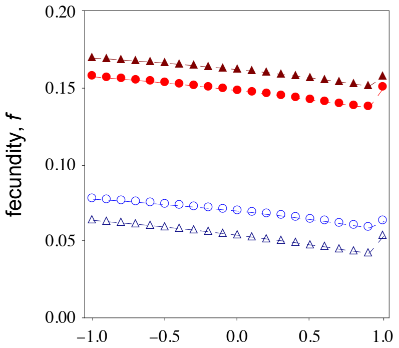
0.20
0.15
0.10
0.05
0.00
fecundity, f
−1.0
−0.5
0.0
0.5
1.0

plot - productivity

```
p111 = 2 f111;
p121 = f121 + f211;
p221 = 2 f221;

p112 = 2 f112;
p122 = f122 + f212;
p222 = 2 f222;

ListLinePlot[{Reverse[p111]/p111[[1]], Reverse[p121]/p121[[1]], Reverse[p221]/p221[[1]]},
 PlotRange → {{0.5, Length[high] + 0.5}, {0.6, 1.4}}, Frame → True,
 FrameTicks → {{Automatic, None},
   {{{1, "-1.0"}, {6, "-0.5"}, {11, "0.0"}, {16, "0.5"}, {21, "1.0"}},
    None}},
 PlotStyle → {Directive[RGBColor[1, 0, 0], Dashed, Thickness[0.001]],
   Directive[RGBColor[.5, 0, 0], Dashed, Thickness[0.001]],
   Directive[RGBColor[0, 0, .5], Dashed, Thickness[0.001]],
   Directive[RGBColor[0, 0, 1], Dashed, Thickness[0.001]]},
 PlotLegends → Automatic,
 PlotMarkers → {{"●", 8}, {"■", 8}, {"□", 8}, {"○", 8}},
 AspectRatio → 1, ImageSize → 200]
ListLinePlot[{Reverse[p112]/p112[[1]], Reverse[p122]/p122[[1]], Reverse[p222]/p222[[1]]},
 PlotRange → {{0.5, Length[high] + 0.5}, {0.6, 1.4}}, Frame → True,
 FrameTicks → {{Automatic, None},
   {{{1, "-1.0"}, {6, "-0.5"}, {11, "0.0"}, {16, "0.5"}, {21, "1.0"}},
    None}},
 PlotStyle → {Directive[RGBColor[1, 0, 0], Dashed, Thickness[0.001]],
   Directive[RGBColor[.5, 0, 0], Dashed, Thickness[0.001]],
   Directive[RGBColor[0, 0, .5], Dashed, Thickness[0.001]],
   Directive[RGBColor[0, 0, 1], Dashed, Thickness[0.001]]},
 PlotLegends → Automatic,
 PlotMarkers → {{"●", 8}, {"■", 8}, {"□", 8}, {"○", 8}},
 AspectRatio → 1, ImageSize → 200]
```

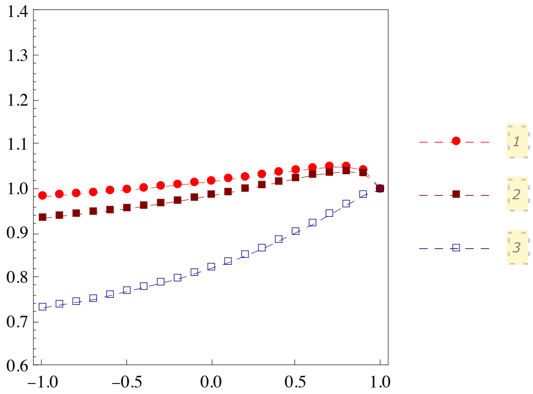
1.4
1.3
1.2
1.1
1.0
0.9
0.8
0.7
0.6
−1.0
−0.5
0.0
0.5
1.0
1
2
3

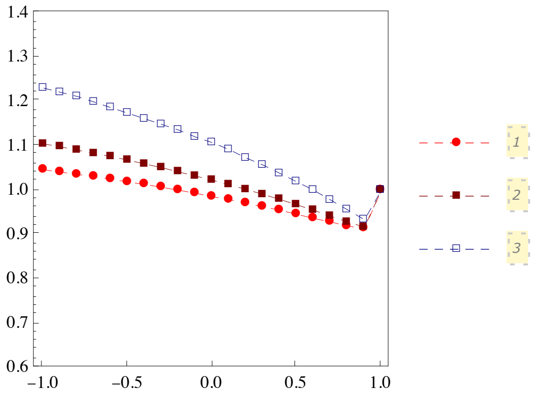
1.4
1.3
1.2
1.1
1.0
0.9
0.8
0.7
0.6
−1.0
−0.5
0.0
0.5
1.0
1
2
3

plot - inequality

```
fig5b =
 ListLinePlot[{Table[0.9, {i, 1, Length[f121]}], Reverse[1 - f211/f121],
   Reverse[1 - f212/f122]}, PlotRange → {{0.5, Length[high] + 0.5}, {0.4, 1}},
  Frame → True,
  FrameLabel →
   {{Text[Style["inequality, Gω", FontSize → 11, FontFamily → "Arial"]],
     None}, {None, None}},
  FrameTicks →
   {{{{0, "0.0", {0, 0.01}}, {0.2, "0.2", {0, 0.01}},
      {0.4, "0.4", {0, 0.01}}, {0.5, "0.5", {0, 0.01}},
      {0.6, "0.6", {0, 0.01}}, {0.7, "0.7", {0, 0.01}},
      {0.8, "0.8", {0, 0.01}}, {0.9, "0.9", {0, 0.01}},
      {1, "1.0", {0, 0.01}}}, None},
    {{{1, "-1.0", {0, 0.01}}, {6, "-0.5", {0, 0.01}},
      {11, "0.0", {0, 0.01}}, {16, "0.5", {0, 0.01}},
      {21, "1.0", {0, 0.01}}}, None}},
  PlotStyle → {Directive[RGBColor[0, 0, 0], Dotted, Thickness[0.015]],
    Directive[RGBColor[.9, 0, 0], Dashed, Thickness[0.001]],
    Directive[RGBColor[0, 0, .9], Dashed, Thickness[0.001]],
    Directive[RGBColor[0, 0, 1], Dashed, Thickness[0.001]]},
  PlotMarkers → {{"", 8}, {"●", 8}, {"△", 10}, {"○", 8}},
  AspectRatio → 1, ImageSize → 200]
```

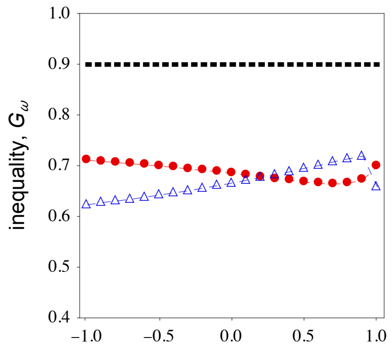

```
GB = 1 - p[2] (q[1, 1] p112 + q[1, 2] p122 + q[2, 2] p222) / p[1] (q[1, 1] p111 + q[1, 2] p121 + q[2, 2] p221)

fig5c = ListLinePlot[{Table[1 - 1/10, {i, 1, Length[GB]}], Reverse[GB]},
  PlotRange → {{0.5, Length[high] + 0.5}, {0.8, 1}}, Frame → True,
  FrameLabel →
   {{Text[Style["inequality, Gβ", FontSize → 11, FontFamily → "Arial"]],
      None}, {None, None}},
  FrameTicks →
   {{{{0, "0.0", {0, 0.01}}, {0.2, "0.2", {0, 0.01}},
       {0.4, "0.4", {0, 0.01}}, {0.5, "0.5", {0, 0.01}},
       {0.6, "0.6", {0, 0.01}}, {0.7, "0.7", {0, 0.01}},
       {0.8, "0.8", {0, 0.01}}, {0.9, "0.9", {0, 0.01}},
       {1, "1.0", {0, 0.01}}}, None},
    {{{1, "-1.0", {0, 0.01}}, {6, "-0.5", {0, 0.01}},
       {11, "0.0", {0, 0.01}}, {16, "0.5", {0, 0.01}},
       {21, "1.0", {0, 0.01}}}, None}},
  PlotStyle → {Directive[RGBColor[0, 0, 0], Dotted, Thickness[0.015]],
    Directive[RGBColor[.9, 0, 0], Dashed, Thickness[0.001]],
    Directive[RGBColor[0, 0, .9], Dashed, Thickness[0.001]],
    Directive[RGBColor[0, 0, 1], Dashed, Thickness[0.001]]},
  PlotMarkers → {{"", 8}, {"●", 8}, {"△", 10}, {"○", 8}},
  AspectRatio → 1, ImageSize → 200]

{0.901343, 0.912104, 0.91127, 0.909577, 0.907738, 0.905837, 0.903817,
 0.901903, 0.900001, 0.898159, 0.896439, 0.894795, 0.89312, 0.891582,
 0.890072, 0.888593, 0.887161, 0.885861, 0.884555, 0.883347, 0.88212}
```

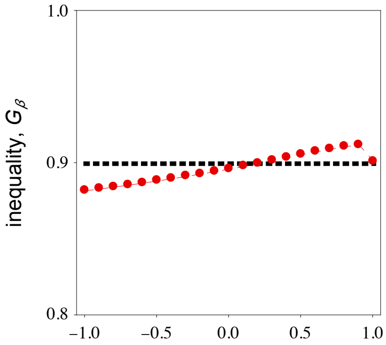

Supplement: Supplemental Information 1 [file peerj-06-5488-s002.pdf]
